# *ACSS2* gene variants determine kidney disease risk by controlling de novo lipogenesis in kidney tubules

**DOI:** 10.1172/JCI172963

**Published:** 2023-12-05

**Authors:** Dhanunjay Mukhi, Lingzhi Li, Hongbo Liu, Tomohito Doke, Lakshmi P. Kolligundla, Eunji Ha, Konstantin Kloetzer, Amin Abedini, Sarmistha Mukherjee, Junnan Wu, Poonam Dhillon, Hailong Hu, Dongyin Guan, Katsuhiko Funai, Kahealani Uehara, Paul M. Titchenell, Joseph A. Baur, Kathryn E. Wellen, Katalin Susztak

**Affiliations:** 1 Renal Electrolyte and Hypertension Division,; 2 Institutes for Diabetes, Obesity and Metabolism,; 3 Department of Genetics, and; 4 Department of Physiology, University of Pennsylvania, Philadelphia, Pennsylvania, USA.; 5 Division of Endocrinology, Baylor College of Medicine, Houston, Texas, USA.; 6 Diabetes and Metabolism Research Center, University of Utah, Salt Lake City, Utah, USA.; 7 Department of Cancer Biology,; 8 Abramson Family Cancer Research Institute, and; 9 Penn-CHOP Kidney Innovation Center, University of Pennsylvania, Philadelphia, Pennsylvania, USA.

**Keywords:** Genetics, Nephrology, Chronic kidney disease, Fibrosis

## Abstract

Worldwide, over 800 million people are affected by kidney disease, yet its pathogenesis remains elusive, hindering the development of novel therapeutics. In this study, we used kidney-specific expression of quantitative traits and single-nucleus open chromatin analysis to show that genetic variants linked to kidney dysfunction on chromosome 20 target the acyl-CoA synthetase short-chain family 2 (*ACSS2*). By generating *ACSS2*-KO mice, we demonstrated their protection from kidney fibrosis in multiple disease models. Our analysis of primary tubular cells revealed that ACSS2 regulated de novo lipogenesis (DNL), causing NADPH depletion and increasing ROS levels, ultimately leading to NLRP3-dependent pyroptosis. Additionally, we discovered that pharmacological inhibition or genetic ablation of fatty acid synthase safeguarded kidney cells against profibrotic gene expression and prevented kidney disease in mice. Lipid accumulation and the expression of genes related to DNL were elevated in the kidneys of patients with fibrosis. Our findings pinpoint ACSS2 as a critical kidney disease gene and reveal the role of DNL in kidney disease.

## Abstract 2

<p>Deletion or inhibition of ACSS2 attenuates fibrosis in chronic kidney disease models by suppressing de novo lipogenesis and NLRP3 inflammasome activation.</p>

## Graphical abstract

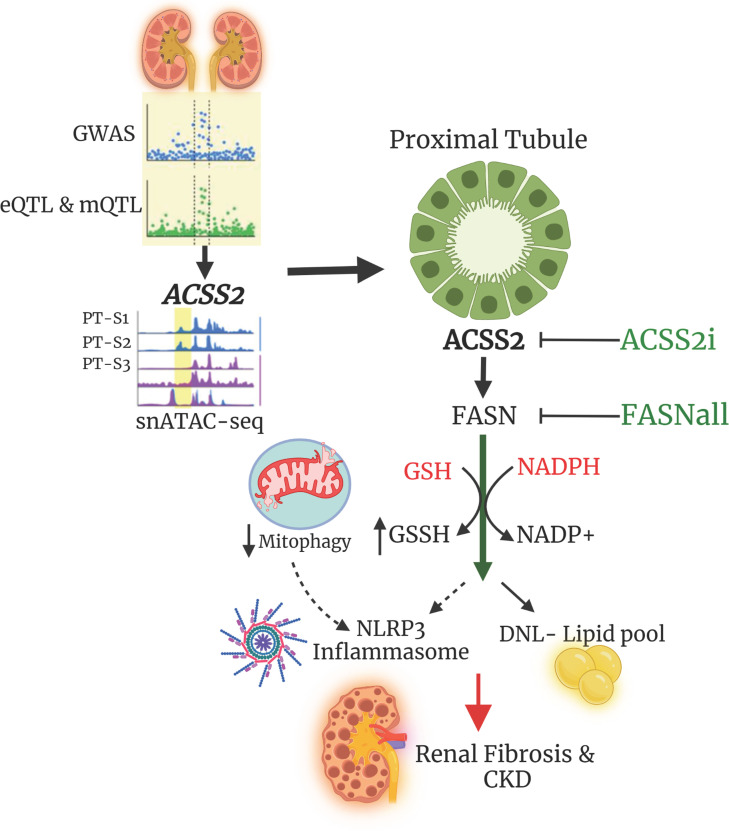

## Introduction

Over 800 million people in the world have chronic kidney disease (CKD) (1). CKD is a major cause of cardiovascular death and if left untreated leads to end-stage kidney disease necessitating dialysis or kidney transplantation. CKD is one of the most rapidly growing common causes of death (2), accounting for over 1 million fatalities each year. Despite its considerable impact on public health, the mechanisms driving CKD pathogenesis remain largely unknown, impeding the development of effective treatments.

The kidney is a highly metabolically active organ responsible for filtering and reabsorbing a vast amount of electrolytes and fluids, including 180 liters of water and nearly 1 kilogram of sodium chloride daily (3, 4). In particular, the proximal tubule (PT) segment of the kidney relies predominantly on fatty acids as an energy source and mitochondrial oxidative phosphorylation for efficient energy production. However, kidney lipid content is markedly elevated in disease states such as diabetic kidney disease (DKD) (5). Indeed, Kimmelstiel and Wilson identified lipid deposition as a key characteristic of DKD (6). The mechanism underlying lipid accumulation and alterations in lipid metabolism in kidney disease, however, remains poorly understood.

Kidney tubule cells take up fatty acids through CD36 and fatty acid transporter protein 2 (FATP2) transporters (7, 8), and increased lipid uptake is believed to contribute to disease development. A defect in fatty acid oxidation (FAO) has been identified as an important contributor to tubule atrophy (9). Improving FAO by expression of *PPARA*, *PPARGC1A*, or *CPT1A* or pharmacological stimulation of these pathways improves kidney function (9–11).

Large-scale GWAS have identified more than 800 genetic loci where SNPs are associated with kidney function as measured by the estimated glomerular filtration rate (eGFR) (12). GWAS, however, have several limitations: most regions contain large number of significant variants whose levels show a close correlation; the identified GWAS variants are in the noncoding region, making it challenging to pinpoint causal variants, target genes, and cell types (13, 14). Functional annotation of GWAS requires multiple orthogonal data sets (14). Recently, expression quantitative trait locus (eQTL) analysis has emerged as a valuable tool for identifying target genes by identifying disease-associated variants that also regulate gene expression (15). Epigenetic data sets including open chromatin annotation have been used to narrow down likely causal variants, and single-cell epigenome and expression data can even identify the disease-causal cell types (16). While computational target gene prioritization has significantly improved, cellular and animal models remain critical for validation studies.

In this study, we conducted a computational analysis of the chromosome 20 eGFR GWAS locus using expression, methylation QTL, and single-cell open chromatin data to prioritize Acyl-CoA synthetase short-chain family 2 (*ACSS2*) as a kidney disease risk gene. Our gene-KO and cellular model experiments revealed the critical role of ACSS2 in de novo lipogenesis (DNL), which consumes NADPH and induces changes in cellular redox homeostasis leading to NLRP3-dependent pyroptosis and fibrosis. Moreover, our mouse model studies demonstrated that pharmacological inhibition of ACSS2 or fatty acid synthase (FASN) can mitigate kidney fibrosis.

## Results

### Gene prioritization analysis indicates that ACSS2 is a kidney disease gene.

**Figure 1:**
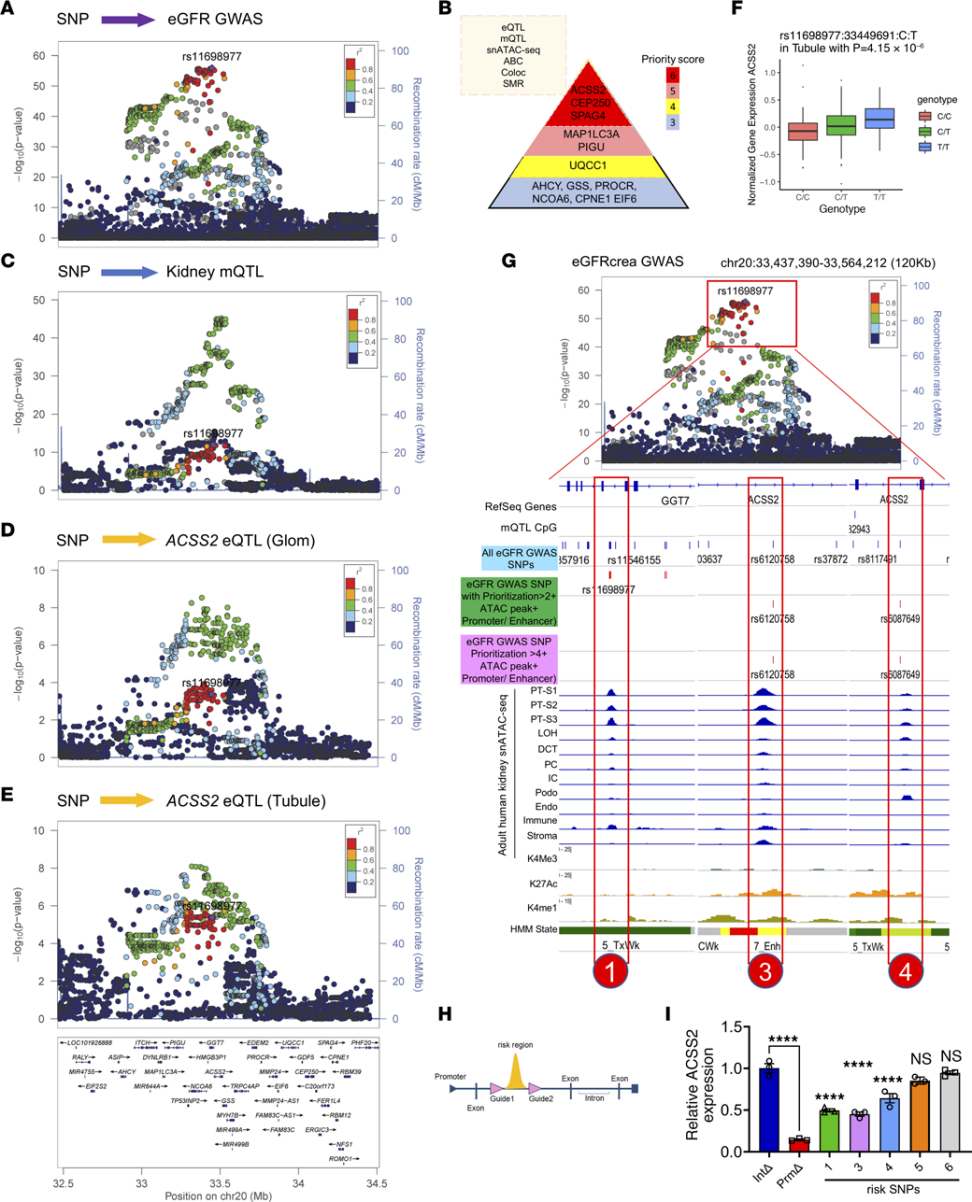
Prioritization of *ACSS2* as a kidney disease gene from GWAS. (**A**) Regional plot showing SNPs associated with kidney eGFR GWAS (*n* = 1,508,569 individuals). The *x* axis shows the chromosomal location, and the *y* axis shows the strength of association [–log(*P*)] on chromosome 20. The locus top variants (rs11698977) tagging the independent signal closest to *ACSS2* gene was selected as the index variant to calculate the linkage disequilibrium (LD) correlation coefficient (r2), with other variants in the locus shown by blue dots (lower r2) and red dots (higher r2). (**B**) Gene prioritization strategy (top left). Genes with gene priority scores higher than 3 at the chromosome 20 eGFR GWAS locus (right). The color indicates the priority score. Coloc, colocalization. (**C**) Regional plot of SNPs associated with kidney tubule cytosine methylation levels (mQTL) (*n* = 443). The *x* axis shows chromosomal location, and the *y* axis shows the strength of association [–log(*P*)]. (**D**) Regional plot for SNPs associated with *ACSS2* expression in kidney glomeruli (*n* = 303). The *x* axis shows the chromosomal location, and the *y* axis shows the strength of association [–log(*P*)]. (**E**) Regional plot for SNPs associated with kidney tubule *ACSS2* expression (*n* = 356). The *x* axis shows the chromosomal (chr) location, and the *y* axis shows the strength of association [–log(*P*)]. (**F**) Human kidney *ACSS2* gene expression in tubules (*n* = 356) in microdissected samples. The *y* axis shows normalized *ACSS2* expression, and the *x* axis shows genotype information. (**G**) Upper panel: Locus zoom plot of eGFRcrea GWAS associations (*n* = 1,508,659 individuals; the same is shown in **A**) in the ACSS2 locus. Lower panel: Epigenetic information on the ACSS2 locus in human kidney samples including mQTL SNPs; all eGFR GWAS SNPs (blue) followed by eGFR GWAS SNPs with a priority score of greater than 2 (dark green); eGFRcrea GWAS SNPs with a priority score of greater than 4 (magenta); adult human kidney open chromatin information for each cell type; and histone modifications (H34me3, H3K27ac, and H3K4me1) from human kidney ChIP-Seq and chromatin states predicted by ChromHMM. PT-S1, PT segment S1; LOH, loop of Henle; DCT, distal convoluted tubule; PC, collecting duct principal cells; IC, collecting duct intercalated cells; Podo, podocytes; Endo, endothelial cells; Immune, immune cells; Stroma, stromal fraction. (**H**) Scheme of CRISPR-mediated genomic region deletion. (**I**) Transcript levels of *ACSS2* following deletion of the genetic risk locus containing SNPs 1, 3, 4, 5, and 6. Int, intron; Prm, promoter. Data are presented as the mean ± SEM. ****P < 0.0001, by 1-way ANOVA after Tukey’s multiple-comparison test.

A recent GWAS of kidney function (eGFRcrea) revealed a strong association between genetic variants in the gene dense region of chromosome 20 (12) (Figure 1A). Genetic fine mapping of the region indicated 2 independent regions at this locus (the top SNPs being rs11698977 and rs6141526) (Supplemental Figure 1A; supplemental material available online with this article; https://doi.org/10.1172/JCI172963DS1). To identify the potential causal variants, genes, and cell types at this locus, we used a comprehensive annotation approach. For this, we used the following methods: human kidney gene expression and methylation quantitative trait loci (eQTL, mQTL) analysis (12, 16); human kidney single-nucleus assay for transposase accessible chromatin sequencing (snATAC-Seq) (12, 16); open chromatin peak coaccessibility (17) activity by contact (ABC) (18); Bayesian colocalization (19); and summary Mendelian randomization (SMR) (20). Six of the 8 independent methods used for GWAS prioritization highlighted *ACSS2*, *CEP250*, and *SPAG4* as likely causal genes (Figure 1B) (12, 15, 16).

We chose to focus on ACSS2, as the top fine-mapped SNP (rs11698977) was strongly associated with local cytosine methylation levels and *ACSS2* gene expression in the human kidney tubule compartment (Figure 1, C–F, Supplemental Figure 1B, and Supplemental Table 1). Bayesian colocalization analysis of the locus indicated that the eGFR GWAS identified variants, and variants that influence local methylation and the expression of *ACSS2* were shared (PPH4 >0.88, and PPAabc >0.82) (Supplemental Table 1) (12, 15, 19, 21). SMR analysis indicated that *ACSS2* expression is an effect mediator of genetic variants influencing kidney function at this locus. Lower *ACSS2* expression was associated with better kidney function (Supplemental Figure 1C and Supplemental Table 1) (20).

To further pinpoint disease-causing variants, we analyzed snATAC-Seq data from adult human kidneys and overlapped GWAS significant SNPs with human kidney single-cell open chromatin information. We prioritized 6 candidate causal SNPs in the locus that could mediate the eGFR signal and *ACSS2* expression as they were located within the open chromatin area in PT cells (Figures 1G and Supplemental Table 2). To demonstrate the causal role of these 6 prioritized eGFR variants, we applied the CRISPR/Cas9 system to delete the genomic region containing the GWAS variants in open chromatin peaks in embryonic kidney cells (Figure 1H and Supplemental Table 3). We found that genetic deletion of rs11698977, rs6120758, and rs6087649 altered *ACSS2* gene expression (Figure 1I), but not the levels of CEP250 or SPAG4, indicating that they are the likely causal variants and *ACSS2* are the likely effect mediators of this GWAS signal (Supplemental Figure 1, D and E).

In summary, we identified a genetic signal for kidney function on chromosome 20, spanning over more than 20 genes. Our complex gene prioritization strategy identified *ACSS2, CEP250*, and *SPAG4* as likely causal genes. CRISPR-based locus deletion studies highlighted genetic variants that directly regulated *ACSS2* levels, prioritizing *ACSS2* as a likely kidney disease–causing gene.

### Genetic deletion of ACSS2 protects against kidney fibrosis.

To investigate the role of ACSS2 in kidney disease, we generated mice with a genetic deletion of *Acss2* using the CRISPR/Cas9 KO system. We deleted the first exon of the *Acss2* gene. Gene and protein expression analysis confirmed the reduction of ACSS2 in the kidneys of our *Acss2*-KO mice (*Acss2^–/–^*) compared with expression levels in littermate controls (Supplemental Figure 1, F and G). *Acss2^–/–^* mice were born at the normal Mendelian ratio, without birth defects, growth abnormalities, or signs of kidney dysfunction (Supplemental Figure 1, F–J). We observed no differences in the expression of Ki67 (*mKi67*), Kim1 (*Hacvr1*), or N-gal (*Lcn2*) in the kidneys of WT or *Acss2^–/–^* mice (Supplemental Figure 1, K and L).

**Figure 2:**
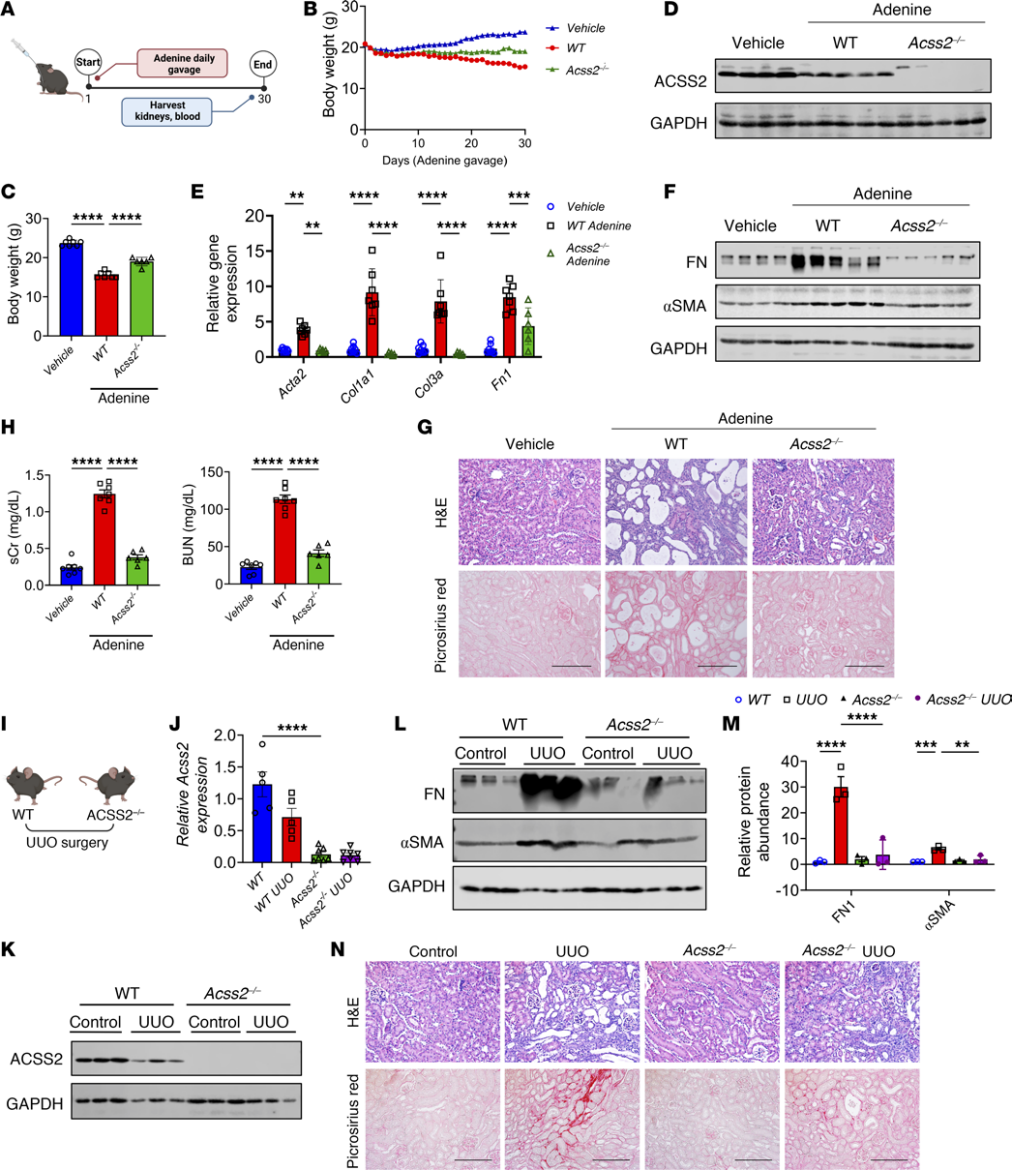
Genetic deletion of *ACSS2* protects against kidney disease. (**A**) Experimental outline. (**B**) Daily body weights of vehicle- (*n* = 7) or adenine-treated (oral gavage) WT (*n* = 7) and *Acss2^–/–^* (*n* = 6) mice. (**C**) Final body weights of WT (*n* = 7) and *Acss2^–/–^* (*n* = 6) mice gavaged with adenine or vehicle (*n* = 7). (**D**) Immunoblots of ACSS2 and GAPDH expression in kidneys of vehicle-treated (*n* = 4) or adenine-treated WT (*n* = 5) and *Acss2^–/–^* (*n* = 5) mice. (**E**) Transcript levels of αSMA (*Acta2*), *Col1a1*, *Col3a*, and *Fn1* in kidneys of vehicle-treated (*n* = 7) or adenine-treated WT (*n* = 7) and *Acss2^–/–^* (*n* = 6) mice. (**F**) Immunoblots of FN1, αSMA, and GAPDH in whole-kidney lysates of vehicle- or adenine-treated WT (*n* = 5) and *Acss2^–/–^* (*n* = 5) mice (*n* = 4). (**G**) H&E and Picrosirius red staining of kidney sections from vehicle- or adenine-treated WT and *Acss2^–/–^* mice. Scale bars: 20 μm. (**H**) sCr and BUN in vehicle- or adenine-treated WT and *Acss2^–/–^* mice. (**I**) Experimental outline. (**J**) Transcript levels of *Acss2* in kidneys of WT (*n* = 5) and *Acss2^–/–^* (*n* = 7) mice following UUO surgery. (**K**) Immunoblots of ACSS2 and GAPDH in UUO or sham-operated kidneys from WT and *Acss2^–/–^* mice. (**L**) Immunoblots showing FN1, αSMA, and GAPDH expression in kidneys of WT and *Acss2^–/–^* mice following UUO or sham surgery. (**M**) Quantification of immunoblots of FN1, and αSMA by ImageJ (NIH). (**N**) H&E- and Picrosirius red–stained images of kidneys from WT and *Acss2^–/–^* mice subjected to UUO surgery. Scale bars: 20 μm. Data are presented as the mean ± SEM. *P* values were determined by 1-way ANOVA after Tukey’s multiple-comparison test (**C**–**M**). **P < 0.01, ***P < 0.001, and ****P < 0.0001. Data in **J**–**N** are representative of multiple experiments. Protein marker was cropped from all blots but was presented in full blots file.

Genetic studies suggested a protective role for ACSS2, so we analyzed WT and *Acss2^–/–^* mice in an adenine-induced kidney disease model (Figure 2A). Body weights were lower in the adenine-treated WT mice compared with *Acss2^–/–^* and vehicle-treated mice (Figure 2, B and C). Kidney weights were increased in the WT adenine-treated group compared with vehicle-treated and *Acss2^–/–^* groups (Supplemental Figure 2A). ACSS2 gene and protein expression levels were lower in whole-kidney lysates from mice with kidney disease (Figure 2, D, J, and K, and Supplemental Figure 2, B and E–G). The change observed in whole-kidney ACSS2 levels was probably attributable to the loss of PT cells, as the single-cell data did not indicate a similar decline in ACSS2 expression in disease states (see Supplemental Figure 7, A–D). Transcript levels of fibrosis markers such as collagen type 1 α1 (*Col1a1*), collagen type 3a (*Col3a*) and fibronectin (*Fn1*) were higher in adenine-treated WT mice than in adenine-treated *Acss2^–/–^* mice (Figure 2E). Consistent with the gene expression data, protein levels of fibronectin (FN1) and α smooth muscle actin (αSMA), were higher in the WT adenine-treated group than in the adenine-treated *Acss2^–/–^* group (Figure 2F and Supplemental Figure 2C). Histological analysis indicated lower tubular injury and fibrosis in *Acss2^–/–^* mice than in WT adenine-treated mice (Figure 2G and Supplemental Figure 2D). Indicators of kidney dysfunction, such as serum creatinine (sCr) and blood urea nitrogen (BUN), were elevated in WT adenine-treated mice compared with adenine-treated *Acss2^–/–^* mice (Figure 2H).

Next, we validated our findings in 2 different models of established kidney disease induced by unilateral ureteral obstruction (UUO) or folic acid injection (FAN) (Figure 2I) (22, 23). Protein markers of fibrosis, including levels of FN1 and αSMA, were higher in the UUO and FAN kidney fibrosis models, but their levels were observably lower in the kidneys of *Acss2^–/–^* mice with UUO or FAN injury (Figure 2, L and M, and Supplemental Figure 2, H and I). Transcript levels of *Col1a1*, *Col3a*, and *Fn1* were higher in the kidney disease models, but they were lower in *Acss2^–/–^* mice with kidney injury (Supplemental Figure 2, J and K). Histological changes such as tubule atrophy and interstitial fibrosis were also lower in *Acss2^–/–^* mice (Figure 2N and Supplemental Figure 2, L–N). Clinical markers of kidney injury, sCr and BUN, were both increased in WT mice injected with folic acid but not in *Acss2^–/–^* mice (Supplemental Figure 2, O and P).

We next established an in vitro cell model of fibrosis by culturing primary kidney tubular epithelial cells (TECs) from WT and *Acss2^–/–^* mice in the presence of TGF-β1. TECs from WT mice showed higher expression of fibrosis markers, including *Col1a1*, *Col3a*, *Fn1*, and *Acta2*, when treated with TGF-β1, whereas cells lacking ACSS2 showed lower levels of TGF-β1–induced fibrosis gene expression (Supplemental Figure 2Q). Consistently, immunoblotting performed with the lysates of primary TECs treated with TGF-β1 showed increased protein levels of αSMA and FN1, which were lower in *Acss2^–/–^* cells (Supplemental Figure 2R and Supplemental Figure 3A).

In summary, *Acss2* -KO mice and kidney tubule cells showed protection from kidney disease.

### ACSS2 expression correlates with DNL and kidney fibrosis.

**Figure 3:**
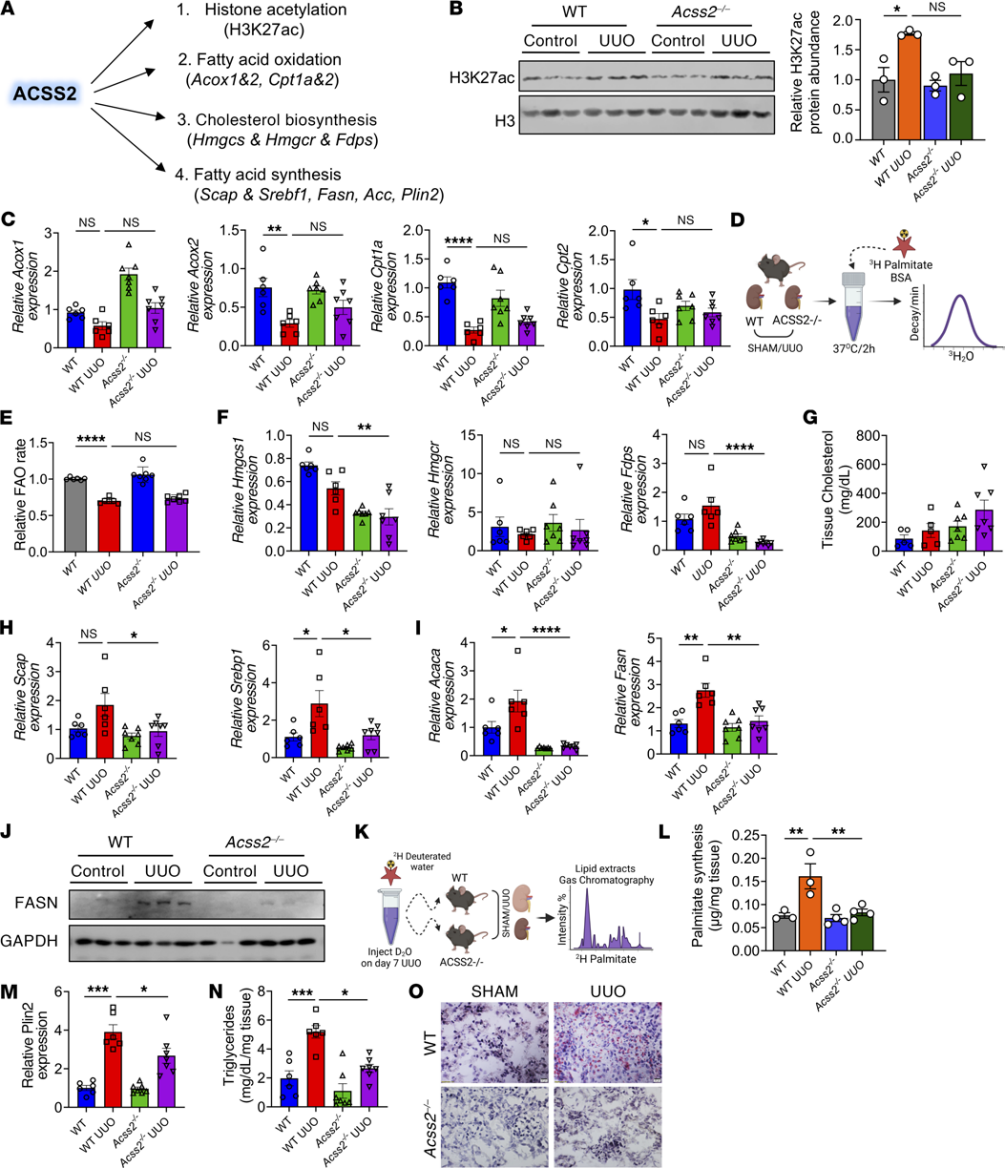
Kidney ACSS2 expression correlates with changes in genes in DNL. (**A**) Biochemical functions of ACSS2. (**B**) Immunoblots of H3K27ac and H3 protein levels in total histones extracted from kidneys of WT and *Acss2^–/–^* mice subjected to UUO or sham surgery (left). Quantification of H3K27ac levels by ImageJ (right). (**C**) Transcript levels of *Acox1*, *Acox2*, *Cpt1a*, and *Cpt2* in kidneys of WT (*n* = 6) and *Acss2^–/–^* (*n* = 7) mice with and without UUO. (**D**) FAO experimental scheme. (**E**) FAO rate in WT (*n* = 6) and *Acss2^–/–^* (*n* = 7) mice with and without UUO surgery. (**F**) Transcript levels of *Hmgcs1*, *Hmgcr*, and *Fdps* in kidneys of WT (*n* = 6) and *Acss2^–/–^* (*n* = 7) mice with and without UUO surgery. (**G**) Total cholesterol in whole kidneys of WT (*n* = 5) and *Acss2^–/–^* (*n* = 7) mice following UUO. (**H**) Transcript levels of *Scap* and *Srebp1* in kidneys of WT (*n* = 6) and *Acss2^–/–^* (*n* = 7) mice following UUO. (**I**) Transcript levels of *Fasn* and *Acaca* in kidneys of WT (*n* = 6) and *Acss2^–/–^* (*n* = 7) mice following UUO. (**J**) Immunoblots of FASN and GAPDH expression in whole kidneys of WT and *Acss2^–/–^* mice with UUO. (**K**) Measurement of DNL. (**L**) DNL rate in WT (*n* = 3) and *Acss2^–/–^* (*n* = 4) mice with and without UUO. (**M**) Transcript levels of *Plin2* in kidneys of WT (*n* = 6) and *Acss2^–/–^* (*n* = 7) mice with UUO. (**N**) Kidney TG levels in WT (*n* = 6) and *Acss2^–/–^* (*n* = 7) mice with UUO. (**O**) Oil Red O staining in kidneys of WT and *Acss2^–/–^* mice with UUO. Scale bars: 10 μm. Data are presented as the mean ± SEM. *P < 0.05, **P < 0.01, ***P < 0.001, and ****P < 0.0001, by 1-way ANOVA after Tukey’s multiple-comparison test (**B**–**N**). The protein marker was cropped from all blots but is presented in the full blots file.

ACSS2 is a multifunctional enzyme involved in the generation of acetyl coenzyme A (ac-CoA) from intracellular or nuclear acetate pools and plays a role in a variety of biochemical processes (24, 25) (Figure 3A). Intranuclear ac-CoA is generated by ACSS2 and can be used for histone posttranslational modification (PTM). The role of ACSS2 in histone 3 lysine 27 acetylation (H3K27ac) has been previously described (26). Therefore, we extracted total histones from kidneys of WT and *Acss2^–/–^* mice at baseline and following UUO injury and compared H3K27ac levels by Western blotting. We found no observable changes in histone acetylation (H3K27ac) in *Acss2^–/–^* mice at baseline or following UUO injury (Figure 3B). FAO genes such as acyl CoA oxidase 1 (*Acox1*), *Acox2*, carnitine palmitoyl transferase 1 (*Cpt1a*), and *Cpt2* were measured in kidneys of WT and *Acss2^–/–^* mice following UUO injury. We observed lower expression of *Acox1*, *Acox2*, *Cpt1a*, and *Cpt2* in WT UUO kidneys (Figure 3C). The reduction of *Acox1*, *Acox2*, *Cpt1a*, and *Cpt2* was not rescued in *Acss2^–/–^* kidneys (Figure 3C). Finally, we tested FAO rates in the kidneys of WT and *Acss2^–/–^* mice using tritium-labeled palmitic acid (^3^H-palmitate) (Figure 3D). We observed comparable FAO rates in the kidneys of WT and *Acss2^–/–^* mice at baseline (Figure 3E). Injured kidneys showed lower FAO rates which were not rescued by *Acss2* gene deletion (Figure 3E). Furthermore, we tested FAO in primary tubular cells by a Seahorse-based palmitic acid oxidation. The Seahorse-based FAO analysis showed no significant difference in the oxygen consumption rate (OCR) or ATP levels between WT and *Acss2^–/–^* primary tubule cells following palmitic acid supplementation (Supplemental Figure 3, B–D). Consistently, in our cultured tubule cell system, we found that expression of *Acox1*, *Acox2*, *Cpt1a*, and *Ppara* was lower following TGF-β1 treatment, consistent with prior studies (9) and indicating a defect in FAO in kidney fibrosis. However, this defect was not rescued in *Acss2^–/–^* cells (Supplemental Figure 3E).

ACSS2-generated ac-CoA also fuels cholesterol biosynthesis. Hydroxy methyl glutaryl CoA synthase (HMGCS1) and HMGC reductase (HMGCR) are the major enzymes involved in the synthesis of the precursor mevalonate for cholesterol synthesis (Figure 3A). We measured the expression of *Hmgcs1*, *Hmgcr*, and farnesyl diphosphate synthase (*Fdps*) in the kidneys of WT and *Acss2^–/–^* mice with sham or UUO surgery. We did not observe a change in the expression of genes involved in cholesterol biosynthesis (Figure 3F), which was consistent with the total kidney cholesterol levels measured in WT and *Acss2^–/–^* mice (Figure 3G).

Finally, we examined DNL, since ac-CoA is utilized in the synthesis of fatty acids. ac-CoA carboxylase (ACACA) and FASN are the key enzymes in fatty acid synthesis, with sterol regulatory binding protein 1 (SREBP1) and SREBP cleavage protein (SCAP) being their key upstream regulators (27). We found that the expression of *Scap*, *Srebp1*, *Fasn*, and *Acaca* was higher in the UUO model of kidney injury (Figure 3, H and I). Loss of ACSS2 was associated with lower expression of *Scap*, *Srebp1*, *Fasn*, and *Acaca* compared with expression in the kidneys of WT UUO mice (Figure 3, H and I). Protein levels of FASN were higher in WT UUO mice and were reduced in *Acss2^–/–^* mice with kidney injury (Figure 3J and Supplemental Figure 3F).

Next, we assessed the DNL rate in vivo by quantifying the incorporation of deuterated water (D_2_O) into palmitate in the kidney (Figure 3K). We observed a higher DNL rate in WT UUO kidneys compared with *Acss2^–/–^* UUO kidneys (Figure 3L). Consistently, tissue triglyceride (TG) levels and the expression of perilipin 2 (*Plin2*), a marker of TG accumulation (28), were higher in UUO kidneys but lower in the UUO injury model of *Acss2^–/–^* mice (Figure 3, M–O). Primary TECs treated with TGF-β1 increased *Fasn* and *Plin2* gene expression, which was consistent with higher TG levels, confirming that TGF-β1 can induce fatty acid synthesis (Supplemental Figure 3, G, I–O, P, and Q).

In summary, we observed changes associated with DNL in the mouse kidney disease model that were improved in the absence of ACSS2.

### Pharmacological inhibition of fatty acid synthesis protects against kidney fibrosis.

Since our cell studies suggested a key role of ACSS2 in kidney DNL, we next examined whether direct inhibition of FASN could protect against kidney fibrosis. First, we tested 2 widely used drugs, FASNall and TVB-3664, in a cell culture model of fibrosis (Supplemental Figure 3G) (29, 30). We found that treatment with FASNall or TVB-3664 did not induce cell death in primary TECs (Supplemental Figure 3H). Treatment of tubule cells with FASNall prevented TGF-β1–induced increase in *Fasn* and *Plin2* expression and TG accumulation (Supplemental Figure 3, I–M). FASNall treatment protected against changes in the expression of TGF-β1–induced profibrotic genes, including *Col1a1*, *Col3a*, *Fn1*, and *Acta2* (Supplemental Figure 3N). We observed a similar effect with TVB-3664, a more potent FASN inhibitor, on profibrotic gene expression changes, *Plin2* expression, TG accumulation, and total cell TG levels (Supplemental Figure 3, M and O–R).

We verified our pharmacological studies by genetic knockdown of *Fasn* using an siRNA (si*Fasn*) in mouse primary TECs (Supplemental Figure 4A). Gene expression analysis of *Fasn* indicated successful knockdown (~60%) (Supplemental Figure 4B). si*Fasn* substantially protected not only against TGF-β1–induced *Plin2*, but also against increases in the expression of *Acta2*, *Col1a1*, *Col3a*, and *Fn1* (Supplemental Figure 4, A and B). We observed similar results in *Scap*-deficient (*Scap^fl/fl^ AdCre*) tubule cells (Supplemental Figure 4, C and D). *Scap*-deficient kidney tubule cells had lower expression of *Fasn* and *Plin2*, confirming the dependency of DNL gene expression on the SCAP/SREBP1 axis (Supplemental Figure 4C).

Next, we tested the effect of FASN inhibition in the UUO mouse model of kidney fibrosis (Supplemental Figure 4E). The expression of *Fasn* and *Plin2* was higher in UUO kidneys (Supplemental Figure 4F). FASNall ameliorated the rise in *Fasn* and *Plin2* expression (Supplemental Figure 4F). Similarly, TG levels were higher in UUO kidneys, but lower in animals treated with FASNall (Supplemental Figure 4G). Oil Red O staining showed lipid accumulation in the UUO kidneys, which was lower in FASNall-injected animals (Supplemental Figure 4H). Protein levels of FN1 and αSMA were lower in FASNall-treated UUO mice compared with sham-treated animals (Supplemental Figure 4, I and J). Histological analysis indicated severe tubule atrophy and fibrosis in vehicle-treated UUO kidneys and less atrophy and fibrosis in FASNall-treated UUO mice (Supplemental Figure 4K). Picrosirius red staining indicated less tissue scarring in FASNall-treated UUO mouse kidneys compared with vehicle-treated UUO kidneys (Supplemental Figure 4K). Finally, expression of the fibrosis markers *Acta2*, *Col1a1, Col3a,* and *Fn1* was lower in FASNall-treated UUO mouse kidneys (Supplemental Figure 4L), suggesting that inhibition of FASN by FASNall prevented kidney injury and fibrosis development.

### Genetic deletion of FASN protects against kidney disease development.

**Figure 4:**
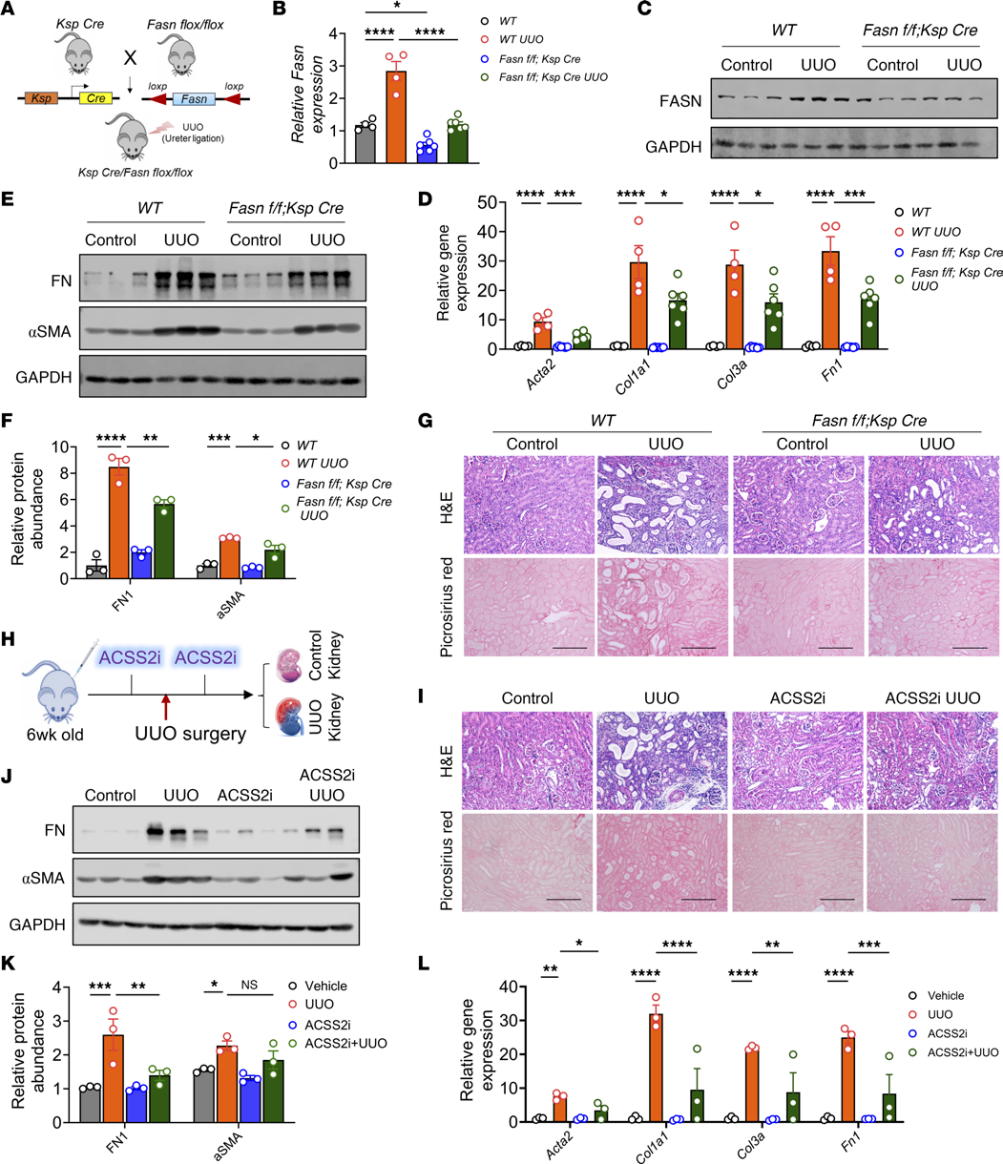
Tubule cell–specific deletion of *FASN* or pharmacological inhibition of ACSS2 protects against kidney disease. (**A**) Experimental design. (**B**) Transcript levels of *Fasn* (*n* = 4, WT; *n* = 6, *Fasn^fl/fl^ Ksp Cre*). (**C**) Immunoblots of FASN and GAPDH levels in *Fasn^fl/fl^ Ksp Cre* and WT mice. (**D**) Transcript levels of *Acta2*, *Col1a1*, *Col3a*, and *Fn1* in kidneys of *Fasn^fl/fl^ Ksp Cre* (*n* = 6) and WT (*n* = 4) mice. (**E**) Immunoblots showing FN1, αSMA, and GAPDH levels in kidneys of *Fasn^fl/fl^ Ksp Cre* and WT mice following UUO. (**F**) Quantification of FN1 and αSMA immunoblots by ImageJ. (**G**) H&E and Picrosirius red staining of UUO kidneys from *Fasn^fl/fl^ Ksp Cre* and WT mice (left). Fibrosis was quantified using ImageJ (right). Scale bars: 20 μm. (**H**) ACSS2i experimental design. (**I**) H&E and Picrosirius red staining of UUO kidneys from mice injected or not with ACSS2i. Scale bars: 20 μm. (**J**) Immunoblots showing FN1, αSMA, and GAPDH levels in kidneys of mice injected with ACSS2i that are subjected to UUO. (**K**) Quantification of FN1 and αSMA immunoblots by ImageJ. (**L**) Transcript levels of *Acta2*, *Col1a1*, *Col3a*, and *Fn1* in UUO kidneys of mice injected with ACSS2i (*n* = 3) or PBS (*n* = 3). Data are presented as the mean ± SEM. *P < 0.05, **P < 0.01, ***P < 0.001, and ****P < 0.0001, by 1-way ANOVA after Tukey’s multiple-comparison test (**B**, **D**, **F**, **K**, and **L**). Data in **B**–**G** are representative of multiple experiments. The protein marker was cropped from all blots but is presented in the full blots file.

Since FASN is the key enzyme in DNL, we next tested whether tubule-specific genetic deletion of *Fasn* would protect mice from developing kidney disease. We generated tubule-specific *Fasn*-KO mice by crossing *Ksp Cre* mice with *Fasn*-floxed mice (Figure 4A). While whole-body KO of *Fasn* is embryonically lethal, tubule-specific *Fasn*-KO (*Fasn^fl/fl^ Ksp Cre*) mice were grossly indistinguishable from their WT littermates. Gene expression analysis revealed that *Fasn* expression was lower in *Fasn^fl/fl^ Ksp Cre* mice than in WT mice (Figure 4B). We tested the role of FASN in adenine-induced kidney disease and the UUO models. Transcript and protein levels of FASN were increased in diseased kidneys of WT mice, but their levels were observably lower in mice with tubule-specific *Fasn* deletion (Figure 4, B and C, and Supplemental Figure 4, M and N). Body weight was lower in adenine-treated WT mice when compared with controls, but it was less reduced in adenine-treated *Fasn^fl/fl^ Ksp* *Cre* mice (Supplemental Figure 4, O and P). Gene and protein expression analysis of *Acta2*, *Col1a1*, *Col3a*, and *Fn1* indicated that tubule-specific deletion of *Fasn* lessened kidney fibrosis (Figure 4, D–F, and Supplemental Figure 4, Q–S). H&E and Picrosirius red staining analyses revealed tubular atrophy and collagen deposition in WT adenine-treated mice and UUO-operated mice, whereas *Fasn^fl/fl^* *Ksp Cre* mice had less tubule damage and fibrosis (Figure 4G and Supplemental Figure 4, T and U). Kidney function tests such as those measuring sCr and BUN levels were also improved in adenine-treated *Fasn*-KO mice compared with WT adenine-treated mice (Supplemental Figure 4V). Together, these results indicate that deletion of FASN in tubule cells ameliorated kidney dysfunction, indicating a key role of tubule-specific DNL in kidney dysfunction.

### Pharmacological inhibition of ACSS2 prevents kidney fibrosis.

Next, we tested whether pharmacological inhibition of ACSS2 would also reduce kidney fibrosis (31). We injected mice with ACSS2i [*N*-(2,3-di-2-thienyl-6-quinoxalinyl)-*N*′-(2-methoxyethyl) urea] prior to UUO surgery and on the fourth day after the UUO surgery (Figure 4H). Histological analysis by H&E staining and fibrosis quantification by Picrosirius red staining indicated lower fibrosis and tubule atrophy in ACSS2i-treated mice (Figure 4I and Supplemental Figure 4W). Gene and protein expression analysis of fibrosis markers (*Acta2*, *Col1a1*, *Col3a*, and *Fn1*) further confirmed the protective effect of ACSS2i in kidney fibrosis (Figure 4, J–L). Importantly, ACSS2i injection also inhibited lipid accumulation, which was evidenced by lower expression levels of *Fasn*, *Plin2*, and TG accumulation in UUO kidneys (Supplemental Figure 4, X–Z).

### Reduced NADPH consumption and a lower oxidative state in the absence of ACSS2.

**Figure 5:**
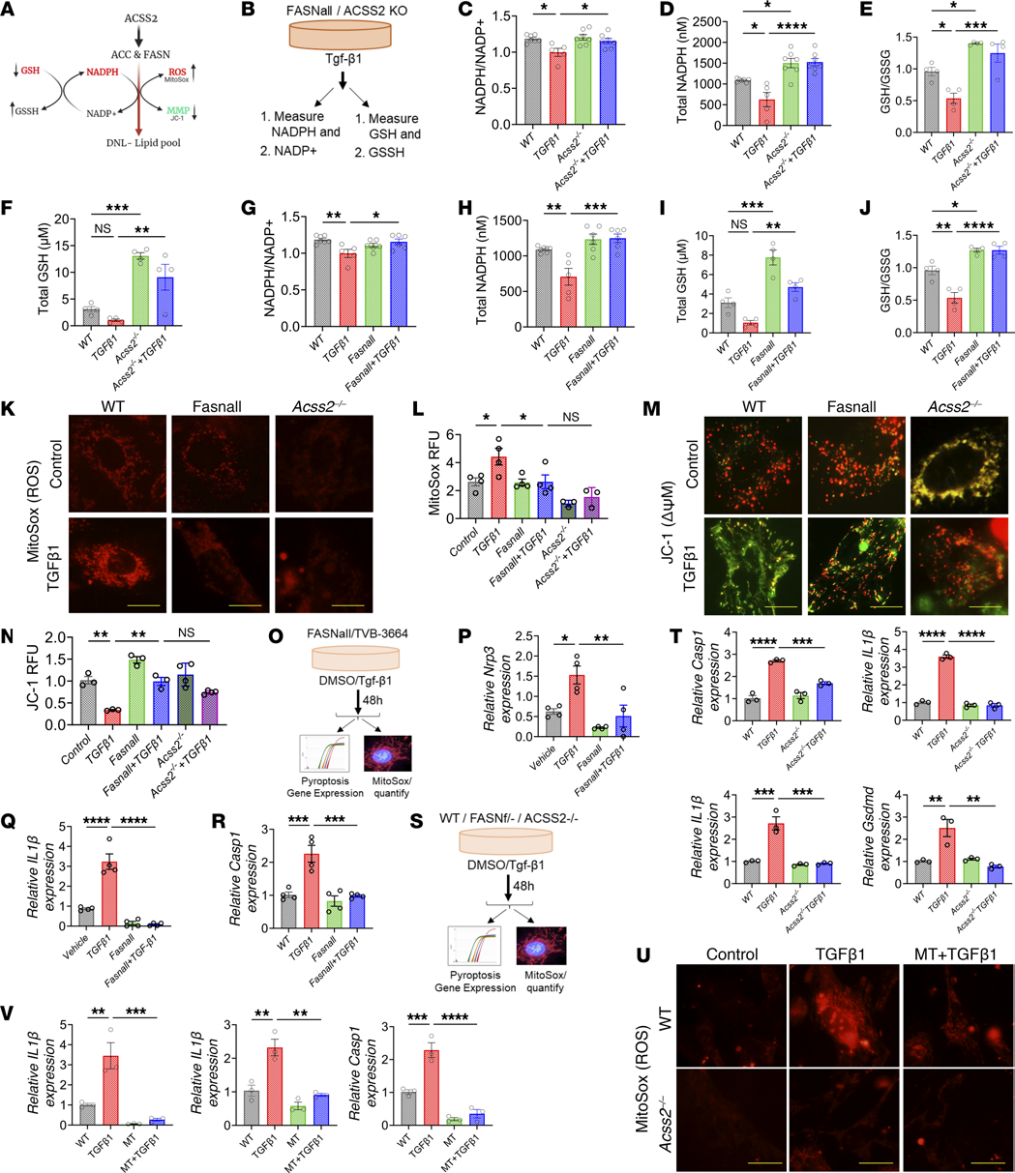
DNL in kidney tubules is associated with higher mtROS. (**A**) Fatty acid synthesis requires a large amount of NADPH, leading to elevated ROS levels in the kidneys. MMP, mitochondria membrane potential. (**B**) Experimental hypothesis. (**C**) NADPH^+^/NDP^+^ ratio in tubule cells treated with TGF-β1. (**D**) Total NADPH levels in *Acss2^–/–^* cells treated with TGF-β1. (**E**) GSH/GSSG ratio in *Acss2^–/–^* cells treated with TGF-β1. (**F**) Total GSH levels in *Acss2^–/–^* cells treated with TGF-β1. (**G**) NADPH^+^/NDP^+^ ratio in cells treated with TGF-β1 and FASNall. (**H**) Total NADPH levels in cells treated with TGF-β1 and FASNall. (**I**) Total GSH levels in cells treated with TGF-β1 and FASNall. (**J**) GSH/GSSG ratio in cells treated with TGF-β1 and FASNall. (**K**) MitoSox staining in WT, *Acss2^–/–^*, and FASNall-cells treated with TGF-β1. Scale bars: 10 μm. (**L**) Quantification of MitoSox fluorescence. (**M**) JC-1 staining of WT, *Acss2^–/–^,* and FASNall cells treated with TGF-β1. Scale bars: 10 μm. (**N**) Quantification of JC-1 (red/green ratio) fluorescence. RFU, relative fluorescence units. (**O**) Experimental scheme. (**P**) Transcript levels of *Nlrp3* in tubule cells treated with FASNall and TGF-β1. (**Q**) Transcript levels of *IL1B* in tubule cells treated with FASNall and/or TGF-β1. (**R**) Transcript levels of *Casp1* in tubule cells treated with FASNall and/or TGF-β1. (**S**) Experimental scheme. (**T**) Transcript levels of *Casp1*, *IL1B*, *IL18*, and *Gsdmd* in *Acss2^–/–^* tubule cells treated with TGF-β1. (**U**) MitoSox staining and (**V**) transcript levels of *IL1B*, *IL18*, and *Casp1* in *Acss2^–/–^* tubular cells treated with TGF-β1 and/or MT. Scale bars: 10 μm. Data are presented as the mean ± SEM. Each experiment was repeated at least twice, and the data presented in this figure are representative of multiple experiments. *P < 0.05, **P < 0.01, ***P < 0.001, and ****P < 0.0001, by 1-way ANOVA after Tukey’s multiple-comparison test (**C**–**V**).

Next, we aimed to understand the mechanism of how ACSS2 deletion and DNL inhibition afforded protection from kidney fibrosis. Our prior experiments with tubule-specific CD36-transgenic mice indicated that TG accumulation alone in kidney tubules per se was not sufficient to cause full-spectrum of fibrosis (9). Therefore, we hypothesized that DNL might be associated with increased NADPH utilization, leaving cells at higher risk of oxidative damage (32) (Figure 5A). We measured the NADPH^+^/NADP^+^ ratio in WT and *Acss2^–/–^* cells using a luminescence probe (Figure 5B). TGF-β1 treatment lowered the NADPH^+^/NADP^+^ ratio in WT cells but not in *Acss2^–/–^* cells (Figure 5C). Total NADPH levels were higher in *Acss2^–/–^* cells than in WT cells (Figure 5D). Levels of intracellular oxidized (GSSG) to reduced glutathione (GSH) is an important measure of cellular redox state. GSH measurements revealed a lower GSH/GSSG ratio in TGF-β1–treated WT cells but not in *Acss2^–/–^* cells (Figure 5E). Total GSH levels were higher in *Acss2^–/–^* cells than in WT cells (Figure 5F).

We then tested the role of FASN and DNL in regulating NADPH^+^/NADP^+^ ratios. We treated cells with TGF-β1 in the presence or absence of FASNall and quantified NADPH and GSH levels. The NADPH^+^/NADP^+^ ratio was lower in TGF-β1–treated tubule cells, but it was preserved in FASNall-treated tubule cells (Figure 5, G, and H). GSH levels and the relative GSH/GSSG ratio were lower in TGF-β1–treated cells but preserved by the FASN inhibitor (Figure 5I), indicating that FASN uses a substantial amount of NADPH for fatty acid synthesis, which scavenges NADPH, leaving more oxidized GSSG (Figure 5J).

Next, we measured mitochondrial ROS using MitoSox. We observed increased ROS levels in TGF-β1–treated kidney tubule cells (Figure 5, K and L). Tubule cells with genetic loss of *Acss2* had lower ROS levels compared with WT cells following TGF-β1 treatment (Figure 5, K and L). We observed similar results following FASNall treatment, indicating the role of fatty acid synthesis in modulating cellular ROS levels (Figure 5, K and L). We then examined mitochondrial parameters (33). We observed a less negative mitochondrial membrane potential in TGF-β1–treated kidney tubule cells, as measured by monomeric JC-1 accumulation (Figure 5, M and N). *Acss2^–/–^* cells or treatment of WT cells with FASNall attenuated this effect (Figure 5, M and N).

Damaged mitochondria can stimulate mitophagy (34). We monitored mitophagy using MitoQ, a plasmid that expresses the mCherry-eGFP fusion construct under the COX8 promoter, which is selective to the mitochondria (35). This plasmid labels mitochondria in yellow when cells are in a fed state or treated with bafilomycin A (BA), whereas enhanced mitophagy induced by starvation or carbonyl cyanide m-chlorophenyl hydrazone (CCCP) turns the plasmid red as a result of the low lysosomal pH, which quenches GFP fluorescence. We found that nutrient-deprived “starved” cells or CCCP treatment had increased mitophagy flux (a higher number of red mitochondria) and that loss of ACSS2 further enhanced the number of mitolysosomes (Supplemental Figure 5, A and B). We confirmed the enhanced mitophagy flux in *Acss2^–/–^* cells compared with WT cells when we analyzed mitophagy flux by LC3 and PARKIN1 immunoblotting in cells subjected to starvation or BA (Supplemental Figure 5, C and D).

In summary, we observed an increased oxidative state in profibrotic tubule cells, inhibition of DNL or ACSS2 protected against ROS accumulation and mitochondrial defects.

### Mitochondrial injury induces pyroptosis and inflammation.

To further understand the mechanism of ACSS2- and FASN-mediated kidney fibrosis development, we next examined whether mitochondrial ROS (mtROS) and mitochondrial defect–mediated activation of the NLRP3 inflammasome pathway play a role in the process (Figure 5O) (36, 37). We observed increased inflammasome activation in TGF-β1–treated cultured kidney tubule cells. Expression of *Nlrp3*, *IL1B*, and caspase 1 (*Casp1*) was higher in TGF-β1–treated cells (Figure 5, P and Q). Inflammasome activation was dependent on DNL, as the levels of *Nlrp3*, *IL1B*, and *Casp1* were lower in TGF-β1–treated tubule cells following treatment with the FASN inhibitor FASNall or TVB-3664 (Figure 5, P–R, and Supplemental Figure 5E). We observed similar results in *Scap*-KO cells or in cells with heterozygous loss of *Fasn* (Figure 5S, and Supplemental Figure 5, F–H). Furthermore, tubule cells obtained from *Acss2^–/–^* mice also showed protection from TGF-β1–induced NLRP3 inflammasome activation (Figure 5T).

To determine whether changes in pyroptosis and fibrosis are dependent on TGF-β1–induced elevated mtROS, we tested the effect of MitoTempo, a mitochondria-specific ROS scavenger (38). We found that the increase in inflammasome activation genes, including *IL1B*, *IL18*, and *Casp1*, following TGF-β1 treatment was efficiently lowered by MT (Figure 5, U and V). Consistently, MT treatment or *Fasn* genetic deletion lowered TGF-β1–induced expression of fibrosis markers, indicating a role of ROS in fibrosis (Supplemental Figure 5, I–K). In summary, increased ACSS2-mediated DNL by FASN appeared to lower NADPH and GSH, leading to elevated ROS levels and enhanced NLRP3-dependent pyroptosis in tubule cells.

### Deletion of Acss2 or DNL attenuates pyroptosis-induced inflammatory fibrosis in mice.

**Figure 6:**
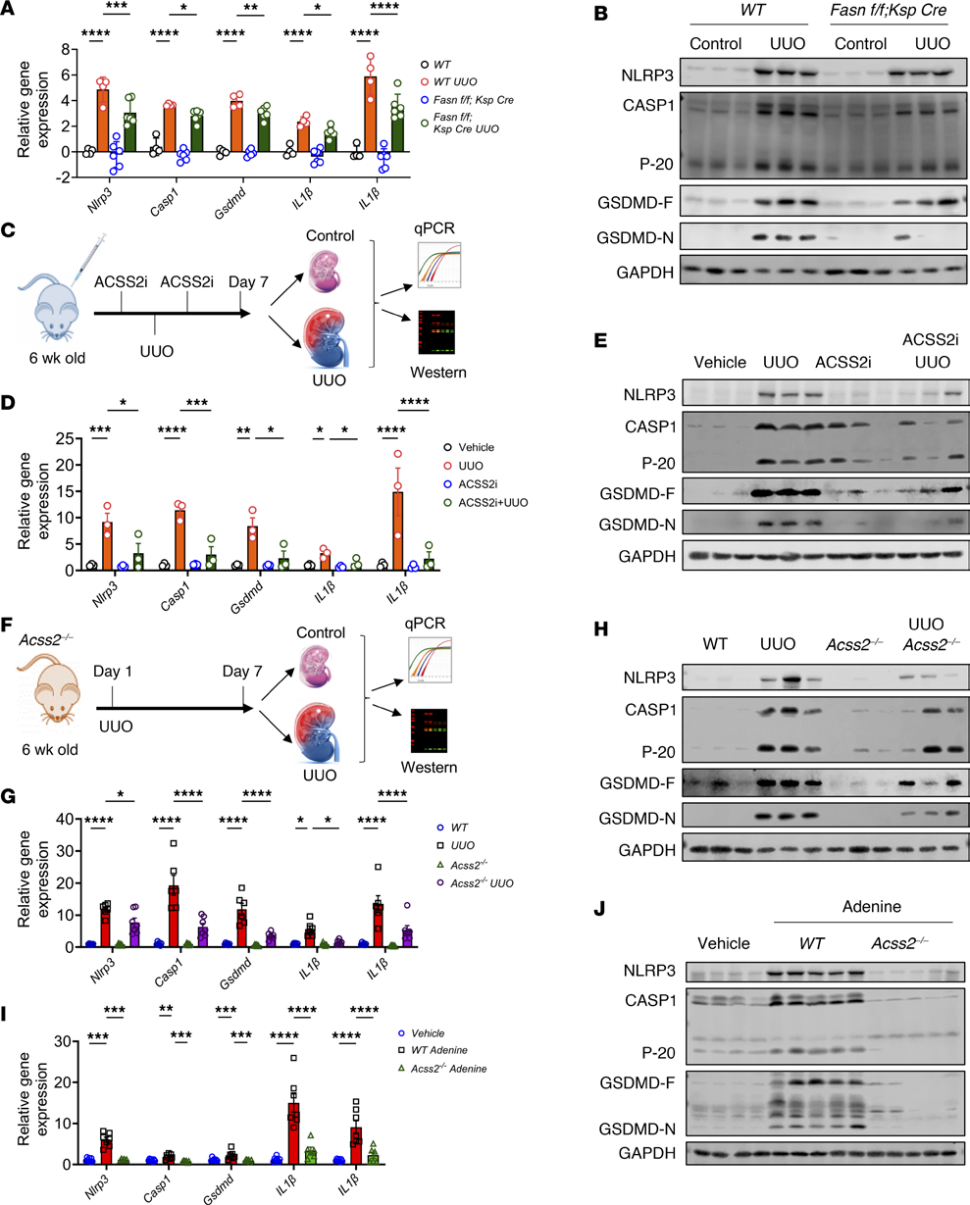
Genetic deletion or pharmacological inhibition of FASN or ACSS2 attenuates inflammasome activation. (**A**) Transcript levels of *Nlrp3*, *IL1B*, *IL18*, and *Gsdmd* in control and UUO kidneys of *Fasn^fl/fl^ Ksp Cre* (*n* = 6) and WT (*n* = 4) mice. (**B**) Immunoblots of NLRP3, CASP1, P20 (cleaved caspase 1), GSDMD-F, GSDMD-N, and GAPDH expression in UUO kidneys from WT and *Fasn^fl/fl^ Ksp Cre* mice. (**C**) Experimental design. (**D**) Transcript levels of *Nlrp3*, *IL1B*, *IL18*, and *Gsdmd* in UUO kidneys of mice injected with ACSS2i (*n* = 3) or vehicle (*n* = 3). (**E**) Immunoblots showing NLRP3, CASP1, P20, GSDMD-F, GSDMD-N, and GAPDH expression in UUO kidneys from mice injected with vehicle (*n* = 3) or ACSS2i (*n* = 3). (**F**) Experimental design. (**G**) Transcript levels of *Nlrp3*, *IL1B*, *IL18*, and *Gsdmd* in WT (*n* = 6) and *Acss2^–/–^* (*n* = *7*) mice following UUO surgery. (**H**) Immunoblots showing NLRP3, CASP1, P20, GSDMD-F, GSDMD-N, and GAPDH expression in UUO kidneys from WT and *Acss2^–/–^* mice. (**I**) Transcript levels of *Nlrp3*, *IL1B*, *IL18*, and *Gsdmd* in adenine-treated CKD kidneys from WT (*n* = 7) and *Acss2^–/–^* (*n* = *6*) mice. (**J**) Immunoblots showing NLRP3, CASP1, P20, GSDMD-F, GSDMD-N, and GAPDH expression in adenine-treated CKD kidneys from WT and *Acss2^–/–^* mice. Data are presented as the mean ± SEM. *P < 0.05, **P < 0.01, ***P < 0.001, and ****P < 0.0001, by 1-way ANOVA after Tukey’s multiple-comparison test (**A**, **D**, **G**, and **I**). Data in **A** were log_2_ transformed. The protein marker was cropped from all blots but is presented in the full blots file.

To gain further insight into the role of ACSS2 and DNL in the development of kidney fibrosis, we investigated whether the activation of NLRP3 inflammasome–mediated mtROS and mitochondrial defects contribute to this process. We analyzed *Acss2^–/–^* mice, *Fasn^fl/fl^ Ksp Cre* mice, and mice treated with ACSS2 or FASN inhibitors. Gene expression analysis of the inflammasome pathway revealed higher levels of *Nlrp3*, gasdermin D (*Gsdmd*), *IL1B*, *IL18*, and *Casp1* in the UUO model of kidney fibrosis (Figure 6, A, D, F, and G and Supplemental Figure 6A). We found that gene expression of *Nlrp3*, *IL1B*, *IL18*, *Casp1*, and *Gsdmd* was lower in the kidneys of *Fasn^fl/fl^ Ksp Cre* mice (Figure 6A), FASNall-treated mice (Supplemental Figure 6A), ACSS2i-treated mice (Figure 6, C and D), and *Acss2^–/–^* mice with UUO (Figure 6, F and G). *Gsdmd* mRNA was detected in PT cells (LDL receptor 2 [*Lrp2^+^*]) particularly, and its expression was elevated in injured kidneys (Supplemental Figure 6, B and C). Immunoblotting for NLRP3, caspase 1 (CASP1), and GSDMD showed higher levels in UUO kidneys, whereas their expression was lower in the kidneys of *Fasn^fl/fl^ Ksp Cre* mice with UUO injury (Figure 6B, and Supplemental Figure 6D). The cleaved form of GSDMD (N-terminal GSDMD [GSDMD-N]) is the effector molecule of pyroptosis (37). The level of GSDMD-N was higher in the UUO kidneys of WT mice and lower in the UUO kidneys of *Fasn ^fl/fl^ Ksp Cre* mice (Figure 6B and Supplemental Figure 6D). Protein levels of NLRP3, CASP1, cleaved CASP1 (P20), full-length GSDMD (GSDMD-F), and GSDMD-N in the kidneys of mice treated with ACSS2i (Figure 6E and Supplemental Figure 6E) or FASNall (Supplemental Figure 6, E, and G) treatment or *Acss2^–/–^* (Figure 6H and Supplemental Figure 6H) kidneys following UUO surgery were lower compared with levels in the vehicle-treated UUO kidneys. We observed similar results in the adenine-induced kidney disease models with genetic deletion of *Acss2* or *Fasn*, suggesting that NLRP3 inflammasome activation is associated with kidney disease development in these models (Figure 6, I and J, and Supplemental Figure 6, I–K).

### The DNL gene signature in human kidney disease samples is highly enriched in PT cells.

**Figure 7:**
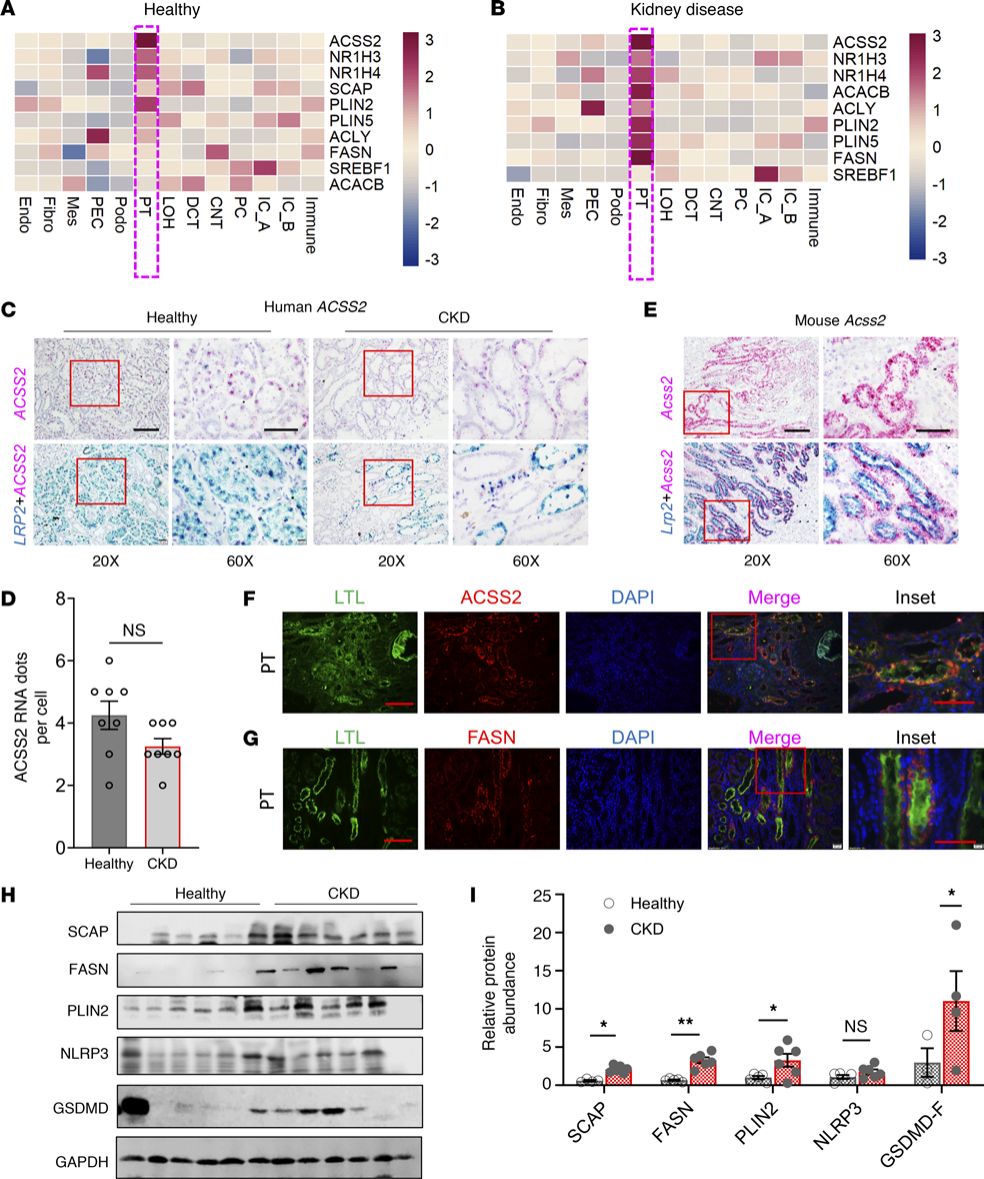
Changes in DNL gene expression in the kidneys of patients with CKD. (**A**) Relative gene expression *z* scores for *ACSS2*, *NR1H3*, *NR1H4*, *SREBF1*, *SCAP*, *PLIN2*, *PLIN5*, *ACACAB*, *ACLY*, and *FASN* in healthy human kidney snRNA-Seq data. PEC, parietal epithelial cells; Mes, mesangial cells; IC_A, intercalated cells A; IC_B, intercalated cells B. (**B**) Gene expression *z* score for *ACSS2*, *NR1H3*, *NR1H4*, *SREBF1*, *SCAP*, *PLIN2*, *PLIN5*, *ACACAB*, *ACLY*, and *FASN* from human CKD kidney snRNA-Seq in various kidney cell types. (**C**) ISH of human *ACSS2* and *LRP2* in healthy and CKD human kidneys. Original magnification, ×20 (scale bar: 20 μm) and ×60 (scale bar: 10 μm). (**D**) Quantification of RNA ISH (*n* = 4). (**E**) ISH of mouse *Acss2* and *Lrp2* in healthy mouse kidney samples. Original magnification, ×20 (scale bar: 20 μm) and ×60 (scale bar: 10 μm). (**F**) Immunofluorescence images of ACSS2 expression in healthy human kidneys. LTL identifies the PT segment. Scale bars: 20 μm and 10 μm (inset). (**G**) Immunofluorescence images of FASN expression in healthy human kidneys. LTL identifies the PT segment. Scale bars: 20 μm and 10 μm (inset). (**H**) Immunoblots showing SCAP, FASN, PLIN2, NLRP3, and GSDMD expression in healthy and CKD kidneys (*n* = 6). (**I**) Quantification of immunoblots. Data were normalized to GAPDH and are presented as the mean ± SEM. In the healthy group, the sixth sample was excluded from statistical analysis because of its disease-like characteristics. The first sample from the healthy group was excluded from the SCAP analysis. Only 3 healthy samples and 4 CKD samples were included in the GSMD statistical analysis due to high variability. *P < 0.05 and **P < 0.01, by 1-way ANOVA after Tukey’s multiple-comparison test (**D** and **I)**. The protein marker was cropped from all blots but is presented in the full blots file.

To understand the relevance of ACSS2 and DNL in patients with kidney disease, we first analyzed the fatty acid synthesis gene signature in human kidney single-cell expression data (http://www.susztaklab.com/hk_genemap/scRNA). Healthy controls and kidney disease samples showed strong expression of DNL genes, including *ACSS2*, *NR1H3* (liver X receptor α [*LXRA*]), *NR1H4* (farnesoid X receptor [*FXR*]), *SREBF1*, *SCAP*, *PLIN2*, *PLIN5*, *ACACAB*, ATP citrate lyase (*ACLY*), and *FASN* in PT cells and some other tubule cells (Figure 7, A and B). *ACSS2* was almost exclusively expressed by the PT cells compared with all other cells in the kidney, and its expression level did not differ between healthy and diseased PT cells (39) (Supplemental Figure 7, A and B). We confirmed single-cell gene expression results using ISH in healthy and diseased human kidneys and found that *ACSS2* was highly expressed in PT cells, as we observed its coexpression with the PT marker megalin (*LRP2*) (Figure 7C). Consistent with single-cell expression data, the quantification of RNA ISH showed no change in *ACSS2* expression in PT cells in diseased kidneys (Figure 7D). Mouse kidney tissue samples followed the same expression pattern. Mouse kidney single-cell gene expression data (40) indicated a PT-specific expression pattern of *Acss2* without a major difference in its expression in diabetic mice (Supplemental Figure 7, C and D). *Acss2* was almost exclusively expressed in *Lrp2*^+^ PT cells (Figure 7E). Immunofluorescence to detect ACSS2 protein in human kidneys followed the ISH pattern (Figure 7F). In healthy human kidneys, FASN was mostly expressed in LTL^+^ (lotus tetragonolobus lectin) (proximal) tubule cells (Figure 7G). PLIN2 expression was enriched at the basolateral aspect of LTL^+^ (proximal) tubule cells (Supplemental Figure 7E). Protein levels of SCAP, FASN, PLIN2, NLRP3, and GSDMD were higher in fibrotic human kidney tissue samples, likely indicating that DNL could be associated with kidney disease (Figure 7, H and I).

## Discussion

Here, we identify ACSS2 as a human kidney disease risk gene via the integration of human genetic (GWAS), kidney QTL (eQTL and mQTL), and human kidney single-cell expression (snATAC-Seq) data. Using CRISPR KO mice and cultured kidney cell models, we show that ACSS2 controlled DNL and contributed to fibrosis development by regulating cellular redox state and inflammasome activation. Mice with tubule-specific deletion of FASN, the key rate-limiting enzyme in DNL, demonstrate the critical role of DNL in kidney tubule cells. Furthermore, our work identifies ACSS2 and FASN as potential pharmacological targets for kidney disease.

Drugs that target genes with genetic mutations that cause kidney disease have a higher chance of making it into the clinic (41). A recent large GWAS (12) that included information on 1.5 million participants identified over 800 risk loci for kidney dysfunction. It is a daunting task to translate these genetic discoveries into causal genes. Here, we used genetic mapping, kidney eQTL and mQTL, single-cell open chromatin information, and advanced statistical methods such as Bayesian colocalization, Mendelian randomization, and chromatin coaccessibility methods to prioritize *ACSS2* as a kidney disease risk gene on chromosome 20. This is a large, highly gene-dense region with close to 20 genes. We observed that the expression of genes at this locus showed a strong correlation with each other (12). Even after applying multiple gene prioritization methods, we were left with 3 genes prioritized by all available data sets and methods. Here, we focused on *ACSS2*. The analysis of the other genes at this locus should be the focus of future studies, as multiple genes exemplified by the *DPEP1* and *CHMP1A* locus (42), could be responsible for disease development at a single locus.

Abnormal lipid metabolism is an important feature of CKD (9, 43, 44). Kidney tubular cells almost exclusively consume fatty acids to liberate energy for the active reabsorption of filtered electrolytes and metabolites. Proper FAO and mitochondrial respiration events are crucial for normal kidney function (45). We and others (9, 11) had showed that both pathways were defective in patients with CKD. Consistent with these findings, we also observed lower expression of FAO genes. While ACSS2 has been proposed to play a role in FAO in the liver of mice on a high-fat diet (46), we did not observe changes in FAO in *Acss2*-KO kidneys or cells. ACSS2 has emerged as an important enzyme for histone modifications in brain cells (47), but histone modification changes were not evident in mouse kidneys. Future studies using cell-type–specific metabolomics could help clarify the role of ACSS2 in kidney PT cells.

There is growing interest in the role of fatty acid synthesis in fibrotic diseases such as those of the liver; however, its role has not been studied in the kidney (48). ACACA and FASN are the major rate-limiting enzymes controlling fatty acid synthesis. Increased *FASN* expression and activity have been consistently associated with steatosis-induced liver fibrosis (49) and bleomycin-induced pulmonary fibrosis (30). Inhibition of FASN activity by TVB-2640 in patients with nonalcoholic steatohepatitis (NASH) shows protection, and its effects are being studied in phase IIb clinical trials (50). In our study, we noted an increase in the expression of fatty acid synthesis pathway genes, including *FASN* and *ACACA*. Concomitantly, *SREBP1* and *SCAP* are upstream regulators of fatty acid synthesis that were also higher in injured kidneys. Deletion of *SCAP* or *FASN* in tubule cells or pharmacological inhibition of *FASN* protected cultured tubular cells from profibrotic gene expression and mice from tissue fibrosis, highlighting the importance of DNL in kidney disease development.

The mechanistic link between DNL and fibrosis is less understood (51). In this study, we demonstrate that fatty acid synthesis in tubular cells lowered the cellular reduced form of NADP and is associated with elevated mtROS production and fibrotic gene expression. Importantly, DNL signature genes were highly expressed by PT cells. Deletion of *ACSS2* suppressed *FASN* and *ACACA* expression in tubular cells, lowered the cellular redox state, and prevented the expression of profibrotic genes, including *Acta2*, *Col1a1*, *Col3a*, and *Fn1*. We also show that scavenging mtROS by MitoTempo diminished the expression of fibrotic genes. Inflammatory pathways (52, 53), including NLRP3-dependent pyroptosis, have been shown to play a role in kidney (54–56), liver (57), and heart (58) fibrosis by several earlier studies. We found that expression levels of NLRP3, GSDMD-N, and cleaved caspase 1 proteins in UUO kidneys were lower in the absence of ACSS2.

Most important, our study identifies a new pharmacologically targetable mechanism for kidney fibrosis. We show that not only did genetic deletion of *ACSS2*, *FASN*, and *SCAP* protect cells and mice from fibrosis development, but that pharmacological targeting of these pathways was also protective. We show that small molecules, such as FASNall, which targets FASN, and ACSS2i, which targets ACSS2, protected mice from developing kidney disease. It is reassuring that *Acss2^–/–^* mice did not have an observable phenotype at baseline, suggesting that pharmacological targeting of ACSS2 is probably safe. Our studies indicate that other compounds, such as ACC1 (*ACACA*) inhibitors (59), that are currently in clinical trials for NASH could also ameliorate fibrosis development and offer an entirely new class of drugs for kidney dysfunction.

However, there are potential limitations of our study. (a)The genetic locus will require further analysis, as our studies prioritized multiple potential causal genes in addition to *ACSS2*. (b) We believe additional mouse and cellular models will be essential to study the role of other genes at this locus. (c) Our genetic data indicated a protective effect for ACSS2, but the level of ACSS2 was already low in whole-kidney lysates. The loss of ACSS2 expression was likely the result of global loss of PT cells that express *ACSS2*. (d) Our study focused on the downstream mechanism of ACSS2 signaling, but not upstream pathways regulating ACSS2. (e) The generation of tubule-specific *Acss2*-KO mice will also be important to ensure that the effect was truly related to tubule-specific *ACSS2* expression.

In summary, our study identifies *ACSS2* as a kidney disease gene. We demonstrate that DNL and fatty acid synthesis play a key role in kidney fibrosis by regulating cellular redox status and inflammation. Finally, we present data indicating that pharmacological targeting of ACSS2 or DNL could be an important therapeutic strategy for the treatment of CKD.

## Methods

Additional details on methods can be found in the Supplemental Methods.

### Generation and maintenance of Acss2-KO and tubule-specific Fasn-KO mice.

*Acss2*-KO mice were generated by the CRISPR/Cas9 Mouse Targeting Core facility at the University of Pennsylvania. Two sgRNAs were generated with the Guide-it sgRNA In Vitro Transcription Kit (632635, Takara). KO mice were identified by standard tail-genotyping PCR. The genetic deletion was confirmed by ISH, RNA *in situ* hybridization, quantitative real-time PCR (qPCR), and Western blotting.

To generate tubule-specific *Fasn*-KO (*Fasn^fl/fl^ Ksp Cre*) mice, we procured mice harboring Cre recombinase under the tubule gene cadherin 16 promoter (*Ksp Cre*) from The Jackson Laboratory (stock no. 012237) (60). These mice were mated with mice with the *FASN*-floxed allele (gift of Clay F. Semenkovich, Washington University, St. Louis, Missouri, USA) (61). *Scap^fl/fl^* mice on s C57BL/6 genetic background was generated earlier (gift of Timothy F. Osborne, Johns Hopkins University School of Medicine, St. Petersburg, Florida, USA) (62).

### In vivo inhibitor studies.

For the inhibitor studies, littermate mice were randomly assigned to receive either FASNall or ACSS2i. A single 1 mg/mL dose of FASNall was given via i.p injection at 10 mg/kg body weight, 1 day prior to UUO surgery. Mice were sacrificed 3 days after UUO surgery, and their kidneys were harvested. ACSS2i was i.p. injected at 20 mg/kg body weight, 1 day prior to UUO surgery, followed by daily injections for 5 days. Mice were sacrificed on the seventh day after UUO surgery.

### Statistics.

Statistical analysis was performed using GraphPad Prism 9 (GraphPad Software). One-way ANOVA and 2-tailed unpaired *t* tests were performed on variables. No outliers were excluded in the in vivo study. Sample size estimation was not performed, and sample size was determined by the number of animals in the colony of a determined age and sex. The number of replicates (including the number of animals used in each experiment) is indicated in the figures and/or figure legends. All data are expressed as the mean ± SEM. The statistical parameters can be found in the figures and the figure legends. A *P* value of less than 0.05 was considered significant.

### Study approval.

All animal studies were approved by the IACUC of the University of Pennsylvania under protocol number 804138. The University of Pennsylvania’s IRB approved the human kidney sample collection. We engaged with an external honest broker, who was responsible for human kidney sample collection. No personal data were acquired.

### Data availability.

GWAS, kidney QTL, snATAC-Seq, and snRNA-Seq data were previously published (GCST90100220 [EMBL-EBI, NIH], GSE115098, GSE173343, GSE172008, and GSE200547) and are publicly available in NCBI’s Gene Expression Omnibus (GEO) database as well as the Susztak Laboratory Kidney Biobank (https://susztaklab.com/GWAS/index.php; https://susztaklab.com/Kidney_eQTL/index.php; https://susztaklab.com/Kidney_eQTL/index.php; https://susztaklab.com/Human_snATAC/index.php; https://susztaklab.com/hk_genemap/snRNA)/ Data values for bar graphs can be found in the Supplemental Supporting Data Values file. Full blots are presented in supplemental material. Additional details on protocols and special reagents for this study are provided by the corresponding author upon request.

## Author contributions

KS and DM conceived, planned, and oversaw the study. KS and DM analyzed the experimental data, prepared all figures, and wrote the manuscript. DM, LL, TD, LPK, and JW performed all experiments. DM, HL, and KS consolidated genetics data. EH, KK, and AA helped with transcriptomics analysis. SM helped with FAO tracing. PD and HH helped with *Acss2^–/–^* mouse generation. KF and PMT helped with manuscript editing. KU helped with DNL experiments. JAB and KEW provided critical suggestions during the study. DG generated *Scap^fl/fl^* mice on a C57BL/6 genetic background. All authors approved the final version of the manuscript.

## Supplementary Material

Supplemental data

Unedited blot and gel images

Supplemental table 1

Supplemental table 2

Supplemental table 3

Supporting data values

## Version 1 12/05/2023

In-Press Preview

## Version 2 02/15/2024

Electronic publication

## References

[1] Obrador GT (2017). Genetic and environmental risk factors for chronic kidney disease. Kidney Int Suppl (2011).

[2] Bikbov B (2020). Global, regional, and national burden of chronic kidney disease, 1990-2017: a systematic analysis for the Global Burden of Disease Study 2017. Lancet.

[3] Tian Z, Liang M (2021). Renal metabolism and hypertension. Nat Commun.

[4] Mukhi D (2017). Novel actions of growth hormone in podocytes: implications for diabetic nephropathy. Front Med (Lausanne).

[5] Herman-Edelstein M (2014). Altered renal lipid metabolism and renal lipid accumulation in human diabetic nephropathy. J Lipid Res.

[6] Stadler K (2015). The evolving understanding of the contribution of lipid metabolism to diabetic kidney disease. Curr Diab Rep.

[7] Susztak K (2005). Multiple metabolic hits converge on CD36 as novel mediator of tubular epithelial apoptosis in diabetic nephropathy. PLoS Med.

[8] Khan S (2018). Kidney proximal tubule lipoapoptosis is regulated by fatty acid transporter-2 (FATP2). J Am Soc Nephrol.

[9] Kang HM (2015). Defective fatty acid oxidation in renal tubular epithelial cells has a key role in kidney fibrosis development. Nat Med.

[10] Li S (2013). Proximal tubule PPARα attenuates renal fibrosis and inflammation caused by unilateral ureteral obstruction. Am J Physiol Renal Physiol.

[11] Miguel V (2021). Renal tubule Cpt1a overexpression protects from kidney fibrosis by restoring mitochondrial homeostasis. J Clin Invest.

[12] Liu H (2022). Epigenomic and transcriptomic analyses define core cell types, genes and targetable mechanisms for kidney disease. Nat Genet.

[13] Claussnitzer M, Susztak K (2021). Gaining insight into metabolic diseases from human genetic discoveries. Trends Genet.

[14] Tam V (2019). Benefits and limitations of genome-wide association studies. Nat Rev Genet.

[15] Qiu C (2018). Renal compartment-specific genetic variation analyses identify new pathways in chronic kidney disease. Nat Med.

[16] Sheng X (2021). Mapping the genetic architecture of human traits to cell types in the kidney identifies mechanisms of disease and potential treatments. Nat Genet.

[17] Pliner HA (2018). Cicero Predicts cis-regulatory DNA interactions from single-cell chromatin accessibility data. Mol Cell.

[18] Nasser J (2021). Genome-wide enhancer maps link risk variants to disease genes. Nature.

[19] Giambartolomei C (2014). Bayesian test for colocalisation between pairs of genetic association studies using summary statistics. PLoS Genet.

[20] Zhu Z (2016). Integration of summary data from GWAS and eQTL studies predicts complex trait gene targets. Nat Genet.

[21] Ko YA (2017). Genetic-variation-driven gene-expression changes highlight genes with important functions for kidney disease. Am J Hum Genet.

[22] Yang HC (2010). Models of chronic kidney disease. Drug Discov Today Dis Models.

[23] Yan LJ (2021). Folic acid-induced animal model of kidney disease. Animal Model Exp Med.

[24] Sivanand S (2018). Spatiotemporal control of Acetyl-CoA metabolism in chromatin regulation. Trends Biochem Sci.

[25] Moffett JR (2020). Acetate revisited: a key biomolecule at the nexus of metabolism, epigenetics, and oncogenesis - Part 2: acetate and ACSS2 in health and disease. Front Physiol.

[26] Mendoza M (2022). Enzymatic transfer of acetate on histones from lysine reservoir sites to lysine activating sites. Sci Adv.

[27] Batchuluun B (2022). Lipogenesis inhibitors: therapeutic opportunities and challenges. Nat Rev Drug Discov.

[28] Wilson MH (2021). Imaging cytoplasmic lipid droplets in vivo with fluorescent perilipin 2 and perilipin 3 knock-in zebrafish. Elife.

[29] Alwarawrah Y (2016). Fasnall, a selective FASN inhibitor, shows potent anti-tumor activity in the MMTV-Neu model of HER2(+) breast cancer. Cell Chem Biol.

[30] Wang H (2022). Therapeutic efficacy of FASN inhibition in preclinical models of HCC. Hepatology.

[31] Mews P (2019). Alcohol metabolism contributes to brain histone acetylation. Nature.

[32] Bidault G (2021). SREBP1-induced fatty acid synthesis depletes macrophages antioxidant defences to promote their alternative activation. Nat Metab.

[33] Doke T (2021). Genome-wide association studies identify the role of caspase-9 in kidney disease. Sci Adv.

[34] Doke T, Susztak K (2022). The multifaceted role of kidney tubule mitochondrial dysfunction in kidney disease development. Trends Cell Biol.

[35] Ma X, Ding WX (2021). A fluorescence imaging based-assay to monitor mitophagy in cultured hepatocytes and mouse liver. Liver Res.

[36] Devant P (2023). Gasdermin D pore-forming activity is redox-sensitive. Cell Rep.

[37] Evavold CL (2021). Control of gasdermin D oligomerization and pyroptosis by the Ragulator-Rag-mTORC1 pathway. Cell.

[38] Alfonso-Loeches S (2014). Role of mitochondria ROS generation in ethanol-induced NLRP3 inflammasome activation and cell death in astroglial cells. Front Cell Neurosci.

[39] Wilson PC (2022). Multimodal single cell sequencing implicates chromatin accessibility and genetic background in diabetic kidney disease progression. Nat Commun.

[40] Wu H (2022). Mapping the single-cell transcriptomic response of murine diabetic kidney disease to therapies. Cell Metab.

[41] King EA (2019). Are drug targets with genetic support twice as likely to be approved? Revised estimates of the impact of genetic support for drug mechanisms on the probability of drug approval. PLoS Genet.

[42] Guan Y (2021). A single genetic locus controls both expression of DPEP1/CHMP1A and kidney disease development via ferroptosis. Nat Commun.

[43] Rinaldi A (2022). Impaired fatty acid metabolism perpetuates lipotoxicity along the transition to chronic kidney injury. JCI Insight.

[44] Kimmelstiel P, Wilson C (1936). Intercapillary lesions in the glomeruli of the kidney. Am J Pathol.

[45] Jang HS (2020). Defective mitochondrial fatty acid oxidation and lipotoxicity in kidney diseases. Front Med (Lausanne).

[46] Huang Z (2018). ACSS2 promotes systemic fat storage and utilization through selective regulation of genes involved in lipid metabolism. Proc Natl Acad Sci U S A.

[47] Mews P (2017). Acetyl-CoA synthetase regulates histone acetylation and hippocampal memory. Nature.

[48] Kume S (2007). Role of altered renal lipid metabolism in the development of renal injury induced by a high-fat diet. J Am Soc Nephrol.

[49] Jensen-Urstad AP, Semenkovich CF (2012). Fatty acid synthase and liver triglyceride metabolism: housekeeper or messenger?. Biochim Biophys Acta.

[50] Loomba R (2021). TVB-2640 (FASN inhibitor) for the treatment of nonalcoholic steatohepatitis: FASCINATE-1, a randomized, placebo-controlled phase 2a Trial. Gastroenterology.

[51] Moeller MJ (2021). New aspects of kidney fibrosis-from mechanisms of injury to modulation of disease. Front Med (Lausanne).

[52] Mohandes S (2023). Molecular pathways that drive diabetic kidney disease. J Clin Invest.

[53] Doke T (2023). NAD^+^ precursor supplementation prevents mtRNA/RIG-I-dependent inflammation during kidney injury. Nat Metab.

[54] Wu J (2021). APOL1 risk variants in individuals of African genetic ancestry drive endothelial cell defects that exacerbate sepsis. Immunity.

[55] Gu X (2021). Kidney disease genetic risk variants alter lysosomal beta-mannosidase (*MANBA*) expression and disease severity. Sci Transl Med.

[56] Balzer MS (2022). Single-cell analysis highlights differences in druggable pathways underlying adaptive or fibrotic kidney regeneration. Nat Commun.

[57] Gaul S (2021). Hepatocyte pyroptosis and release of inflammasome particles induce stellate cell activation and liver fibrosis. J Hepatol.

[58] Olsen Maria B (2022). Targeting the inflammasome in cardiovascular disease. JACC Basic Transl Sci.

[59] Kim CW (2017). Acetyl CoA carboxylase inhibition reduces hepatic steatosis but elevates plasma triglycerides in mice and humans: a bedside to bench investigation. Cell Metab.

[60] Shao X (2002). Epithelial-specific Cre/lox recombination in the developing kidney and genitourinary tract. J Am Soc Nephrol.

[61] Chakravarthy MV (2005). “New” hepatic fat activates PPARalpha to maintain glucose, lipid, and cholesterol homeostasis. Cell Metab.

[62] Guan D (2018). Diet-induced circadian enhancer remodeling synchronizes opposing hepatic lipid metabolic processes. Cell.

