# Supplementary material for: ACSS2 gene variants determine kidney disease risk by controlling de novo lipogenesis in kidney tubules
Source: J Clin Invest. 2023 Dec 5;134(4):e172963. doi: 10.1172/JCI172963 (PMC10866669; doi:10.1172/JCI172963)
Supplement: Supplemental data [file jci-134-172963-s268.pdf]

Supplemental Information

ACSS2 gene variants determine kidney disease risk by controlling de novo lipogenesis in kidney tubules

Dhanunjay Mukhi[1,2,3], Lingzhi Li[1,2,3], Hongbo Liu[1,2,3], Tomohito Doke[1,2,3], Lakshmi P. Kolligundla, Eunji Ha[1,2,3], Konstantin Kloetzer[1,2,3], Amin Abedini[1,2,3], Poonam Dhillon[1,2,3], Sarmistha Mukherjee[2,4], Junnan Wu[1,2,3], Hailong Hu[1,2,3], Dongyin Guan[5], Katsuhiko Funai[6], Kahealani Uehara[2,4], Paul M. Titchenell[2,4], Joseph A. Baur[2,4], Kathryn E. Wellen[7,8], Katalin Susztak[1,2,3,9*]

Inventory for Supplemental Information

I. Supplemental methods

II. References

III. Supplemental Figure1. Prioritization of ACSS2 as a kidney disease risk gene.

IV. Supplemental Figure 2. Loss of ACSS2 protects from kidney disease.

V. Supplemental Figure 3. TGF-β1 induces de novo lipogenesis in primary tubule cells.

VI. Supplemental Figure 4. Inhibition of de novo lipogenesis prevents kidney fibrosis.

VII. Supplemental Figure 5. Suppression of mitochondrial ROS suppresses NLRP3-inflammasome activation in primary tubular cells.

VIII. Supplemental Figure 6. Inhibition of de novo lipogenesis suppresses ROS-induced NLRP3 inflammasome activation.

IX. Supplemental Figure 7. Fatty acid synthesis correlated with fibrosis in CKD patients.

X. Supplementary Table 1: Gene prioritization table.

XI. Supplementary Table 2: Prioritized SNPs.

XII. Supplementary Table 3. Guide RNAs and human primers.

XIII. Supplementary Table 4: Mouse gene primers.

Supplemental methods

Prioritization of causal genes for eGFR GWAS loci

To prioritize target genes for eGFR GWAS loci on chr 20, we employed a priority scoring strategy by integrating eight different datasets: (1) microdisected kidney tubule and glomeruli eQTL data sets (https://susztaklab.com/Kidney_eQTL/index.php) (significant SNP~gene associations, FDR<0.05)(1, 2); (2) mQTL (significant SNP~CpG~gene associations, FDR<0.05) and eQTM (CpG level FDR <0.05) analysis (https://susztaklab.com/Kidney_meQTL/index.php) (3); (3) colocalization between eGFR GWAS and eQTL (H4 > 0.8); (4) multiple colocalization (moloc) analysis(3) of SNP~gene pairs between eGFRGWAS, eQTL and mQTL (PPA.abc > 0.8); (5) summary mendelian randomization for the SNP~gene pairs between eGFR GWAS and eQTL (PSMR < 1.38×10-4); (6) SNP~gene pairs passing HEIDI test between eGFR GWAS and eQTL (PHEIDI > 0.01); (7) Cicero co-accessibility interactions data from 57,262 snATAC-seq cells (co-accessibility score > 0.2)(https://susztaklab.com/Human_snATAC/index.php) (2, 3); and (8) Element-gene connections identified by Activity-by-Contact (ABC) model which predicts enhancers regulating genes based on estimating enhancer activity and enhancer-promoter contact frequency from epigenomic datasets (ABC scores >= 0.015)(4). Promoters were defined as ±2000bp from the TSS of protein-coding transcripts from GENCODE v35lift3765 to annotate Cicero connections or Element-gene connections between gene promoters and eGFR GWAS variants. eGFRcrea GWAS meta-analysis, (n=1,508,659 individuals), DNA methylation data (n=506 human kidneys), cis-eQTL, kidney mQTL (n=686 individuals) and Bayesian colocalization,

and summary Mendelian randomization analyses were performed according to previous publication (2, 3).

Human kidney single nuclear ATAC-sequencing

Adult human kidney single nuclear ATAC seq- data was used from previous publications (2, 3). The data can be viewed at the website (http://www.susztaklab.com/Human_snATAC/index.php).

Animal studies

The mice were fed *ad libitum* with water and rodent standard chow diet. 6- to 7-week-old male and female C57BL/6J mice were used in the study. Mice were randomly assigned to experimental groups for all experiments including drug efficacy studies. To induce kidney injury, we employed two widely used kidney disease models including unilateral ureteral obstruction (UUO) and folic acid nephropathy (FAN). UUO surgery experiments were conducted on male and female mice. Briefly, UUO was performed by ligating the right kidney ureter, and the left kidney served as a sham operated kidney. Post-surgical procedures were followed according to the IACUC guidelines. For FAN models, 300mM sodium bicarbonate ($NaHCO_3$) solution was first made to solubilize folic acid (FA) and injected into male mice (FA 250 mg/kg i.p at single dose). Mice were sacrificed 7 days following injection or surgery. For the drug injection studies, mice were first injected one day before the UUO surgery.

Adenine induced chronic kidney disease model

Adenine (#A11500) was purchased from RPI (Saint Louis, MO, USA). Adenine was dissolved in water at a concentration 2.5 mg/ml. Male mice were given adenine by oral gavage at a dose of 50mg/kg body weight daily for four weeks (5). Control male mice received 0.2ml of vehicle every day for four weeks. Animals were sacrificed on day 30. BUN and serum creatinine was analyzed

by BUN (#B7552-150, Horiba Pointe Scientific) and creatinine kits (#DZ072B-KY1, Diazyme). Daily body weights were recorded.

Crispr SNP deletion experiments

Two Crispr guides were generated for the open chromatin of the prioritized SNP region. Guide RNAs were designed using Crispor software(6) and cloned into pLKO5.SgRNA.EFS.GFP plasmid (#57822, Addgene) using Esp3I restriction enzyme (#FD0454, Thermo). Guide RNA sequences and human primers were listed in Supplemental table 3. Bacterial transformation was performed using OneShot-Stbl3 competent cells (#C737303, Thermo) and isolated plasmids were verified by Sanger sequencing. The guide RNA containing plasmids were transfected into HEK293 cells stably expressing Cas9 (gift from Dr. Liling Wan, University of Pennsylvania) using Lipofectamine 3000 (#L300015, Thermo). After 72h of transfection, puromycin (4µg/ml) was added for an additional 3 days. The cells were harvested, and RNA, and DNA were isolated. The DNA was cloned into TOPO-TA vector and TOP10 chemical competent cells (#K4500J10, Thermo). Genomic region deletion was further verified by Sanger sequencing. The isolated RNA was used to measure gene expression by quantitative real time PCR.

Gene expression analysis

A total of 15mg of kidney tissue was homogenized in 1ml of Trizol (Ambion) with Qia Tissue Lyzer for 1min 15sec at 4°C. After homogenization in Trizol, 200ul of chloroform was directly dispensed into the Trizol lysate and vortexed for 15sec at RT. Lysates were then spun down at 12000rpm for 15min at 4oC, and the upper aqueous layer was collected into new, clean tubes. Next, 500ul of isopropanol (100%) was slowly added through the wall of the tubes and mixed gently. The tubes were then spun down at 12500rpm at 4°C for 15min, and the pellet was washed with 70%

ethanol (made from clean 100% ethanol) at 10000rpm for 10min at 4oC. Finally, the RNA pellet was dried at RT for 15min, resuspended in clean RNase free and Dnase free water. RNA was pretreated with DNase before proceeding to cDNA conversion. A total of 2,000 ng RNA was converted into cDNA using the High-capacity cDNA Reverse Transcription Kit (#4368813, Applied Biosystems). Realtime quantitative PCR analysis was performed to measure the relative gene expression by normalizing the CT values of gene of interest with endogenous control gene (*Gapdh* was used). The data was calculated and presented as fold change by ($2^{-\Delta\Delta CT}$ method). Primer sequence listed in Supplemental table 4.

Western blotting

Approximately 20-30mg of kidney tissue was homogenized in SDS-blue loading buffer (#7722, CST) containing 42mM DTT. Samples were loaded onto the SDS-PAGE gels and run at 100v for 1h 40min at RT in Tris-Glycine-SDS buffer. The proteins were transferred onto a PVDF membrane. Membranes were blocked with 3% non-fat dry milk powder in tris-buffer saline containing Tween-20 (TBST) for 30min at RT. The blots were then incubated with primary antibody overnight at 4°C. The primary antibodies used were anti-ACSS2 (#ab66038, abcam), anti-α-SMA (#A5228, Sigma), anti-fibronectin (#ab2413, abcam), anti-NLRP3 (#AG-20B-0014C100, Adipogen), anti-cleaved GSDMD (#36425S, CST), anti-human GSDMD (#sc-393656, Santa Cruz), anti-CASPASE1 (#AG-20B-0042-C100, Adipogen), anti-FASN (#3180S, CST), anti-PLIN2 (#PA5-29099, Invitrogen), anti-SCAP (#PA5-28982, Invitrogen), anti-KIM1 (#PA5-79345, Invitrogen), anti-LC3 (#2775S, CST), anti-PARK2 (#sc-32282, Santa Cruz), and anti-GAPDH (#2118S, CST). After primary antibody incubation, the blots were washed three times with TBST, IRdye-conjugated secondary antibodies were probed for 1h at RT. The secondary antibodies used were anti-rabbit IgG (H+L) (DyLight™ 800 4X PEG Conjugate, #5151S, CST) and anti-mouse IgG (H+L) (DyLight™ 680 Conjugate, #5470S, CST). Finally, the blots were washed in

TBST for three minutes each, 10min at RT, and scanned at 600, 700, and 800 excitation wavelengths in Li-COR imager (Odyssey® XF), Image Studio software. The images were quantified for relative abundance in Image J software.

Immunofluorescence staining

Immunofluorescence performed as previously described(7). Briefly, 5µm thin formalin-fixed paraffin-embedded kidney cortical sections were deparaffinized and rehydrated using ethanol gradients from 100%-70%. Slides were preheated in a 10mM citrate buffer containing 0.1% triton X-100 to retrieve the target antigen. The slides were blocked with PBS containing 10% BSA and 0.1% Tween for 1h at RT. The slides were incubated overnight at 4C with primary antibodies prepared in PBS. The following primary antibodies were used: anti-Ki67 1:50 dilution (#9129S, CST), anti-ACSS2 1:50 dilution (#ab66038, abcam), anti-FASN at 1:50 dilution (#3180S, CST), and anti-Perilipin2 at 1:50 dilution (#PA5-29099, Invitrogen). LTL-Fluorescein (FL-1321, Vector labs) was used to label PT cells. The slides were then incubated at 37OC with anti-rabbit Alexa Fluor 488 (#A-21200, Invitrogen), and anti-mouse Alexa Fluor 594 (#A-31572, Invitrogen). Finally, the sections were stained with DAPI containing anti-fade mounting media (#P36941, Invitrogen).

Histone extraction and western blotting

To extract histones from kidneys, we first isolated nuclei and proceeded with acid-histone extraction method (8). Briefly, we washed nearly 40mg of kidney tissue with ice-cold PBS and minced it in the nuclei isolation buffer (NIB-250 is 15mM Tris-HCl at pH 7.5, 60mM KCl, 15mM NaCl, 5mM MgCl2, 1mM CaCl2, and 250mM sucrose, to which 0.1% Nonidet P-40, 1x protease inhibitor cocktail, 1mM DTT, and 10mM sodium butyrate). We collected the kidney pieces into glass Dounce homogenizer on ice. After 5 min of incubation on ice, we spun down the

homogenates, collected the nuclei pellet, washed it twice with NIB-250 without Nonidet P-40 and proceeded with histone extraction.

We incubated the nuclear pellet with 0.4 N H_2SO4 at a 5:1 ratio for 2h at $4^{O}C$. We then spun down the acidified nuclei at 11000rcf for 10min at $4^{O}C$ and collected the soluble fraction containing histones into a new tube and precipitated with 20% trichloroacetic acid at final concentration overnight at $4^{O}C$. We spun down the samples at 11000rcf for 10min at $4^{O}C$ to sediment the histone pellet at the bottom of the tube. We then washed the histone pellets with 1ml of ice-cold acetone containing 0.1% 12N HCl, followed by two washes with ice-cold 100% acetone. We air-dried the pellet and dissolved them in RIPA buffer. Two micrograms of histone lysates were loaded onto 15% SDS-PAGE gels. Western blotting was performed as described above and probed with anti-H3K27ac (#ab177178, abcam) and anti-total H3 (#4620, CST) antibodies. Imaged in Li-COR imager (Odyssey® XF).

H&E and Sirius Red staining

The tissues were fixed in formalin, dehydrated by an ethanol gradient (30%, 50%, 75% and 95%) and then submitted to the histology Core in 100% ethanol. Once the tissue was sectioned, H&E and Sirius red staining was performed. Images were acquired in Olympus 5000 microscope with Cell Sense software. Percentage of relative fibrosis was quantified in image J.

Primary tubular epithelial cell isolation and *in vitro* experiments

Primary kidney tubular epithelial cells (TECs) were isolated from young pups (2.5-3wk old) of WT, *Acss2*$^{-/-}$, *Fasnf/-* and *Scapf/f* mice. Kidneys were collected on a petri dish on ice, minced in RPMI media (Corning), and then then digested with 200ug/ml collagenase IV (1mg/ml, calbiochem) at

37°C for 30min. Collagenase was inactivated by adding 100µl of fetal bovine serum (100% FBS), and cells were passed through 100µm, 70µm and finally 40µm strainers. Cells were then centrifuged at 1000rpm for 5min at 4°C. The cell pellet was resuspended in 1 ml of sterile ice-cold RBC lysis buffer (Hy-Clone) and incubated for 2min on ice. Lysis was inhibited by adding ice-cold PBS and then centrifuged at 1000rpm for 10min at 4°C. Finally, the cell pellet was resuspended in RPMI complete media (10% FBS with antibiotics 1X ITS and 50ng/ml human EGF) and plated in 10cm dishes. Cells were grown in the incubator at 5% CO2 at 37°C, and the medium was changed every other day.

Cells were serum restricted in 0.5% FBS for 24h. Cells were then treated with 20ng/ml TGF-β1 for 48h in the presence or absence of FASNall (4µM/ml) or TVB-3664 (10nM/ml) in 0.5% serum media for 48h. *Fasnf/- or Scapf/f* cells were treated with adenovirus Ad5CMV-eGFP (Ad-GFP) or Ad5CMVCre-eGFP (Ad-Cre-GFP) (University of Iowa Gene Transfer Vector Core, Iowa City, IA) at a concentration of 0.5µl/ml for 24h in serum free media, and the infection efficiency was assessed by observing GFP signal under a fluorescence microscope before every experiment.

For, *siRNA* transfection experiments, a smart pool of non-target control *siRNA* and mouse *siFasn* were purchased from Dharmacon (#L-040091-00-0005, Horizon Biosciences). Cells were seeded in 6-well plates, grown overnight at 80-90% confluent, and then transfected with 20pM *siFasn* in RNAimax in OptiMEM for 48h. After transfection, cells were treated with TGF-β1 (20ng/ml) for 48h in 0.5% serum containing media. RNA or protein was isolated from these cells, and knockdown efficiency was determined by quantifying the relative *Fasn* expression using real time qPCR.

Cholesterol measurement

Total kidney cholesterol was quantitatively estimated using established methods (#K603-100, BioVision). Approximately 10-15mg of kidney tissue was homogenized in 300 µl of chloroform:

isopropanol: NP-40 (7:11:0.1) in a microcentrifuge. The homogenate was centrifuged at 15000g for 10min, and the liquid layer was collected into a new microtube. The supernatant was air dried at 50°C to remove chloroform and the samples kept under vacuum pressure (SpeedVac, Thermo Scientific) for 30 min to remove trace organic solvent. The dried lipids were dissolved in 200 µl of assay buffer and performed cholesterol measurements as per the manufacturer protocol.

^{3}H labeled FAO measurements in mice kidneys

FAO measurements were performed by tracing tritium labeled water ($^{3}H_{2}O$) (9). Frozen whole kidney extracts prepared in Krebs-Ringer bicarbonate buffer containing HEPES (#K4002, Sigma). Nearly 500µg of protein was used for FAO measurements. Briefly, the kidney homogenates were incubated with master cocktail (Krebs-Ringer bicarbonate buffer containing 100mg/ml fatty acid free BSA, 2.5mM palmitic acid, 10mM carnitine, and 4µCi of 9,10-3H-palmitoyl-CoA) for 2h at 37OC and 600rpm in dark. The homogenates were then subjected to Folch's lipid extraction protocol (2:1 chloroform and methanol) and further precipitated with 10% trichloro acetic acid (#T6399, Sigma). After high-speed centrifugation at 4C, 1ml of supernatants were passed through activated AG 1-X8 resin formate columns (#7316221, BioRad) and eluted in roughly 1ml volume (^{3}H2O) into glass vials. Nearly 500ul of elutes were mixed into 3ml of scintillation cocktail and read in a liquid scintillation counter (Beckman Coulter). The radioactive counts were subtracted from the sample containing no protein and from sample with cold palmitic acid (#P5585, Sigma). The final counts were normalized to the control samples and presented as relative FAO rate.

In vitro palmitic acid oxidation test by Seahorse analyzer

Real-time fatty acid oxidation analysis was performed in renal tubule cells using an XF-96 Extracellular Flux Analyzer with the Palmitate Oxidation Stress Test Kit and FAO Substrate

(#102720-100, Agilent Seahorse Bioscience). Briefly, primary tubule cells were isolated from WT and *Acss2*$^{-/-}$ mice and were cultured in a Seahorse 96-well plate at a density of 5x10^3 cells per well. The day before performing the OCR analysis, the cell culture medium was replaced with substrate-limited medium (DMEM (#A14430-01), 0.5 mM glucose, 1 mM Glutamax, 0.5 mM carnitine, and 1% FBS) and maintained up to 18 hours. An hour before performing the OCR analysis, the substrate-limited medium was exchanged with FAO assay medium (1x potassium bicarbonate buffer with 2.5 mM glucose, 0.5 mM carnitine, and 5 mM HEPES). Etomoxir (40 µM) was added 15 minutes before the start of the OCR analysis to the specified wells. Control cells were supplemented with BSA (0.17 mM) while test cells were supplemented with 1 mM palmitic acid conjugated BSA (0.17 mM). The OCR analysis was performed by treating cells with 2 µM oligomycin, 1 µM fluoro-carbonyl cyanide phenylhydrazone (FCCP), and 0.5 µM rotenone plus 1 µM antimycin A at final concentration.

In vivo DNL tracing with deuterated water

DNL tracing was performed at the GC-MS core at the University of Pennsylvania(10). To assess total lipogenesis, mice were subjected to UUO for seven days. On the 6th day, mice were fasted overnight at ~7 pm. On the 7th day, the mice were refed for three hours and then injected with 400ul of deuterated water (#151882, Sigma) prepared in 0.9% saline via i.p injection and continued feeding for three more hours. Systemic blood was collected by cardiac puncture, and livers and kidneys were harvested using clamps pre-cooled in liquid nitrogen. The blood was allowed to coagulate on ice for 15 min, and spun down at 10,000g for 5 min at 4C to collect serum. The frozen liver and kidney samples were ground at liquid nitrogen temperature with a Cryomill (Qiagen). Saponification of lipids and gas chromatography–mass spectrometry (GC–MS) analysis were performed at the GC-MS core. Briefly, 5µl of serum, and 100 mg of liver or kidney powder was saponified, and fatty acids were extracted by adding 0.5 ml of hexane, vortexing, and

transferring the top hexane layer to a new glass vial. Separation was performed by reversed-phase ion-pairing chromatography on a C8 column coupled to negative-ion mode, full-scan GC–MS at 1-Hz scan time and 100,000 resolving power (Agilent 7890A Gas Chromatograph; 5975 Mass Spectrometer; Thermo Fischer Scientific). Palmitate was analyzed using GC-MS, and the absolute amount of newly made palmitate was assumed equivalent to the rate of DNL. Data analysis with MAVEN software and natural isotope correction were performed by the GC-MS core.

Kidney Triglycerides quantification

Kidney triglycerides were measured using Triglyceride Calorimetric Assay kit (#10010303, Cayman). Approximately 20mg of kidney tissue was homogenized in NP40-substitute assay buffer containing protease and phosphatase inhibitors. Homogenates were collected after centrifuging at 10,000g for 10min at 4°C.

Oil Red O staining

For Oil Red O staining, we cut 5μm thin frozen sections. Briefly, the sections were dried at RT for 15min and fixed in prechilled 10% formalin buffered PBS for 10min. Slides were washed three times with water for 5min, and finally rinsed in 60% isopropanol for 5min. Lipids were stained by incubating slides in fresh Oil Red 'O' working solution for 30-60min at RT and rinsed in 60% isopropanol for five seconds. Slides were washed three times with water and counterstained with hematoxylin for 3min. Finally, slides were washed in 70% ethanol and mounted with 90% glycerol, and immediately proceeded for microscopic analysis. Images were acquired in EVOS FL inverted fluorescence microscope (#12-563-460, Invitrogen).

NADPH/NADP+ ratio measurements

The NADPH/NADP+ ratio was calculated according to the manufacturer protocol (#G1009, Promega). Briefly, an equal number of cells were cultured in a 96-well format and starved overnight for TGF-β1 treatments. Next, the cells were lysed in 20% DTAB containing Basic solution for 10min at RT. Samples were processed according to the protocol for measuring NADPH and NADP+ from the same well simultaneously. The absolute and relative NADPH/NADP+ values were calculated according to the manufacturer's formula.

GSH/GSSH ratio

Reduced and oxidized glutathione levels were measured according to the Glutathione Colorimetric Detection Kit (#EIAGSHC, Thermo Scientific) from the same well after completion of all treatments.

Mitochondrial Quality assessments and Mitophagy

Primary cells were cultured on microscopic cover glass overnight until they reached 70% confluency. Cells were incubated in a 5μM MitoSox (#M36008, Thermo Scientific) solution for 10min in incubator and processed for imaging and quantifications. Cells were also incubated in 10μM JC-1 (#T3168, Thermo Scientific) for 10min and then proceeded with imaging and quantifications.

Mitophagy was assessed as described in our earlier paper (11). Primary cells were transfected with Mito 'Q' mCherry-eGFP COX8 plasmid (#78520, Addgene) for 48h. Cells were processed for various treatments and proceeded with imaging.

Cells cultured as above and pretreated with 100μM MitoTempo (#SML0737, Sigma) for 2h, followed by TGF-β1 treatments while MitoTempo was still present. The images of JC-1, mitosox stainings, Mito 'Q' mCherry-eGFP COX8, and MitoTempo experiments were all acquired in Olympus 5000 microscope with Cell Sense software.

***In situ* hybridization**

In situ hybridization was performed on formalin-fixed paraffin-embedded tissue sections using the RNAscope 2.5 HD Duplex Detection Kit (#322436, ACD Bio) according to the manufacture protocol. Freshly cut kidney tissue sections were used in all *in situ* experiments. For the Gsdmd *in situ* hybridization, the antigen retrieval was performed for 30min in heated water bath. The following probes were used for the RNAscope *in situ* assay: Mm Acss2, Hs-ACSS2, Mm-Gsdmd, Mm-Lrp2, Hs-LRP2 and Mm-Hnf4a. *In situ* hybridization quantification was performed manually according to the ACD Bio RNAscope 2.5 HD Duplex user manual.

Human kidney bulk RNA-seq, and single nuclear RNA sequencing data (previously generated) can be viewed at the http://www.susztaklab.com/hk_genemap/scRNA website.

References

1. Qiu C, et al. Renal compartment-specific genetic variation analyses identify new pathways in chronic kidney disease. *Nat Med.* 2018;24(11):1721-1731.
2. Sheng X, et al. Mapping the genetic architecture of human traits to cell types in the kidney identifies mechanisms of disease and potential treatments. *Nat Genet.* 2021;53(9):1322-1333.

3. Liu H, et al. Epigenomic and transcriptomic analyses define core cell types, genes and targetable mechanisms for kidney disease. *Nat Genet.* 2022;54(7):950-962.

4. Nasser J, et al. Genome-wide enhancer maps link risk variants to disease genes. *Nature.* 2021;593(7858):238-243.

5. Rahman A, et al. A novel approach to adenine-induced chronic kidney disease associated anemia in rodents. *PLoS One.* 2018;13(2):e0192531.

6. Concordet JP, and Haeussler M. CRISPOR: intuitive guide selection for CRISPR/Cas9 genome editing experiments and screens. *Nucleic Acids Res.* 2018;46(W1):W242-W245.

7. Mukhi D, et al. Growth hormone induces transforming growth factor-beta1 in podocytes: Implications in podocytopathy and proteinuria. *Biochim Biophys Acta Mol Cell Res.* 2023;1870(2):119391.

8. Zhao S, et al. ATP-Citrate Lyase Controls a Glucose-to-Acetate Metabolic Switch. *Cell Rep.* 2016;17(4):1037-1052.

9. Mukherjee S, et al. SIRT3 is required for liver regeneration but not for the beneficial effect of nicotinamide riboside. *JCI Insight.* 2021;6(7).

10. Wan M, et al. Postprandial hepatic lipid metabolism requires signaling through Akt2 independent of the transcription factors FoxA2, FoxO1, and SREBP1c. *Cell Metab.* 2011;14(4):516-527.

11. Doke T, et al. Genome-wide association studies identify the role of caspase-9 in kidney disease. *Sci Adv.* 2021;7(45):eabi8051.

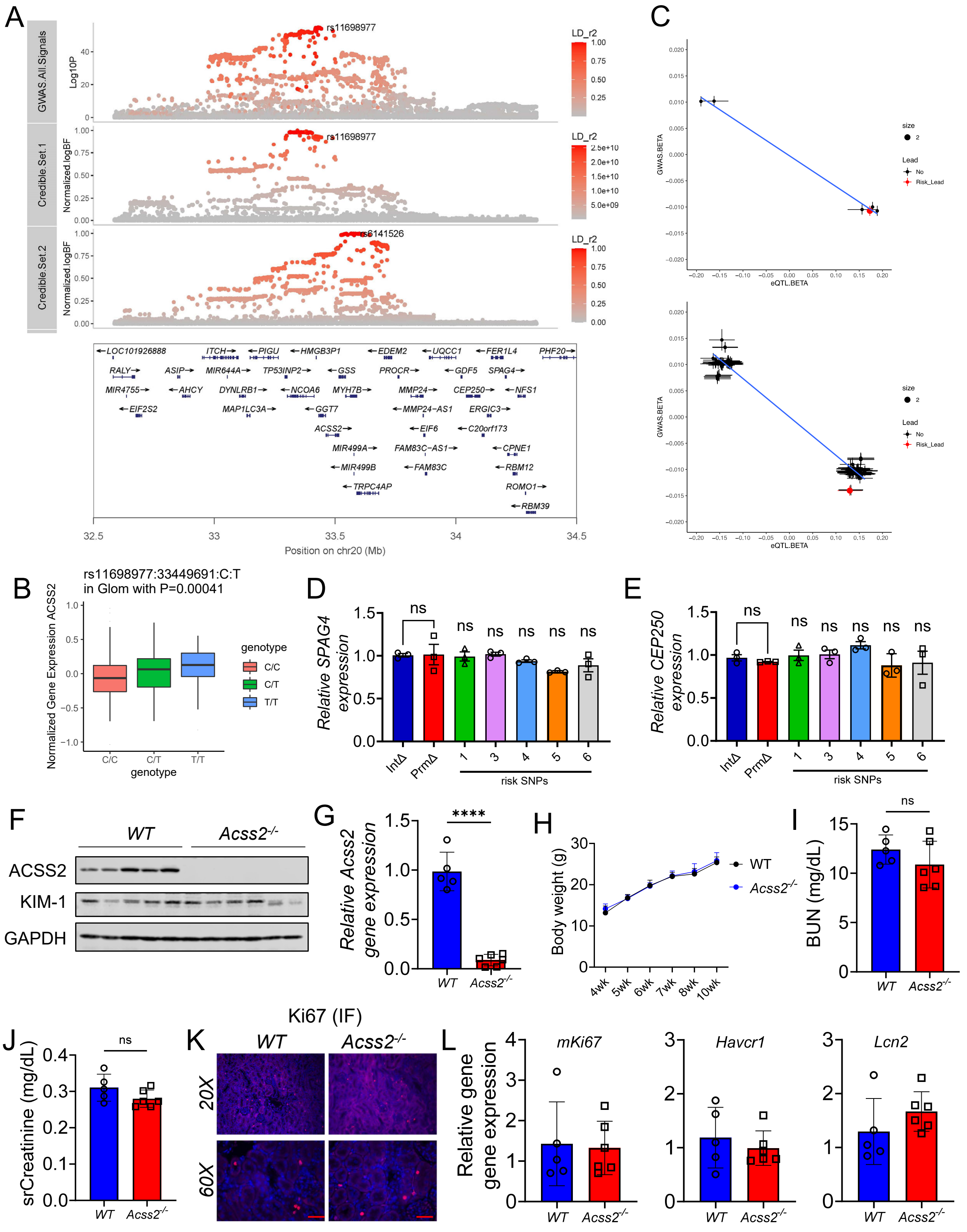

Supplemental Figure 1. Prioritization of ACSS2 as a kidney disease risk gene.

A. Fine mapping regional plot showing single nucleotide variants associated with kidney eGFR GWAS dataset (N=1.5M European population). X-axis chromosomal location and y-axis shows the strength of association (-log(p)). Variants shown in red color indicates correlation (extreme right r2) with underlying genes. Color key indicates linkage disequilibrium (r2).

B. Human kidney *ACSS2* gene expression in glomeruli (n=303) in microdissected samples. Y -axis shows normalized *ACSS2* expression and X-axis shows genotype information.

C. GWAS and eQTL effect sizes (upper plot tubule; lower plot glom) plotted for *ACSS2* gene in 1.5M human samples.

D. Transcript levels of *CEP250* following risk regions deleted in HEK293T cells. The data was generated using samples used in Figure 1I.

E. Transcript levels of *SPAG4* following risk regions deleted in HEK293T cells. The data was generated using samples used in Figure 1I.

F. Immunoblots of ACSS2, KIM-1 and GAPDH in whole kidney lysates of wild type (*WT)* (n=5) and *Acss2*$^{-/-}$ mice (n=6).

G. Transcript levels of *Acss2* in kidneys of *WT* (n=5) and *Acss2*$^{-/-}$ mice (n=6).

H. Weekly body weights recorded in *WT* (n=5) and *Acss2*$^{-/-}$ mice (n=6) at baseline.

I. Blood urea nitrogen (BUN) in 10 weeks old *WT* (n=5) and *Acss2*$^{-/-}$ (n=6) mice at baseline.

J. Serum creatinine (sCr) in 10 weeks old *WT* (n=5) and *Acss2*$^{-/-}$ (n=6) mice at baseline.

K. Ki67 immunofluorescence in *WT* and *Acss2*$^{-/-}$ mice at 10 weeks of age. Scale bars 10μm.

L. Transcript levels of *mKi67, Havcr1 and Lcn2* in 10 weeks old *WT* (n=5) and *Acss2*$^{-/-}$ (n=6) mice at baseline.

Data are represented as mean ± SEM. P values determined by one-way ANOVA for D, E, G, I, J, L after Tukey's multiple comparison. *p < 0.05, **p < 0.01, ***p < 0.001 and ****p < 0.0001. Protein marker was cropped from all blots but was presented in full blots file.

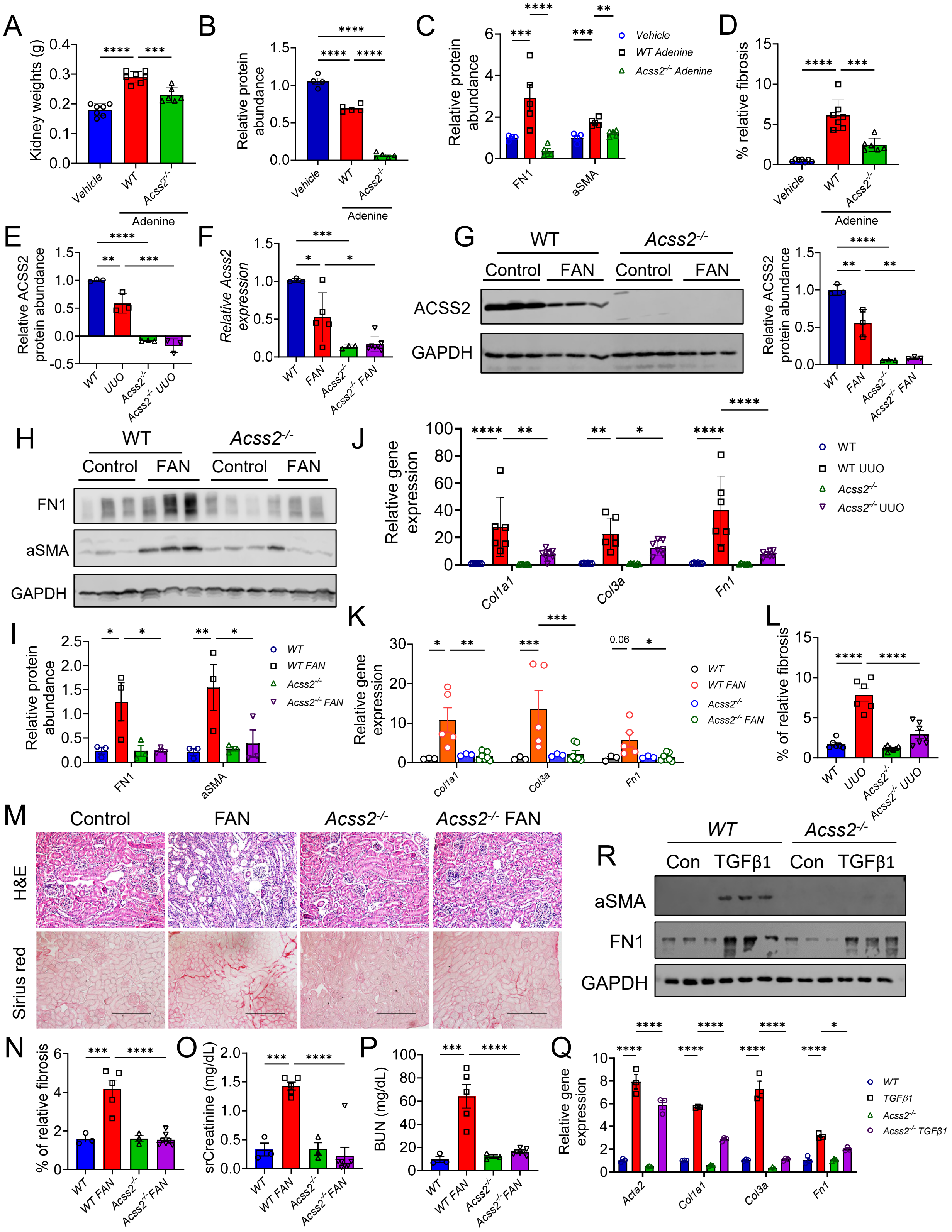

Supplemental Figure 2. Loss of ACSS2 protects from kidney disease.

A. Kidney weights in *WT* (n=7) and *Acss2*$^{-/-}$ (n=6) gavage with adenine.

B. Quantification of ACSS2 immunoblot in adenine kidneys by image J.

C. Quantification of FN1, aSMA and GAPDH immunoblots by image J.

D. Quantification of Sirius red images by image J and plotted as % relative fibrosis area in *WT* (n=7) and *Acss2*$^{-/-}$ (n=6) mice gavage with adenine.

E. Quantification of ACSS2 immunoblot in unilateral ureteral obstruction (UUO) kidneys by image J.

F. Transcript levels of *Acss2* in kidneys of *WT* (n=5) and *Acss2*$^{-/-}$ (n=7) mice injected with folic acid (FAN).

G. (Left) Immunoblot for ACSS2 in FAN kidneys of *WT* and *Acss2*$^{-/-}$ mice. (Right) Quantification of ACSS2 protein levels by image J.

H. Immunoblots of fibronectin (FN1) and alpha smooth muscle actin (aSMA) in whole kidney lysates of *WT* and *Acss2*$^{-/-}$ mice injected with folic acid.

I. Quantification of FN1, aSMA and GAPDH immunoblots by image J.

J. *Collagen 1a1 (Col1a1), collagen type 3a (Col3a) and fibronectin (Fn1)* mRNA levels measured in kidneys of *WT* (n=6) and *Acss2*$^{-/-}$ (n=7) mice in sham and UUO surgery.

K. *Col1a1, Col3a and Fn1* mRNA levels in kidneys of *WT* (n=5) and *Acss2*$^{-/-}$ (n=7) mice injected with folic acid.

L. Quantification of fibrosis by image J in UUO of WT (n=6) and *Acss2*$^{-/-}$ (n=7) mice kidneys.

M. H&E and Sirius red staining images of *WT* and *Acss2*$^{-/-}$ mice injected with folic acid. Scale bars 20μm.

N. Quantification of Sirius red images by image J and plotted as % relative fibrosis area in *WT* (n=5) and *Acss2*$^{-/-}$ (n=7) mice injected with folic acid.

O. Creatinine (sCr) levels estimated in serum samples collected from *WT* (n=5) and *Acss2*$^{-/-}$ (n=7) mice injected with folic acid.

P. Blood urea nitrogen (BUN) levels estimated in serum samples collected from *WT* (n=5) and *Acss2*$^{-/-}$ (n=7) mice injected with folic acid.

Q. *Acta2, Col1a1, Col3a and Fn1* mRNA levels were measured in tubular epithelial cells (TECs) of *WT* and *Acss2*$^{-/-}$ mice treated with TGFβ1.

R. Immunoblots for FN1, aSMA and GAPDH in TEC lysates of *WT* and *Acss2*$^{-/-}$ mice.

Data are represented as mean ± SEM. P values determined by one-way ANOVA. *p < 0.05, **p < 0.01, ***p < 0.001 and ****p < 0.0001. The data in F-P was a representative data of multiple experiments. Protein marker was cropped from all blots but was presented in full blots file.

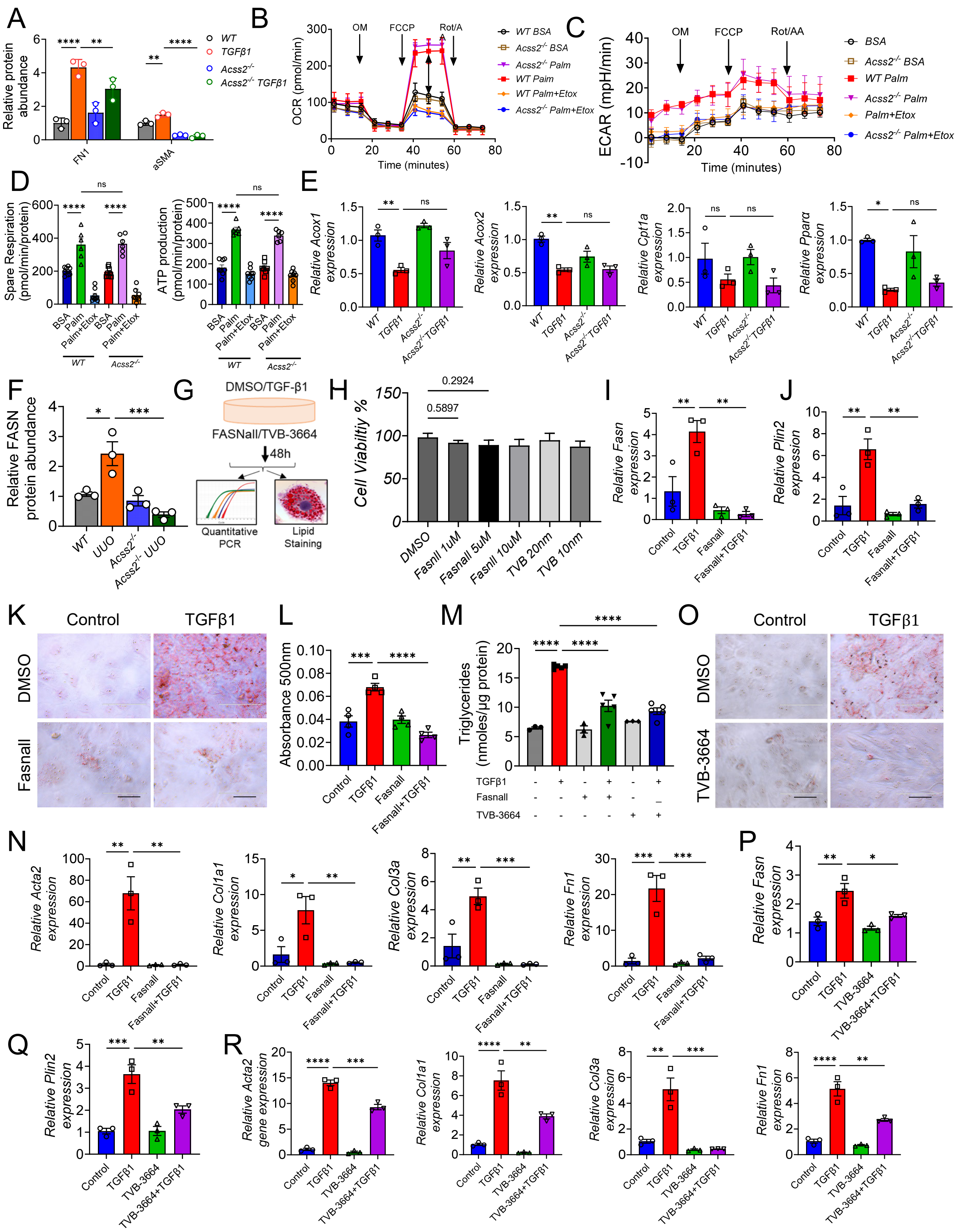

Supplemental Figure 3. TGFβ1 induces de novo lipogenesis in primary tubule cells.

A. Quantification of immunoblots of fibronectin (FN1), alpha smooth muscle actin (aSMA) and GAPDH protein by image J.
B. Seahorse-based oxygen consumption rate (OCR) in *WT* and *Acss2*$^{-/-}$ primary cells treated with palmitic acid.
C. Seahorse-based extracellular acidification rate (ECAR) in *WT* and *Acss2*$^{-/-}$ primary cells.
D. Spare respiration and ATP production by *WT* and *Acss2*$^{-/-}$ cells treated with palmitic acid.
E. Relative gene expression of *acyl CoA oxidase 1 (Acox1), Acox2, carnitine palmitoyl transferase 1 (Cpt1a)*, and peroxisome proliferator-activated receptor alpha (*Ppara)* in *WT* and *Acss2*$^{-/-}$ primary tubular epithelial cells (TECs) treated with TGFβ1.
F. Quantification of fatty acid synthase (FASN) and GAPDH immunoblots in kidneys of *WT* UUO and *Acss2*$^{-/-}$ UUO by image J.
G. Experimental scheme.
H. Cell viability of primary TECs treated with FASNall or TVB-3664.
I. Relative *Fasn* gene expression in primary TECs treated with TGFβ1 or FASNall.
J. Relative *perilipin 2* (*Plin2)* gene expression in primary TECs treated with TGFβ1 and FASNall.
K. Oil Red O staining in primary TECs treated with TGFβ1 or FASNall. Scale bars 20μM.
L. Quantification of Oil Red O staining.
M. Triglycerides in TECs treated with TGFβ1 and FASNall or TVB-3664.
N. Relative gene expression of *alpha smooth muscle actin (Acta2), collagen 1a1 (Col1a1), collagen type 3a (Col3a)* and *fibronectin (Fn1)* in primary TECs treated with FASNall or TGFβ1.
O. Oil Red O staining of primary TECs treated with TGFβ1 or TVB-3664. Scale bars 20μM.
P. Relative *Fasn* gene expression in primary TECs treated with TGFβ1 or TVB-3664.
Q. Relative *Plin2* gene expression in primary TECs treated with TGFβ1 or TVB-3664.
R. Relative gene expression of *Acta2, Col1a1, Col3a* and *Fn1* in TECs treated with TGFβ1 or TVB-3664.

All graphs present means of ± SEM. P values determined by one-way ANOVA for panels A, and D-R. *$p < 0.05$, **$p < 0.01$, ***$p < 0.001$ and ****$p < 0.0001$. The data was a representative of multiple experiments.

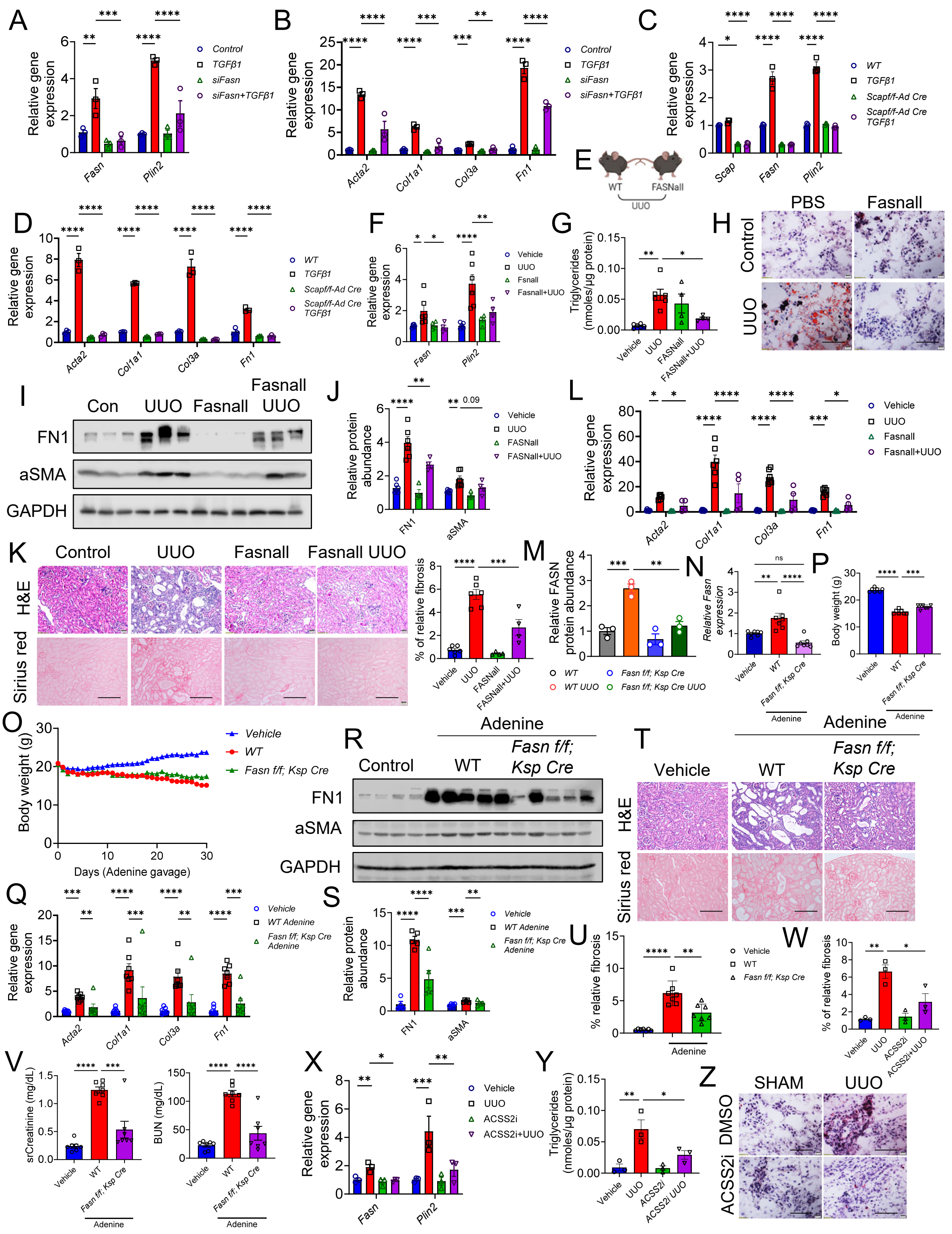

Supplemental Figure 4. Inhibition of *de novo* lipogenesis prevents kidney fibrosis.

A. Gene expression level of fatty acid synthase (*Fasn)* and perilipin 2 (*Plin2)* in TECs transfected with small interfering RNA against *Fasn* (*siFasn)* and treated with TGFβ1 for 48h.

B. Gene expression levels of *alpha smooth muscle actin (Acta2), Collagen 1a1 (Col1a1), collagen type 3a (Col3a)* and *fibronectin (Fn1)* in TECs transfected with *siFasn* and treated with TGFβ1.

C. Gene expression level of *sterol regulatory element binding protein (SREBP) cleavage activating protein* (*Scap), Fasn,* and *Plin2* were measured in *WT* and *Scapf/f* TECs treated with Adeno-Cre virus (Ad-Cre) for 24h and treated with TGFβ1 for 48h.

D. Gene expression level of *Acta2, Col1a1, Col3a* and *Fn1* measured in *WT* and *Scapf/f* TECs treated with Ad-Cre and TGFβ1. WT and TGF β1 treated samples are the same as used in supplemental figure 2 panel O.

E. Experimental design.

F. Gene expression level of *Fasn* and *Plin2* in kidneys of mice injected with FASNall or PBS followed by UUO injury.

G. Kidney triglyceride levels in mice injected with FASNall or PBS and subjected to UUO injury.

H. Oil Red O-stained kidney sections of mice injected with FASNall, or PBS followed by UUO injury. Scale bars 10μm.

I. FN1, aSMA and GAPDH immunoblots in kidneys of mice injected with FASNall or PBS followed by UUO injury.

J. Quantification of immunoblots of aSMA and FN1 proteins in whole kidney lysates of UUO and FASNall treated UUO mice in image J.

K. (Left) H&E and Sirius Red staining in kidney sections of mice injected with FASNall or PBS in followed by UUO injury. Scale bars 20μm. (Right) Percentage of fibrosis quantified in image J.

L. Gene expression level of *Acta2, Col1a1, Col3a* and *Fn1* in kidneys of mice injected with FASNall or PBS in followed by UUO injury.

M. Quantification of FASN and GAPDH immunoblots of *Fasn f/f; Ksp Cre* mice with or without UUO.

N. *Fasn* gene expression levels in *Fasn f/f; Ksp Cre* (n=7) and *WT* (n=7) mice treated with adenine.

O. Daily body weights in adenine-CKD model of *Fasn f/f; Ksp Cre* (n=7) and *WT* (n=7) mice.

P. Final body weights of *Fasn f/f; Ksp Cre* (n=7) and *WT* (n=7) mice treated with adenine.

Q. Gene expression level of *Acta2, Col1a1, Col3a* and *Fn1* in *WT (n=7)* and *Fasn f/f; Ksp Cre (n=7)* mice gavage with adenine.

R. FN1, aSMA and GAPDH immunoblots in kidneys of mice gavage with adenine in *WT* (n=5) and *Fasn f/f; Ksp Cre* (n=5).

S. Quantification of FN1, aSMA and GAPDH immunoblots of *Fasn f/f; Ksp Cre and WT* mice gavage with adenine.

T. H&E and Sirius Red staining in kidney sections of mice treated with adenine or vehicle. Scale bars 20μm.

U. Quantification of Sirius red staining in image J in kidneys of mice gavaged with adenine in *WT* and *Fasn f/f; Ksp Cre* mice.

V. Serum creatinine (sCr) and blood urea nitrogen (BUN) estimated in *WT* (n=7) and *Fasn f/f; Ksp Cre* (n=7) mice gavage with adenine.

W. Quantification of Sirius red staining in kidneys mice with UUO or ACSS2i treatment.

X. Gene expression levels of *Fasn* and *Plin2* in *WT* (n=3) and ACSS2i (n=3) injected control and UUO kidneys.

Y. Kidney triglycerides n kidneys of *WT* (n=3) and ACSS2i (n=3) injected control and UUO kidneys.

Z. Oil Red O staining in fresh kidney sections of *WT* and ACSS2i injected control and UUO mice. Scale bars 10μm.

Data are represented as mean ± SEM. P values determined by one-way ANOVA after Tukey's multiple comparison. *p < 0.05, **p < 0.01, ***p < 0.001 and ****p < 0.0001. WT and TGFβ1 data in panel D was the same as used in data panel Q of supplementary figure 2. Controls and WT adenine samples in panels N-V are the same for *Acss2*$^{-/-}$ and *Fasn f/f; Ksp Cre* adenine experiments. Protein marker was cropped from all blots but was presented in full blots file.

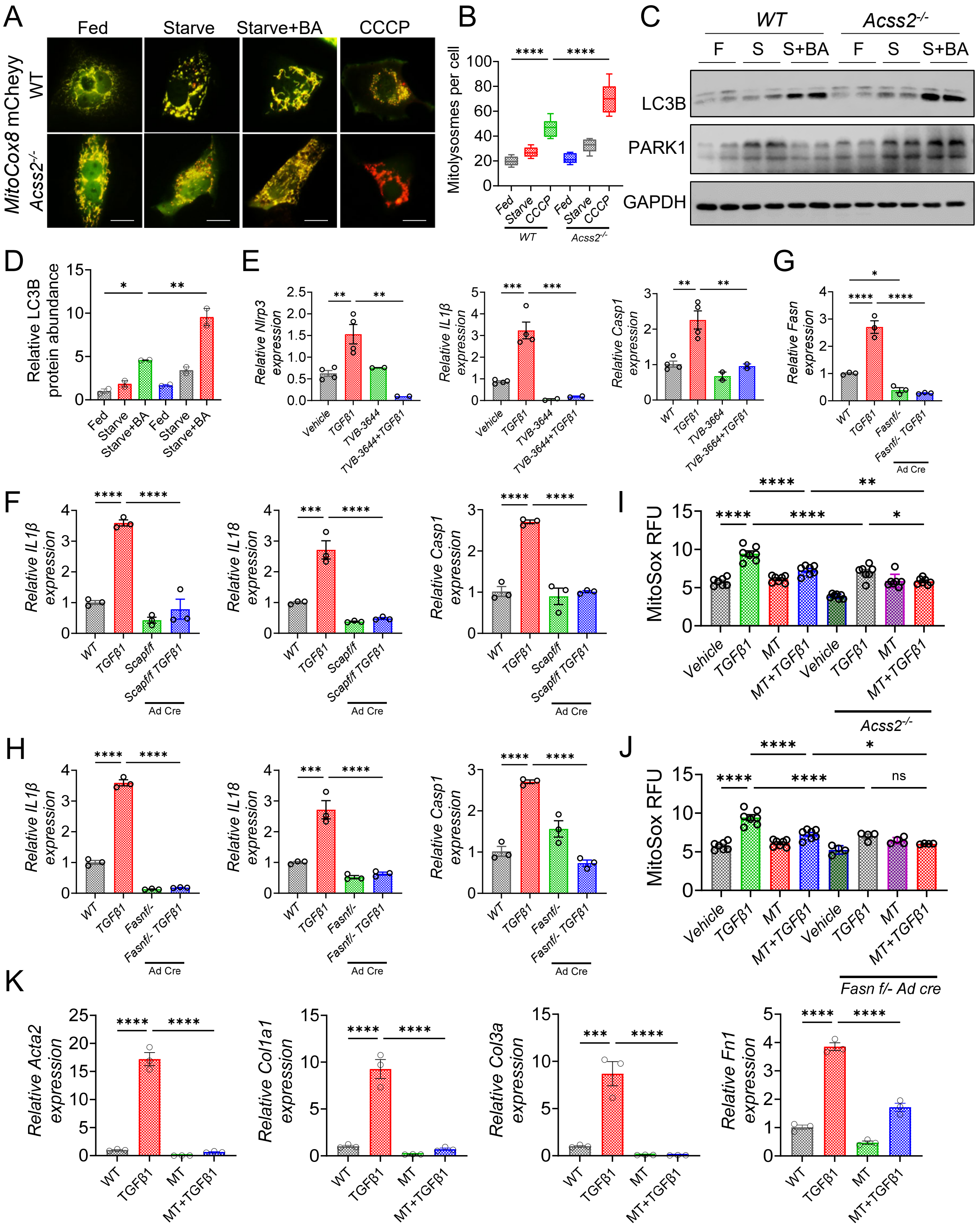

Supplemental Figure 5. Suppression of mitochondrial ROS suppresses NLRP3-inflammasome activation in primary tubular cells.

A. Mitophagy was assessed in primary tubular epithelial cells (TECs) transfected with mitoCox8 eGFP-mCherry plasmid and subjected to various mitophagy inducers for 2h. Scale bars 10µm.

B. Mitolysosomes quantified in Image J.

C. Immunoblots of LC3B and Parkin1 in primary TECs in fed, starve, and bafilomycin (BA).

D. Quantification of immunoblots of LC3B in Image J.

E. Relative gene expression of *Nlrp3, IL1B*, and caspase1 (*Casp1)* in TECs treated with TVB-3664 or TGFβ1 for 48hr.

F. Relative gene expression of *IL1B, IL18* and *Casp1* in *WT* and *Scapf/f* TECs transfected with Adeno-Cre virus (Ad-Cre) and treated with TGFβ1.

G. Relative gene expression of *Fasn* in *WT* and *Fasnf/-* TECs transfected with Ad-Cre and treated with TGFβ1. WT and TGF β1 samples are same used in the supplementary figure 4 panel C.

H. Relative gene expression of *IL1B, IL18* and *Casp1 WT* and *Fasnf/-* TECs transfected with Ad-Cre and treated with TGFβ1.

I. Relative fluorescence of MitoSox quantified in *WT* and *Acss2*$^{-/-}$ cells treated with vehicle or TGFβ1 or mitoTempo (MT).

J. Relative fluorescence of MitoSox quantified in *WT* and *Fasnf/-* cells transfected with Ad Cre for 24h and treated with vehicle or TGFβ1 or MT.

K. Relative gene expression of *alpha smooth muscle actin (Acta2), Collagen 1a1 (Col1a1), collagen type 3a (Col3a)* and *fibronectin (Fn1)* measured in primary TECs treated with TGFβ1 or MT.

Data are represented as mean ± SEM. P values determined by one-way ANOVA after Tukey's multiple comparison. *p < 0.05, **p < 0.01, ***p < 0.001 and ****p < 0.0001. Vehicle, TGFβ1, MT, and MT+TGFβ1 data are the same for both panels I and J. The data was a representative of multiple experiments. Protein marker was cropped from all blots but was presented in full blots file.

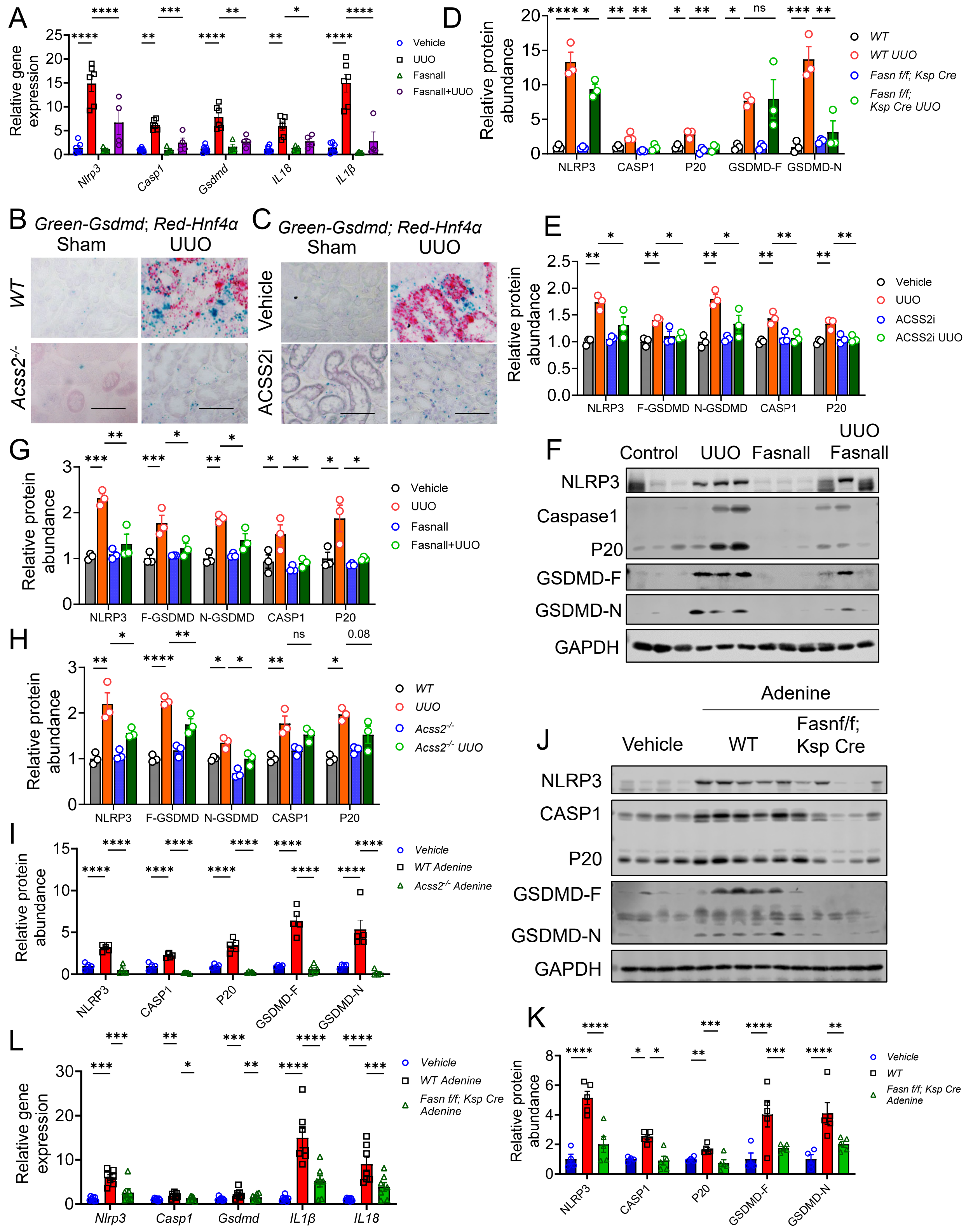

Supplemental Figure 6. Inhibition of de novo lipogenesis suppresses ROS-induced NLRP3 inflammasome activation.

A. Gene expression levels of *Nlrp3*, caspase1 (*Casp1), Gasdermin D (Gsdmd), IL1B,* and *IL18* measured in kidneys of mice injected with vehicle (n=6) or FASNall (n=4).

B. In situ hybridization of mouse *Gsdmd* in kidneys of *WT* and *Acss2*$^{-/-}$ mice with unilateral ureteral obstruction (UUO) (upper panel) and its quantification (lower panel). HNF-4A was used to detect proximal tubule (PT) cells. Scale bars 10μm.

C. In situ hybridization of mouse *Gsdmd* in kidneys of mice injected with ACSS2i or UUO injury (upper panel) and its quantification (lower panel). HNF-4A was used to detect proximal tubule (PT) cells. Scale bars 10μm.

D. Quantification of immunoblots of NLRP3, CASP1, P20 (Cleaved-CASPASE1), GSDMD-F (GSDMD-Full length), GSDMD-N (cleaved-GSDMD) and GAPDH in control and UUO kidneys of WT and *Fasn f/f; Ksp Cre* mice.

E. Quantification of immunoblots performed for NLRP3, total and p20 forms of Caspase1 and full length GSDMD and N-GSDMD in WT UUO and ACSS2i injected UUO mice.

F. Immunoblots of NLRP3, CASP1, P20, GSDMD-F, GSDMD-N and GAPDH in kidney lysates of mice injected with vehicle (n=6) or FASNall (n=4).

G. Quantification of immunoblots of NLRP3, CASP1, P20, GSDMD-F, GSDMD-N and GAPDH in vehicle or FASNall injected mice with UUO.

H. Quantification of immunoblots performed for NLRP3, total and P20 forms of CASP1 and GSDMD-F and GSDMD-N in *WT* UUO and *Acss2*$^{-/-}$ mice subjected to UUO.

I. Quantification of immunoblots of NLRP3, CASP1, P20, GSDMD-F, GSDMD-N and GAPDH in vehicle *(n=4), WT (n=5)* and *Acss2*$^{-/-}$ *(n=5)* mice gavage with adenine.

J. Immunoblots of NLRP3, CASP1, P20, GSDMD-F, GSDMD-N and GAPDH in vehicle (n=4), WT *(n=5)* and *Fasn f/f; Ksp Cre (n=5)* mice gavage with adenine.

K. Quantification of immunoblots of NLRP3, CASP1, P20, GSDMD-F, GSDMD-N and GAPDH in vehicle (n=4), WT *(n=5)* and *Fasn f/f; Ksp Cre (n=5)* mice gavage with adenine.

L. Gene expression of levels of *Nlrp3*, *Casp1, (Gsdmd), IL1β,* and *IL18* measured in kidneys of mice injected with vehicle (n=7), or adenine gavage in *WT (n=7)* and *Fasn f/f; Ksp Cre (n=7)* mice.

Data are represented as mean ± SEM. P values determined by one-way ANOVA after Tukey's multiple comparison. *p < 0.05, **p < 0.01, ***p < 0.001 and ****p < 0.0001. Controls and WT adenine samples are the same for both *Acss2*$^{-/-}$ and *Fasn f/f; Ksp Cre* adenine experiment. Protein marker was cropped from all blots but was presented in full blots file.

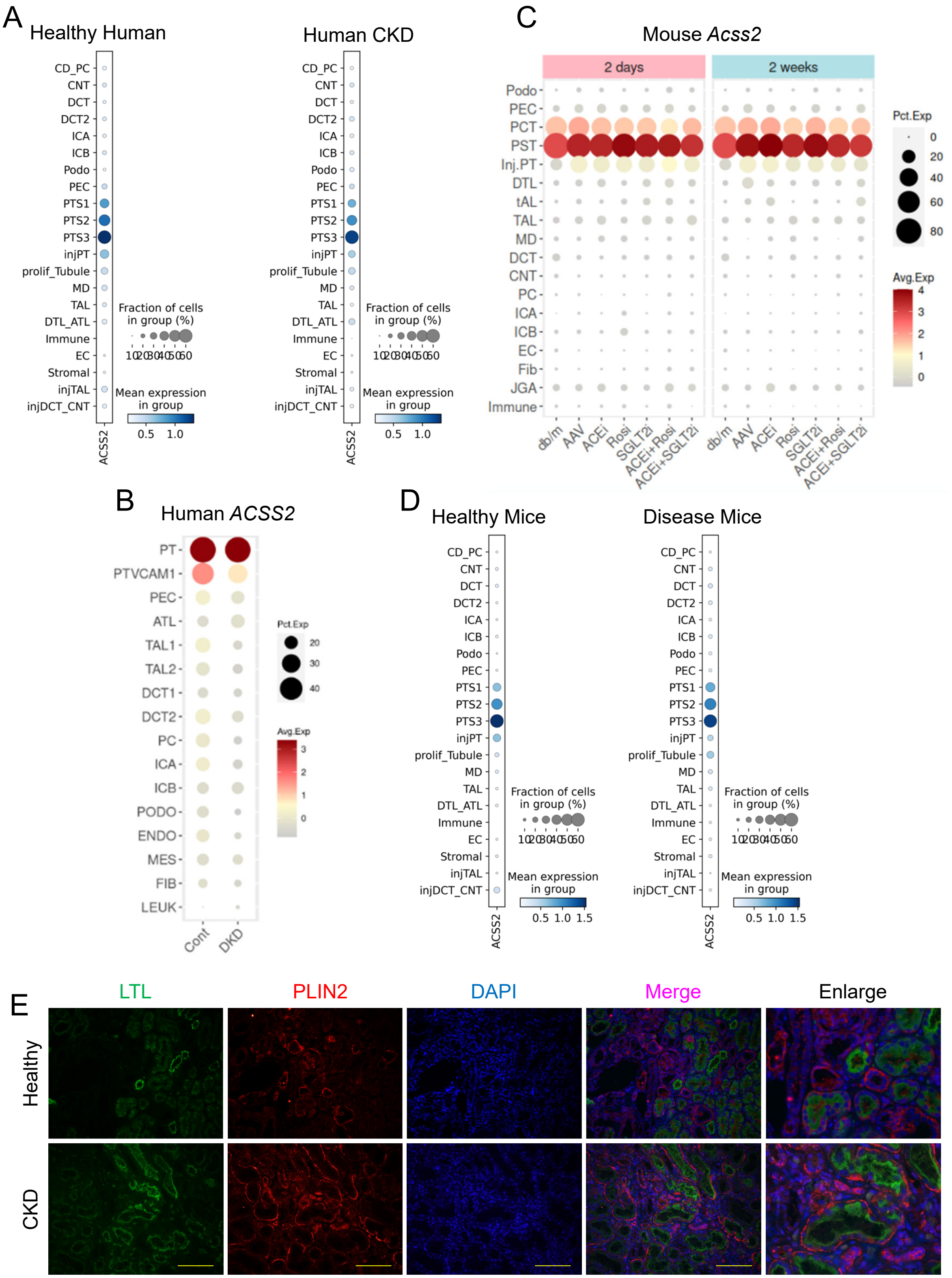

566

567 **Supplemental Figure 7. Fatty acid synthesis correlated with fibrosis in CKD patients.**

A. ACSS2 gene expression in one million human snRNA-seq atlas from healthy (n=141505 cells) and chronic kidney disease patients (n=157897 cells). The size of the bubble correlates with the percent of positive cells, and the color indicates the level of its expression (darker higher). Cell types: collecting duct-principal cells (CD_PC), distal convoluted tubule (DCT 1 and 2), intercalated cells A (IC_A) and intercalated cells B (IC_B), podocytes (Podo), parietal epithelial cells (PEC), proximal tubule (PT), injured PT (injPT), proliferative tubule (prolif_Tubule), macula densa (MD), thick ascending Loop of Henle (TAL), distal thin limb-ascending thin limb (DTL-ATL), immune cells (immune), endothelial cells (Endo), stromal cells (stroma), inured TAL (injTAL), and injured DCT (injDCT_CNT).

B. ACSS2 gene expression in human snRNA-seq data (n=39,176 cells) from healthy and diabetic kidney disease patients. The size of the bubble correlates with the percent of positive cells, and the color indicates the level of the expression (darker higher). Cell types: VCAM1 positive PT cells (PT-VCAM1+), mesangial (Mes), fibroblasts (Fibro), leukocytes (LEUK).

C. ACSS2 gene expression in one million mouse kidney single cell RNA-seq (scRNA-seq) atlas. The size of the dot correlates with the percent positive cells, and the color indicates the level of the expression (darker higher). Cell type annotations are the same as in panel B. Juxtaglomerular apparatus (JGA), and immune cells (immune).

D. ACSS2 gene expression in mouse kidney single cell and snRNA-seq atlas (n=382551 control cells, and n=64948 diseased cells). The size of the dot correlates with the percent positive cells, and the color indicates the level of the expression (darker higher). Cell type annotations are the same as in panel A.

E. Immunofluorescence of PLIN2 in healthy (upper panel) and CKD (lower panel) kidneys of human subjects. LTL was used to stain PT segments of the kidney. Scale bars 20µm and enlarge scale is 10µm.

Supplementary Table 1: Gene prioritization table.

This table data is related to Figure 1, E, F, and G, comprising all SNPs that are in this genomic region including genetic evidence, SNP position, target genes and genetic tests.

Supplementary Table 2: Prioritized SNPs.

This table data is related to Figure 1G comprises six prioritized SNPs in establishing ACSS2 causal risk for this locus.

Supplementary Table 3. Guide RNAs and human primers.

This table is related to Figure 1I and Supplementary Figure 1, D and E. Includes information on ACSS2 guide RNAs, genotyping primers, and human qPCR primers for the three genes.

Supplementary Table 4: Mouse gene primers.

Mouse primers were used in this study.

S.No	Gene	Forward primer	Reverse primer
1	*Acss2*	GCTTCTTTCCCATTCTTCGGT	CCCGGACTCATTCAGGATTG
2	*Col1a1*	TGCCTGGACCTCCTGGCGAGCGT	AGCAGGTCCGGGAGCACCACGTT
3	*Col3a*	ACAGCTGGTGAACCTGGAAG	ACCAGGAGATCCATCTCGAC
4	*Fn1*	ACAAGGTTCGGGAAGAGGTT	CCGTGTAAGGGTCAAAGCAT
5	*Acta2*	GTTCAGTGGTGCCTCTGTCA	ACTGGGACGACATGGAAAAG
6	*Acox1*	CTTGGATGGTAGTCCGGAGA	TGGCTTCGAGTGAGGAAGTT
7	*Acox2*	TACCAACGCCTGTTTGAGTG	TTTCCAGCTTTGCATCAGTG
8	*Pparα*	CGAGAAGGAGAAGCTGTTGG	TCAGCGGGAAGGACTTTATG
9	*Hmgcr*	CGTAAGCGCAGTTCCTTCC	TTGTAGCCTCACAGTCCTTGG
10	*Hmgcs1*	GGTCTGATCCCCTTTGGTG	TGTGAAGGACAGAGAACTGTGG
11	*Fdps*	TCTTTCTACCTGCCTATTGCG	CTCCAAAGAGATCAAGGTAGTCG

12	*Scap*	AAGATTTCTGTGCCAGGGAG	CTGTGAAGGGTTACTCGCC
13	*Srebp1*	GGCATGAAACCCGAAGTGGT	AGAGGGAGTGAGAATGCCCC
14	*Fasn*	GCCAACTCGAGGGACACATC	GGGCTTCACGACTCCATCAC
15	*Acaca*	GCCTGAGACTGGATCAGTGG	TGTGTGACTGGGCTGTGTGA
16	*Plin2*	GGATAAGCTCTATGTCTCGTGG	GTCTGGCATGTAGTCTGGAG
17	*Cpt1a*	GGTCTTCTCGGGTCGAAAGC	TCCTCCCACCAGTCACTCAC
18	*Nlrp3*	ATTACCCGCCCGAGAAAGG	TCGCAGCAAAGATCCACACAG
19	*Il-1β*	GCAACTGTTCCTGAACTCAACT	ATCTTTTGGGGTCCGTCAACT
20	*Il18*	ACTGTACAACCGCAGTAATAC	AGTGAACATTACAGATTTATCCC
21	*Caspase1*	ACAAGGCACGGGACCTATG	TCCCAGTCAGTCCTGGAAATG
22	*Gasdermin D*	CCATCGGCCTTTGAGAAAGTG	ACACATGAATAACGGGGTTTCC
23	*Gapdh*	AGGTCGGTGTGAACGGATTTG	TGTAGACCATGTAGTTGAGGTCA
