# Supplementary material for: ACSS2 gene variants determine kidney disease risk by controlling de novo lipogenesis in kidney tubules
Source: J Clin Invest. 2023 Dec 5;134(4):e172963. doi: 10.1172/JCI172963 (PMC10866669; doi:10.1172/JCI172963)

Full unedited blots for Figure 2D

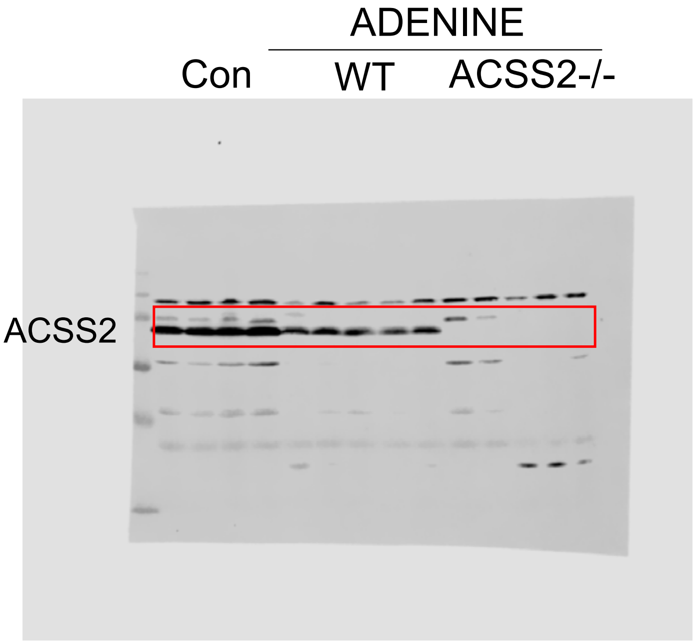

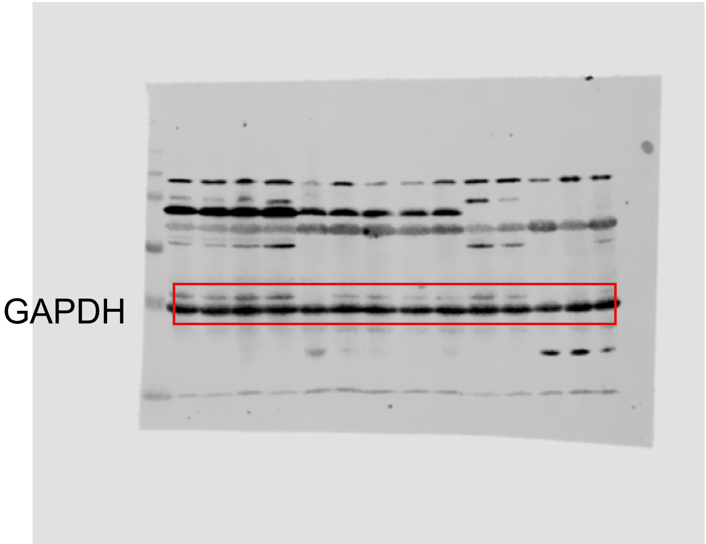

Full unedited blots for Figure 2F

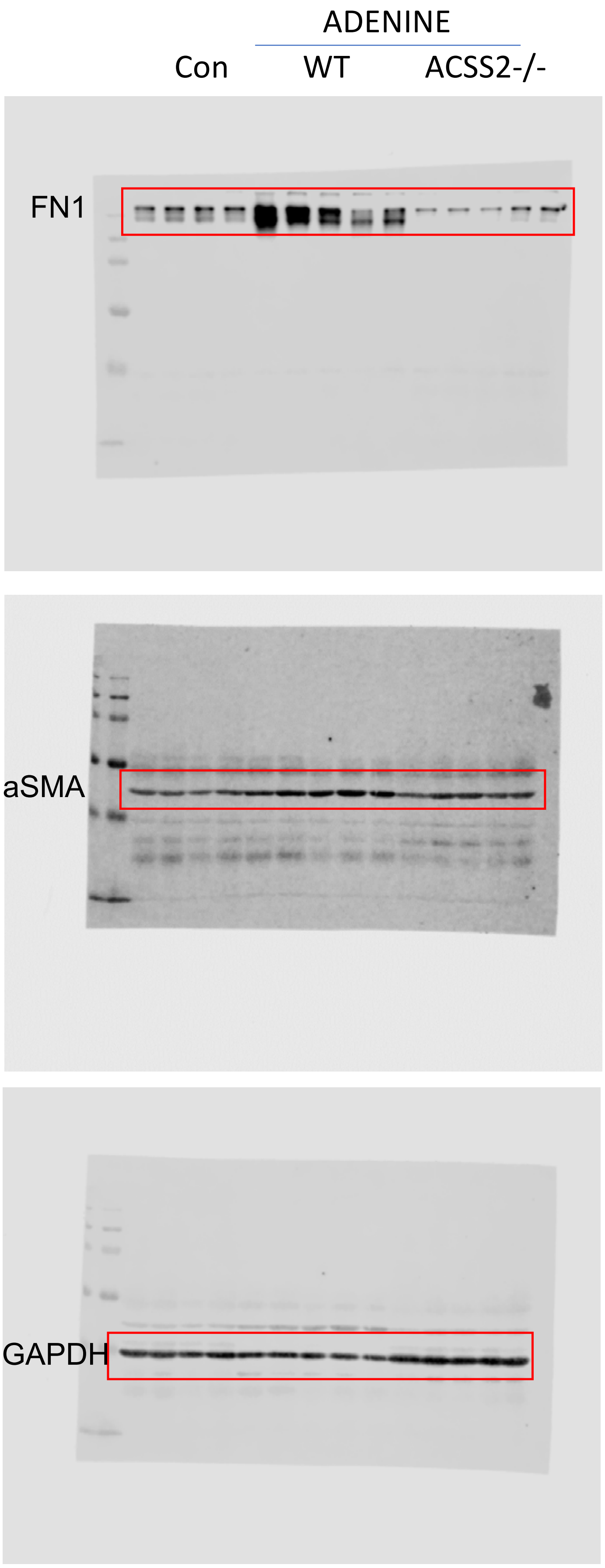

Full unedited blots for Figure 2K

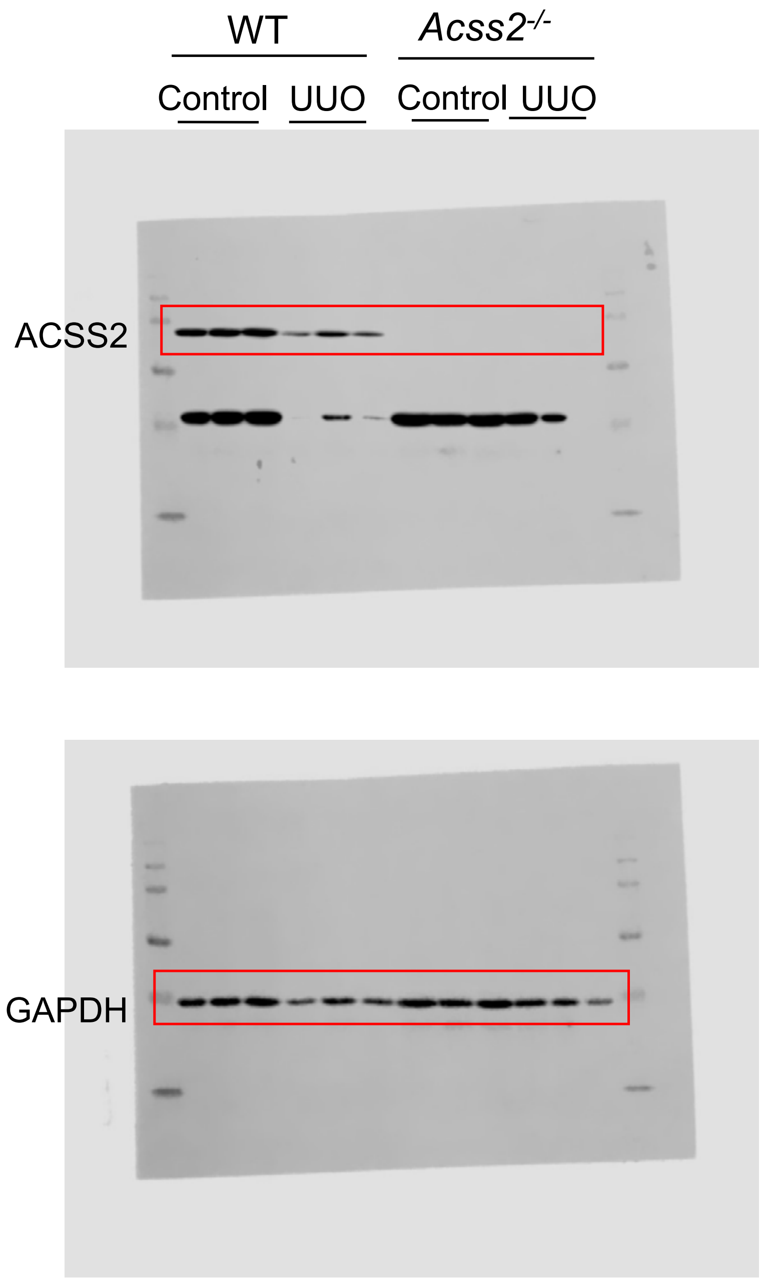

Full unedited blots for Figure 2L

WT | *Acss2*$^{-/-}$

Control UUO Control UUO

FN1

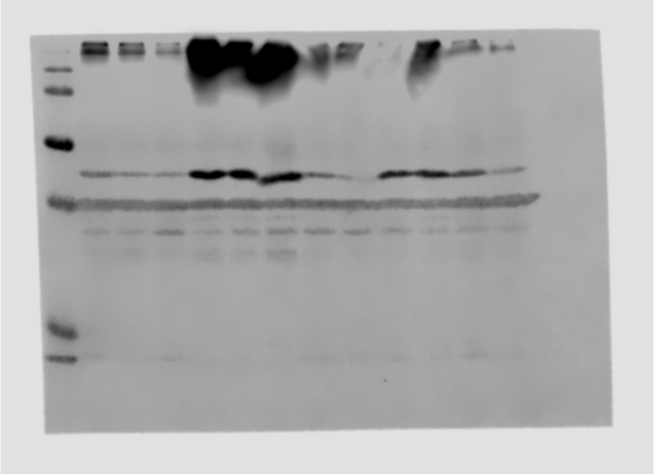

aSMA

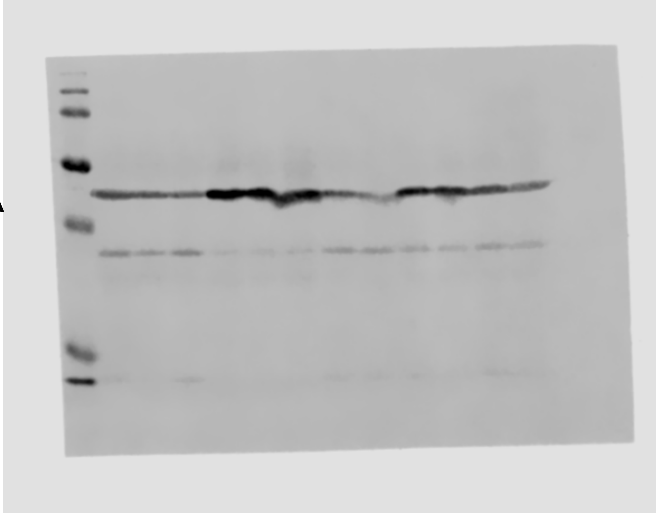

GAPDH

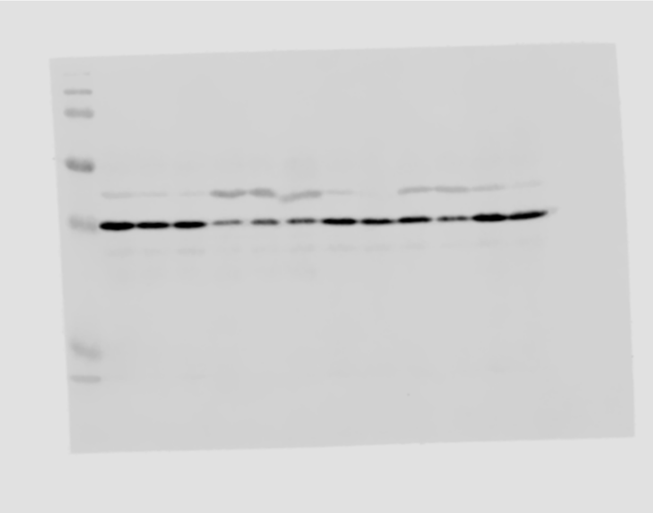

Full unedited blots for Figure 3B

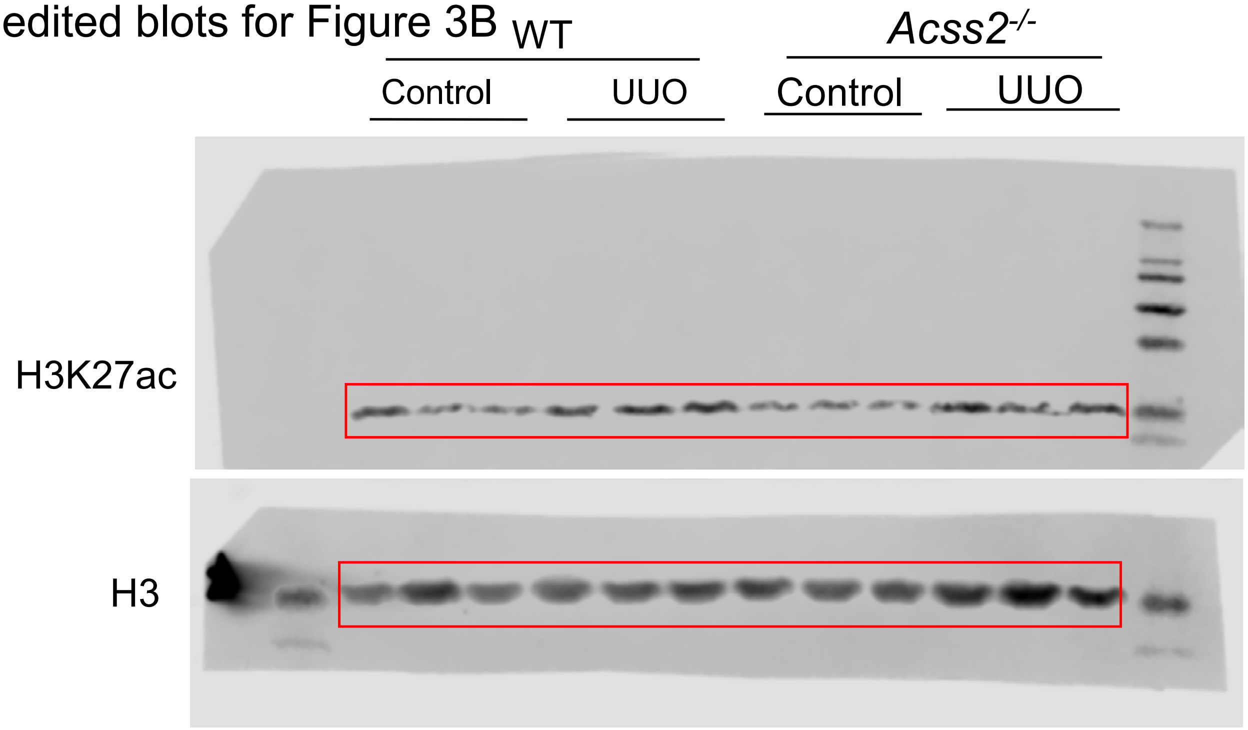

Full unedited blots for Figure 3J

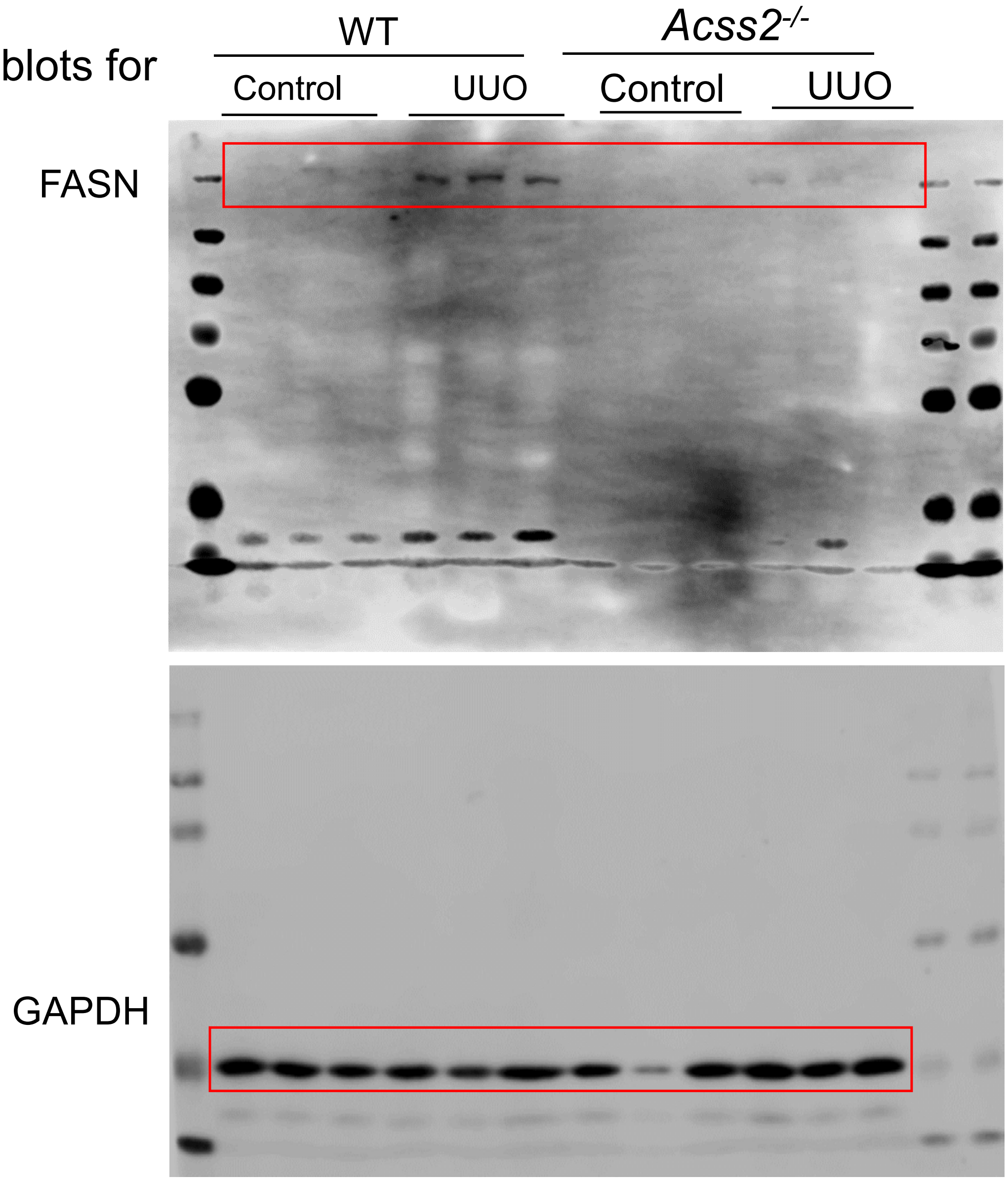

Full unedited blots for Figure 4C

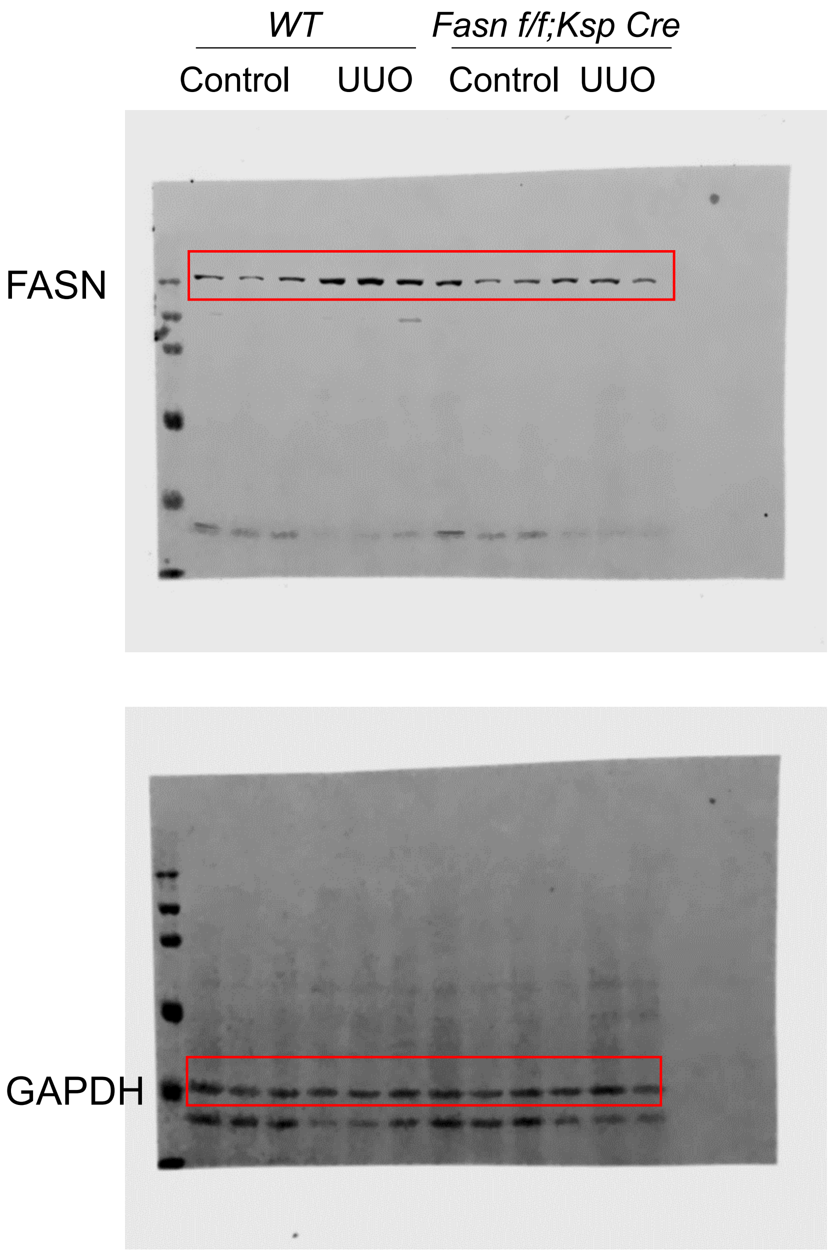

Full unedited blots for Figure 4E

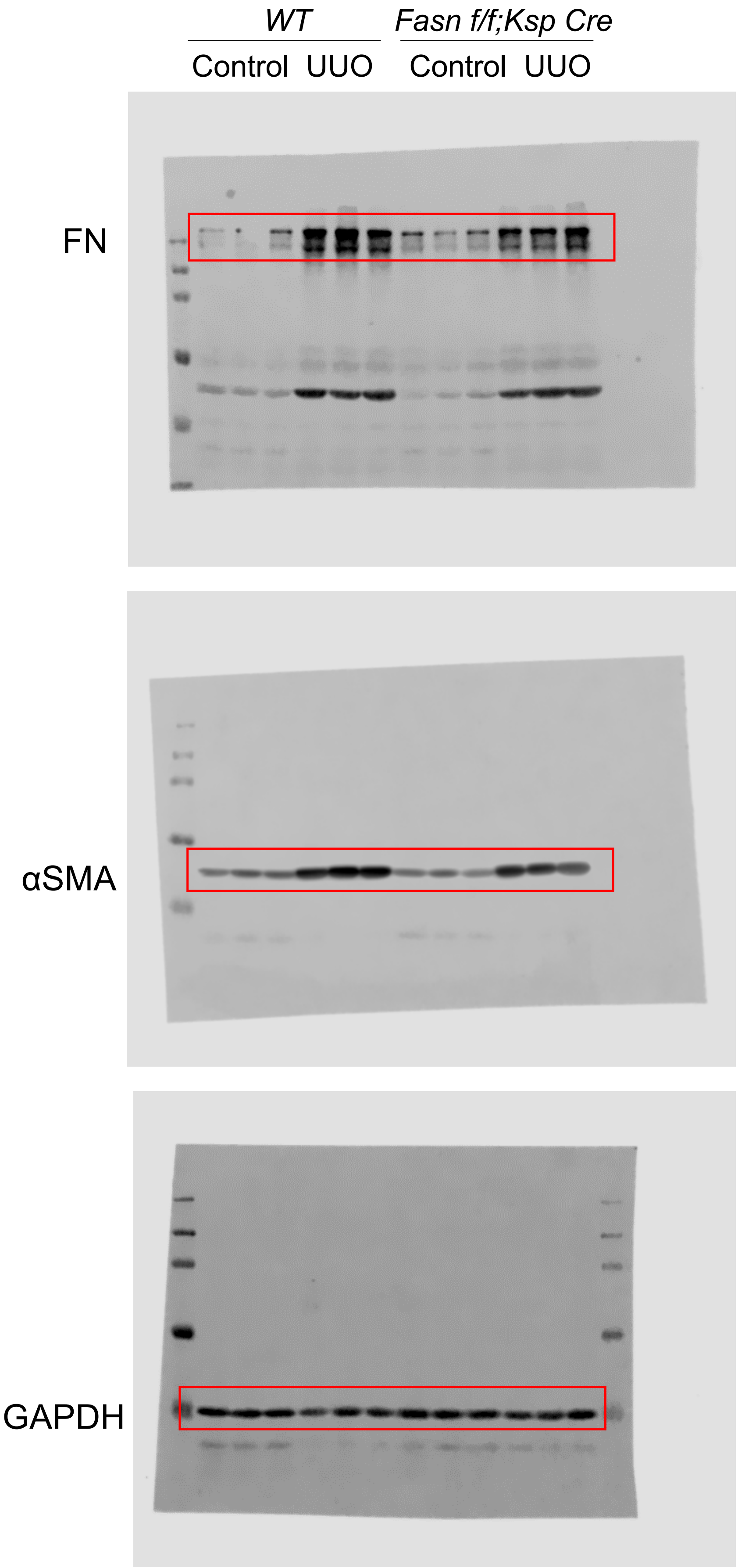

Full unedited blots for Figure 4J

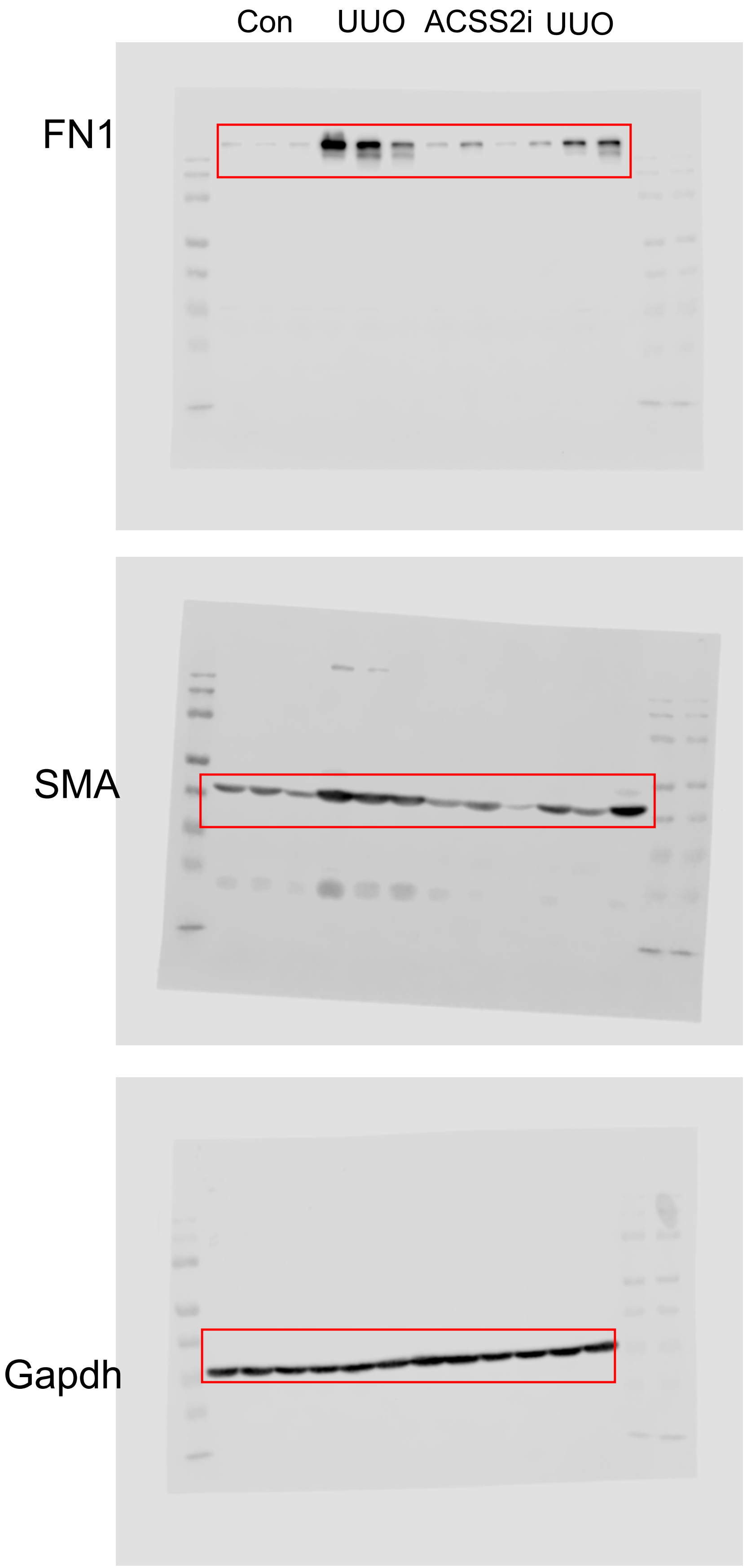

Full unedited blots for Figure 6B

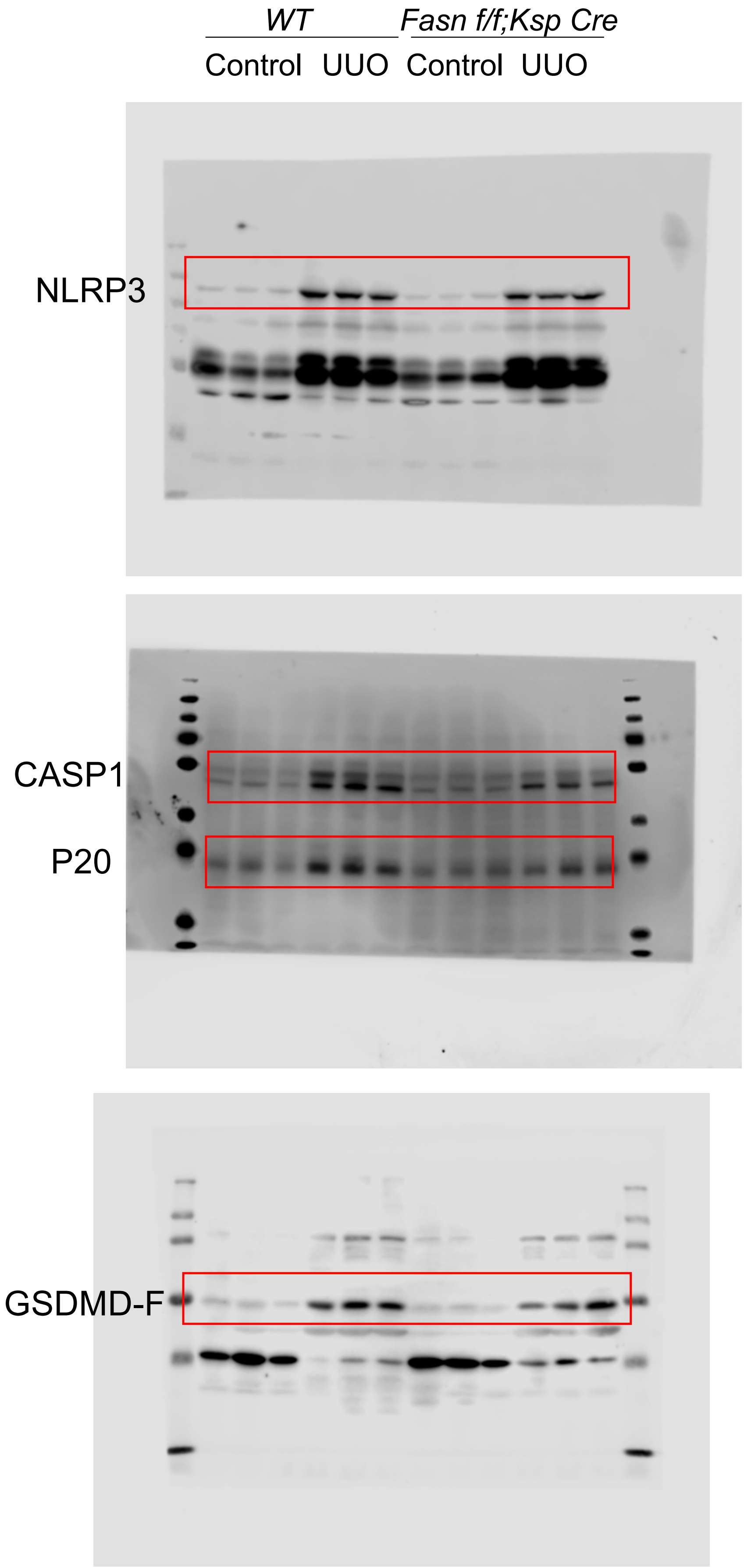

Full unedited blots for Figure 6B

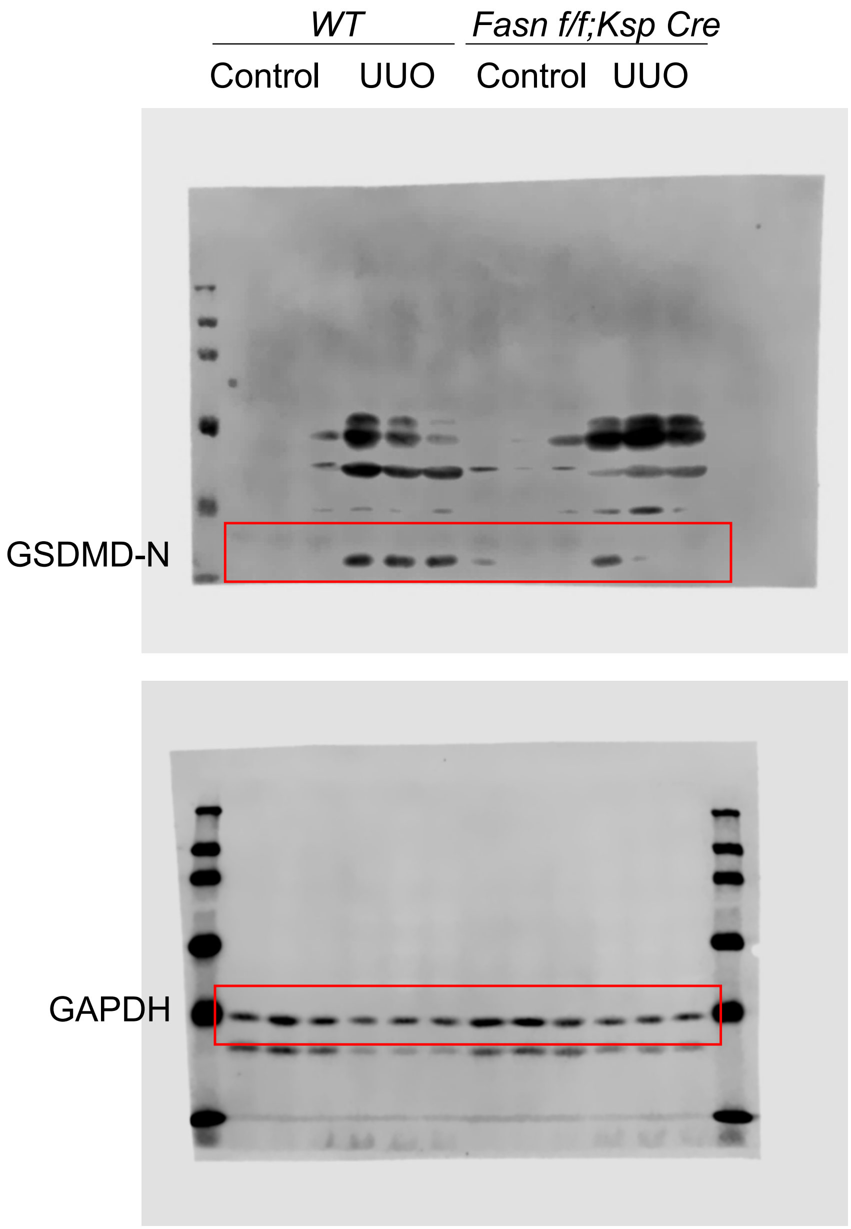

Full unedited blots for Figure 6E

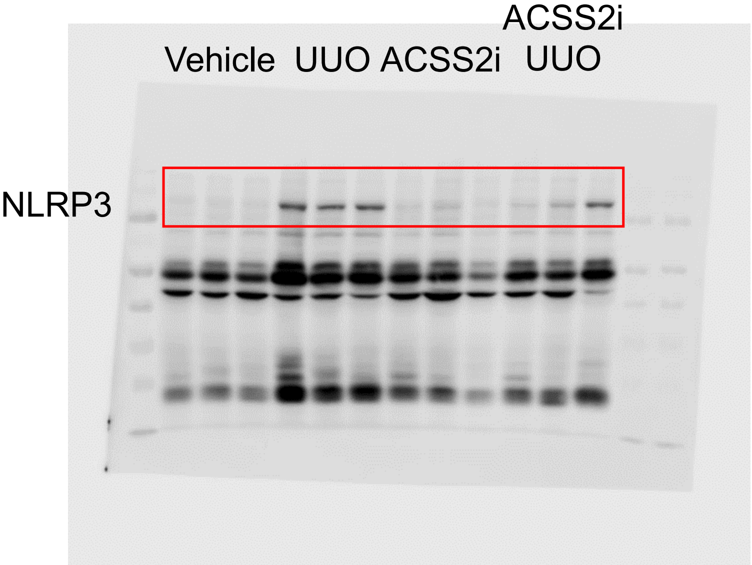

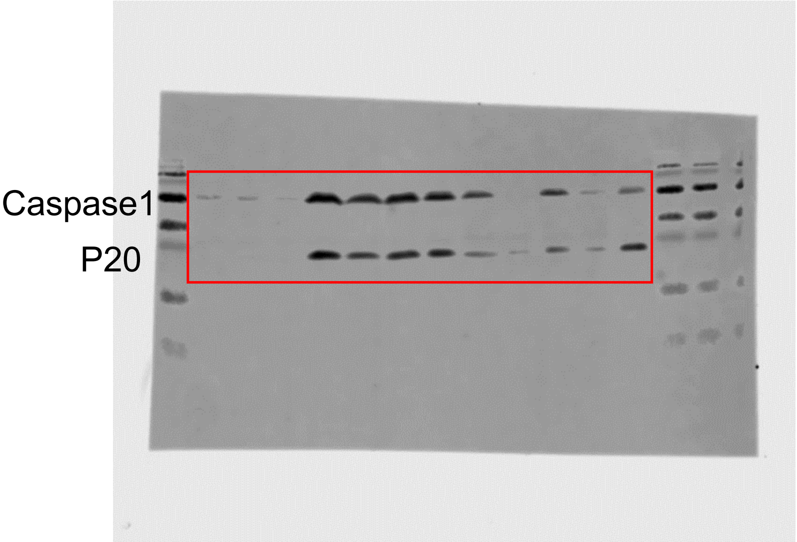

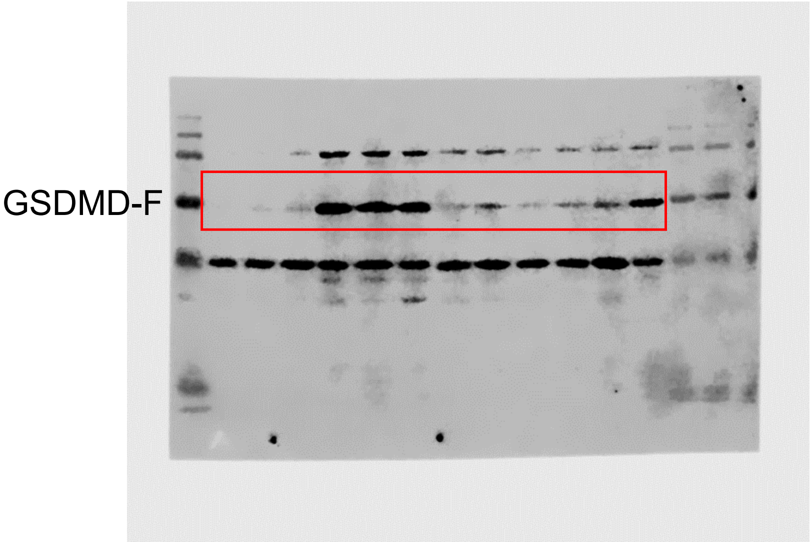

Full unedited blots for Figure 6E

Vehicle UUO ACSS2i ACSS2i UUO

GSDMD-N

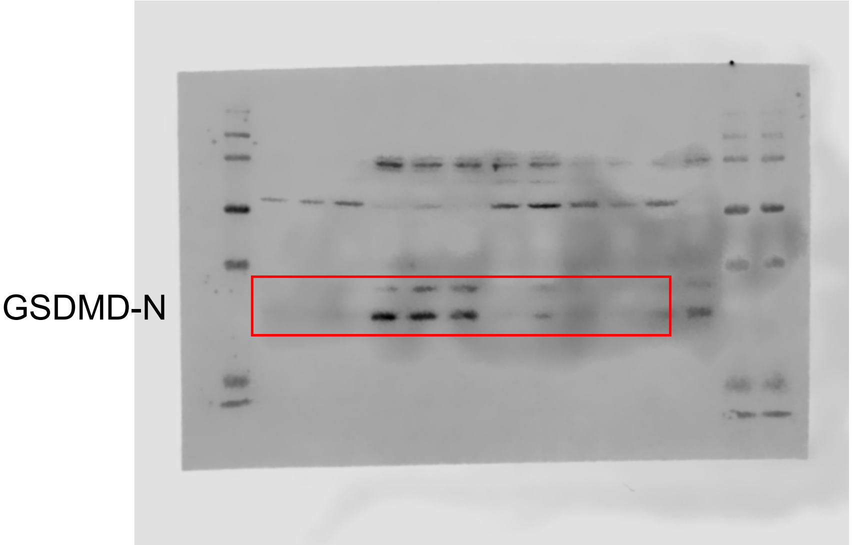

GAPDH

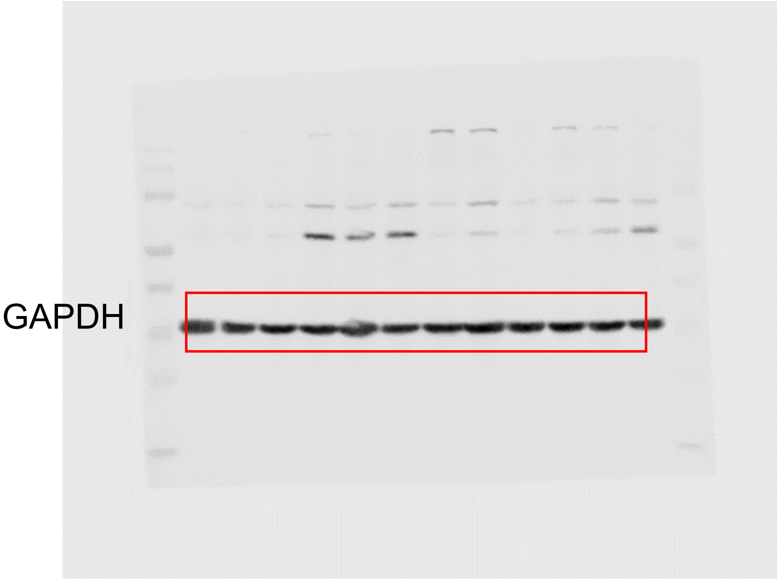

Full unedited blots for Figure 6H

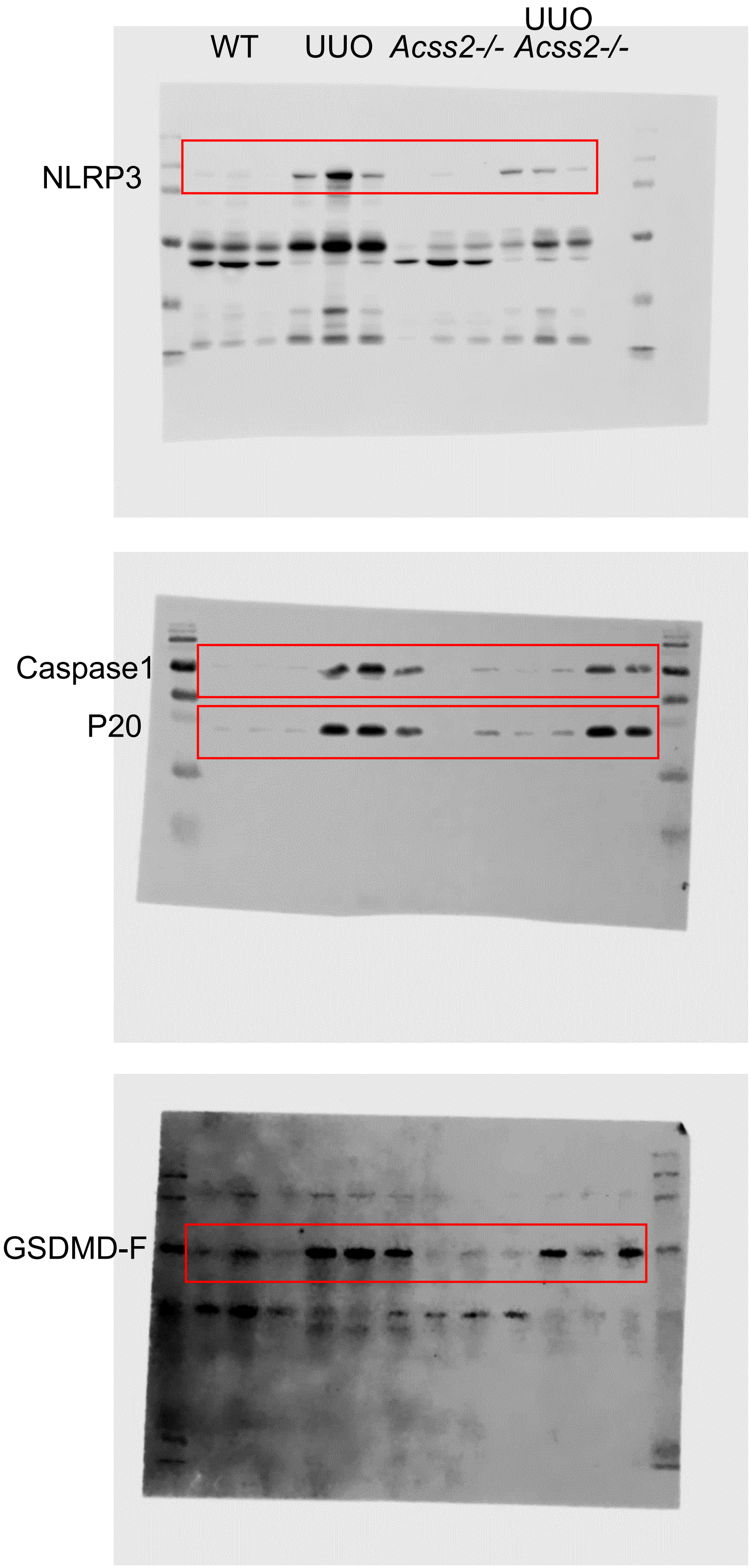

Full unedited blots for Figure 6H

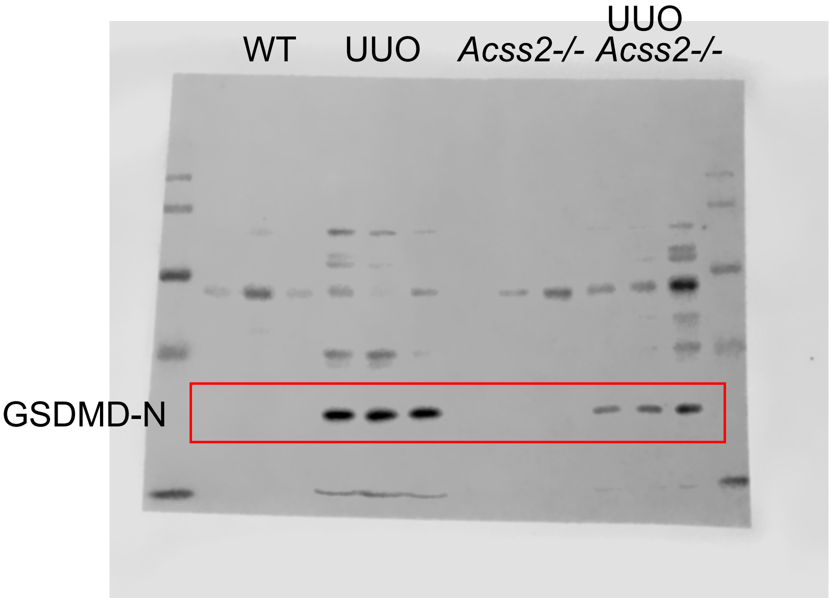

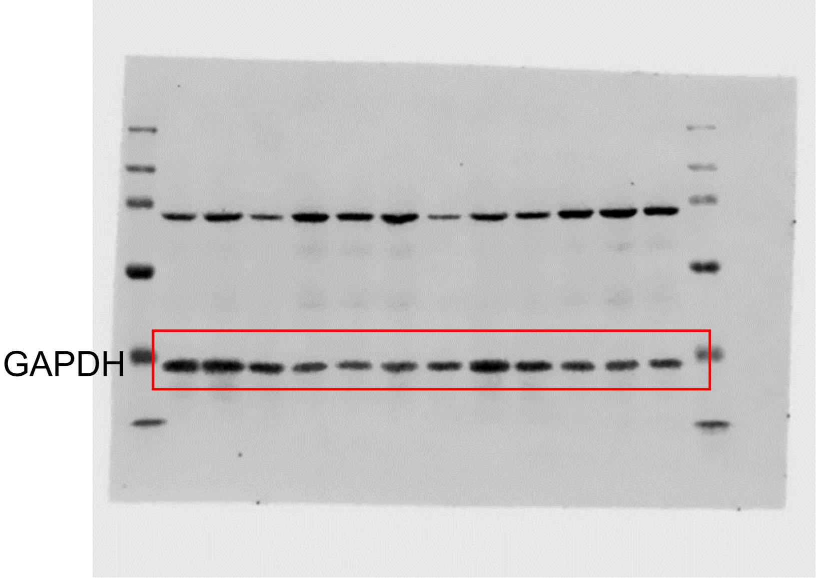

Full unedited blots for Figure 6J

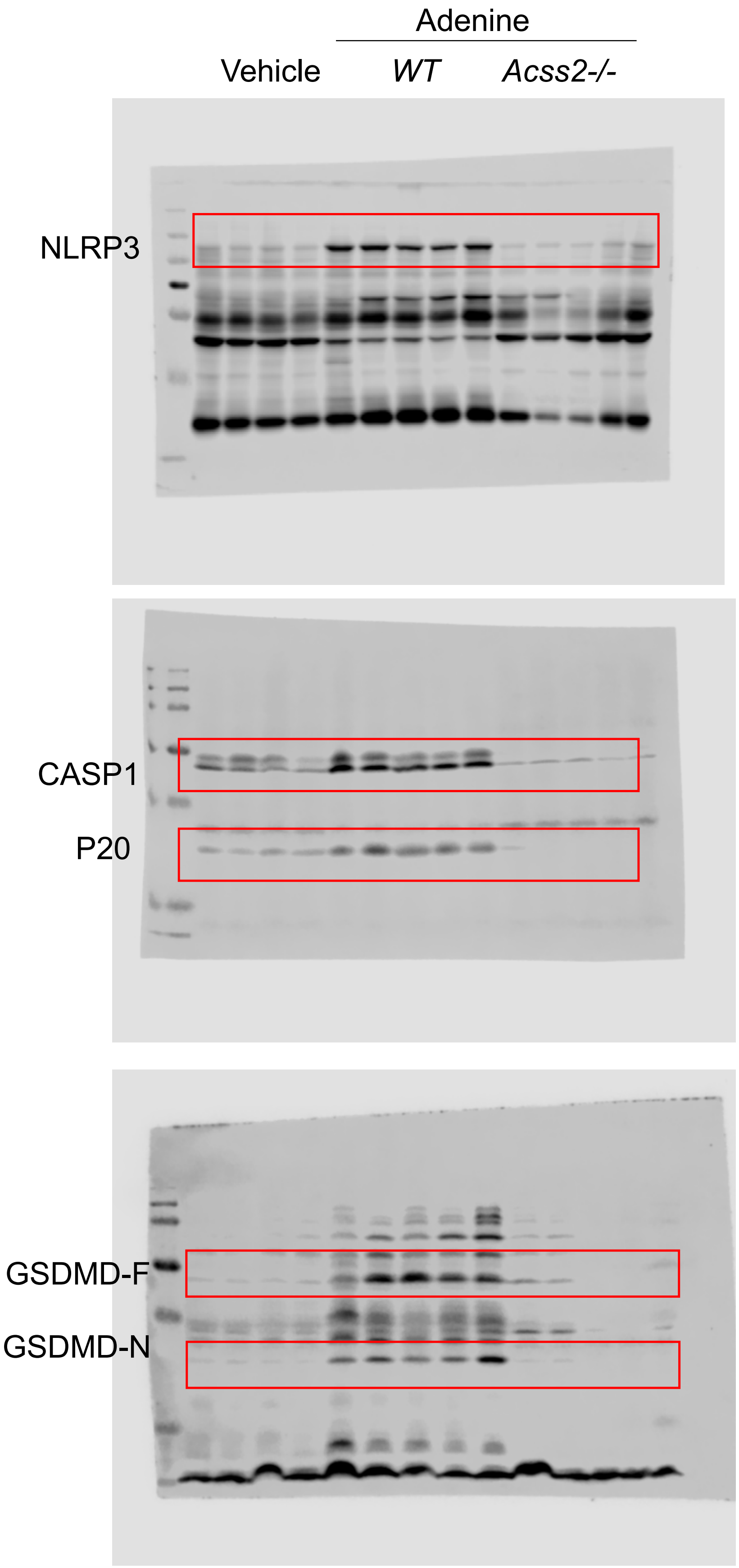

Full unedited blots for Figure 6J

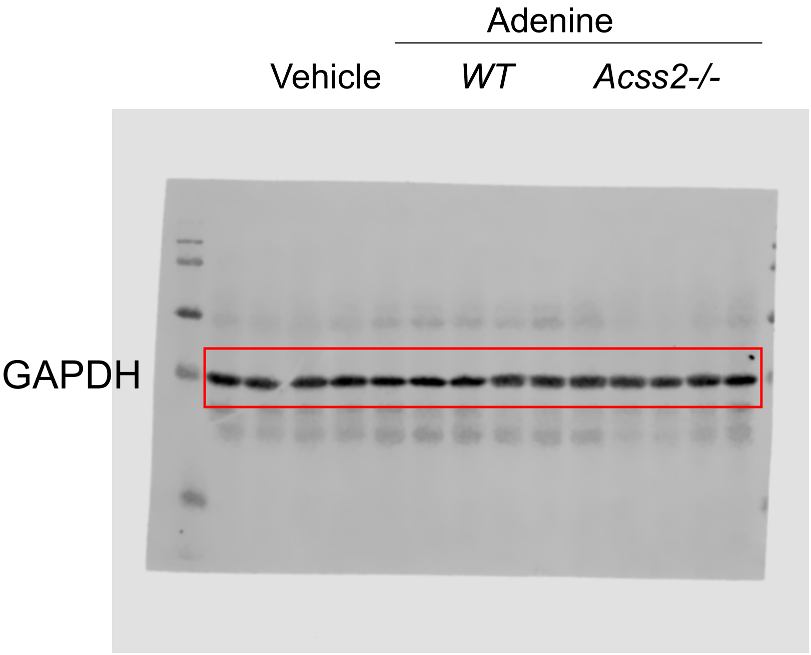

Full unedited blots for Figure 7H

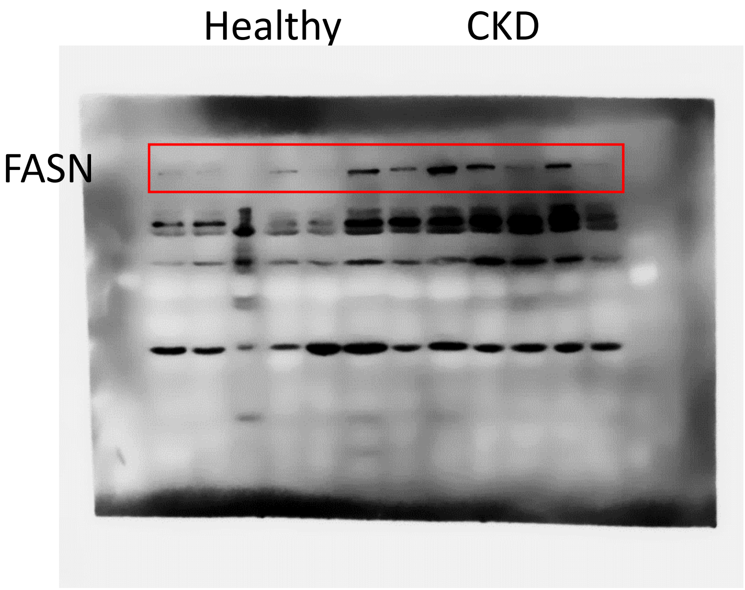

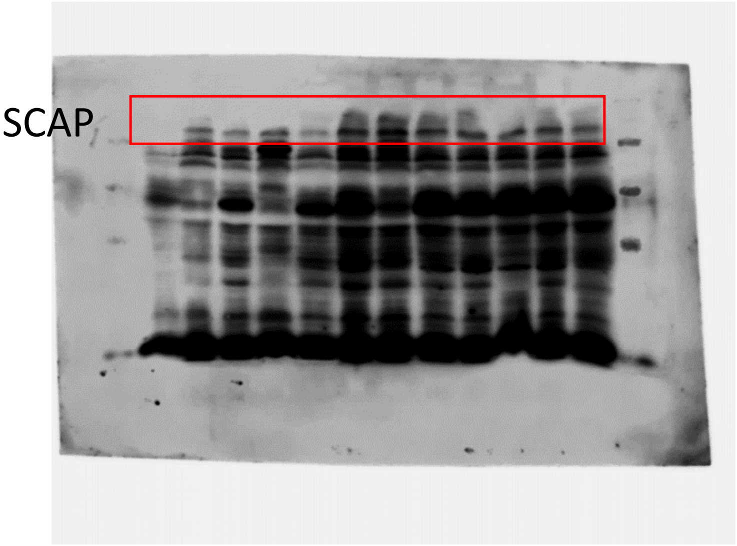

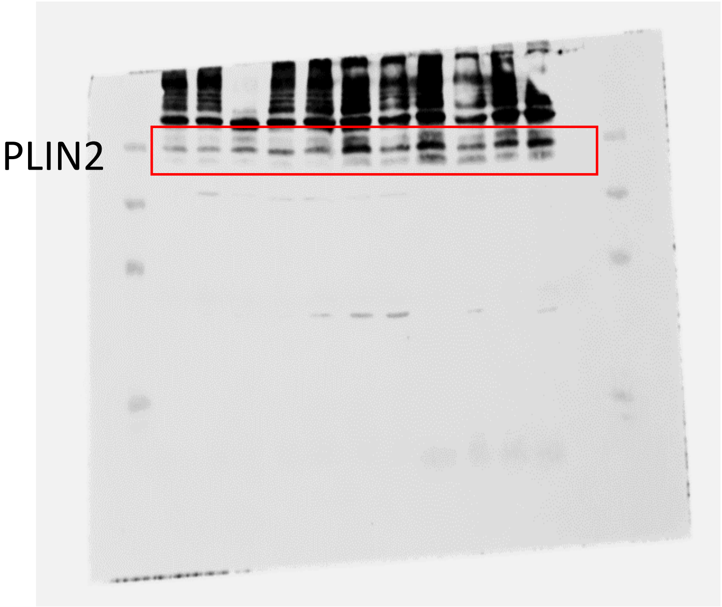

Full unedited blots for Figure 7H

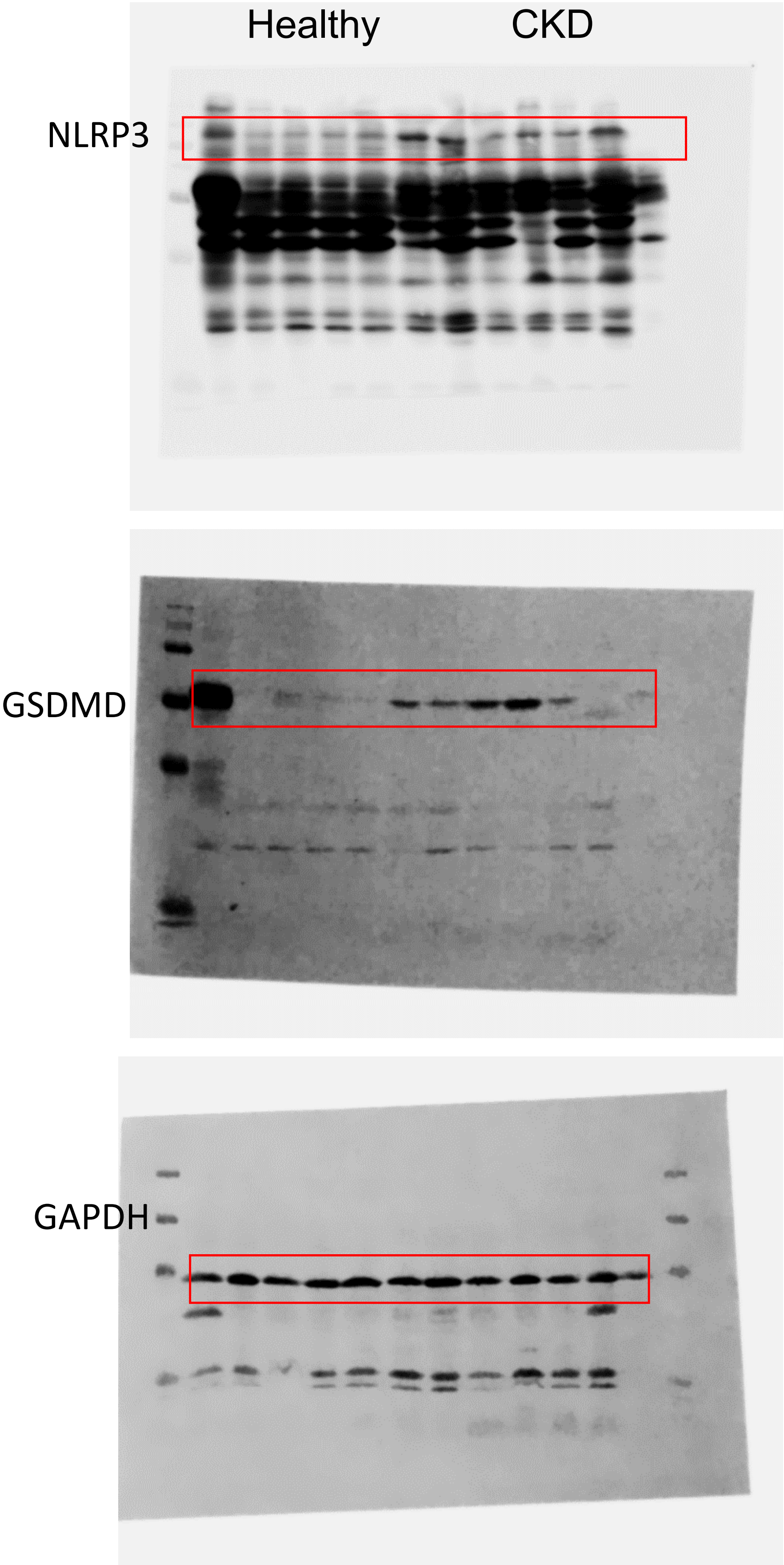

Full unedited blots for supplementary figure 1F

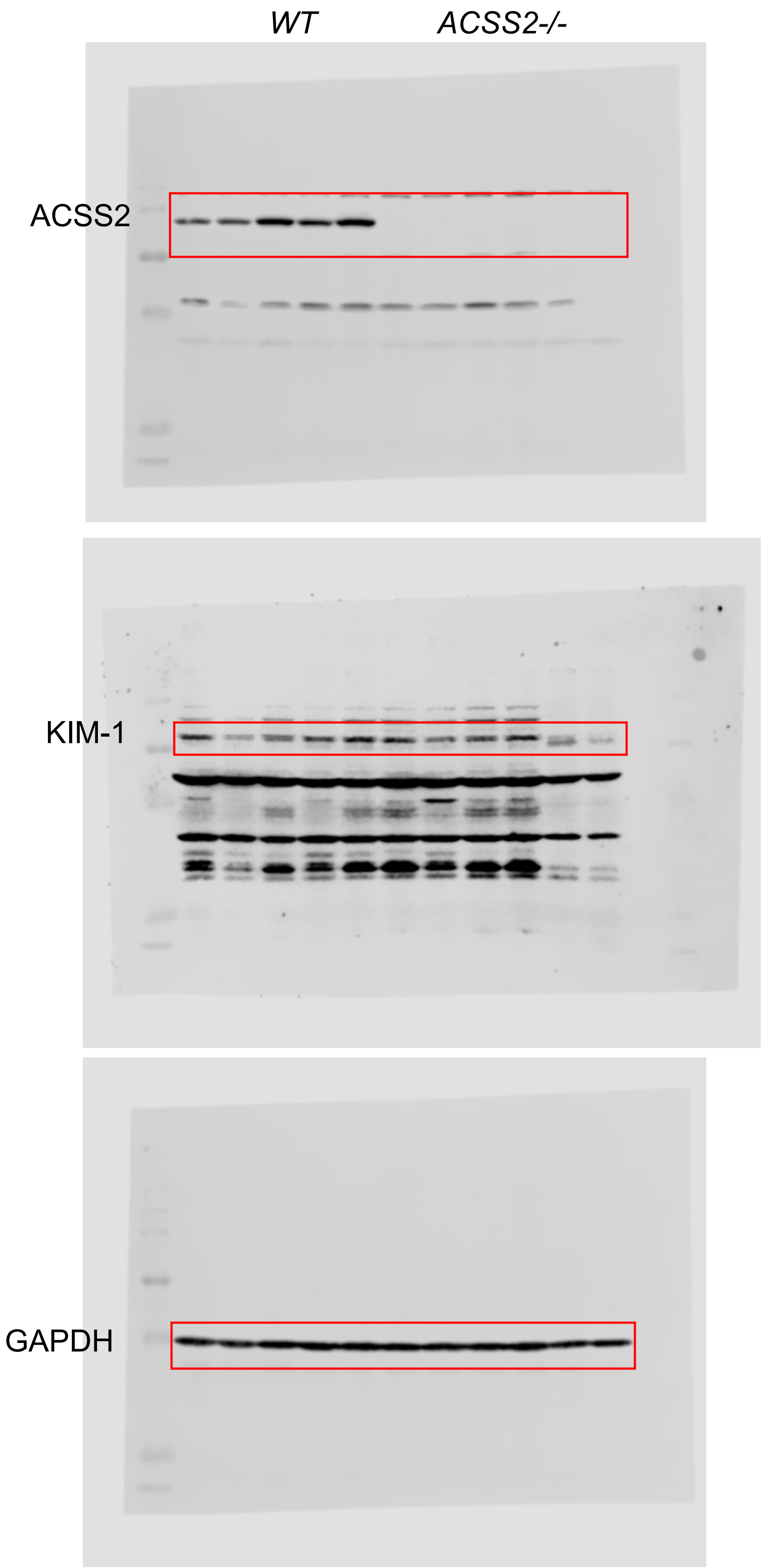

Full unedited blots for supplementary figure 2G

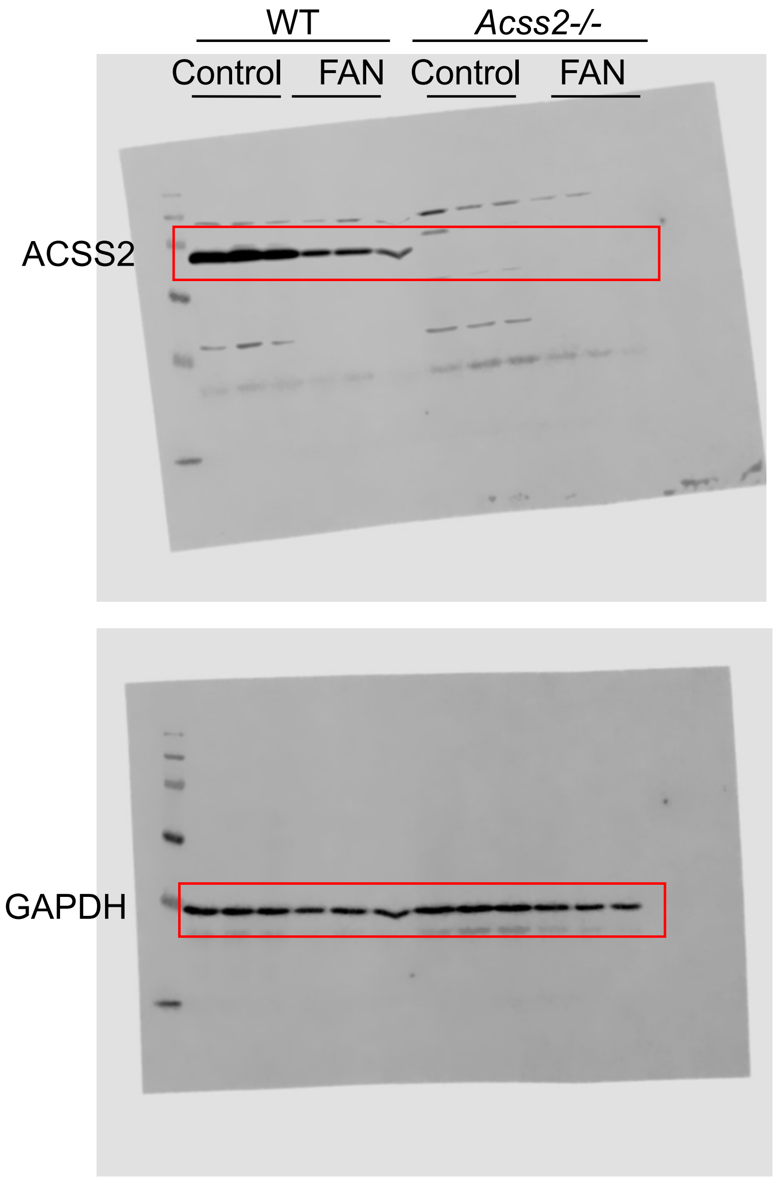

Full unedited blots for supplementary figure 2G

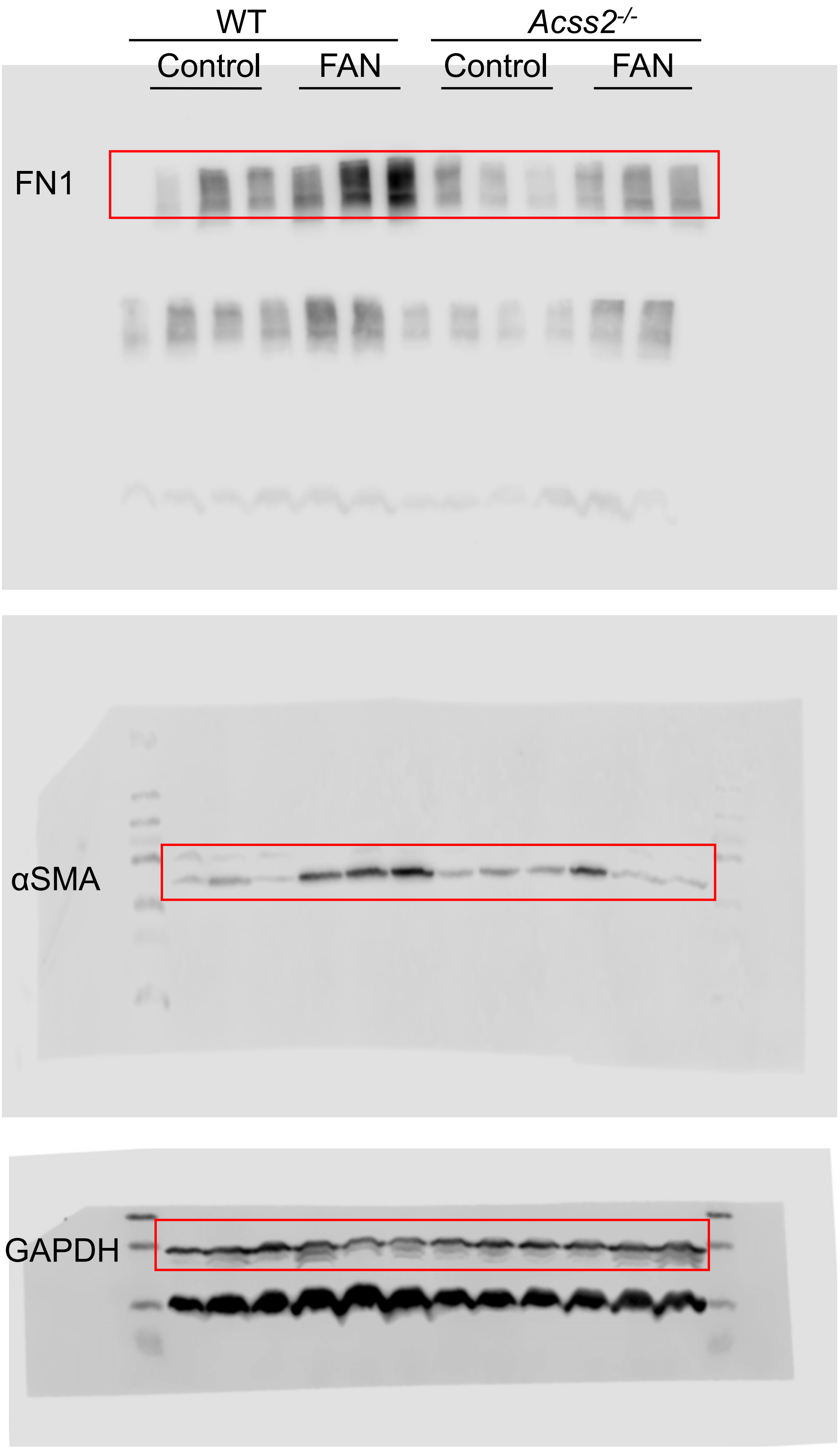

Full unedited blots for supplementary figure 2R

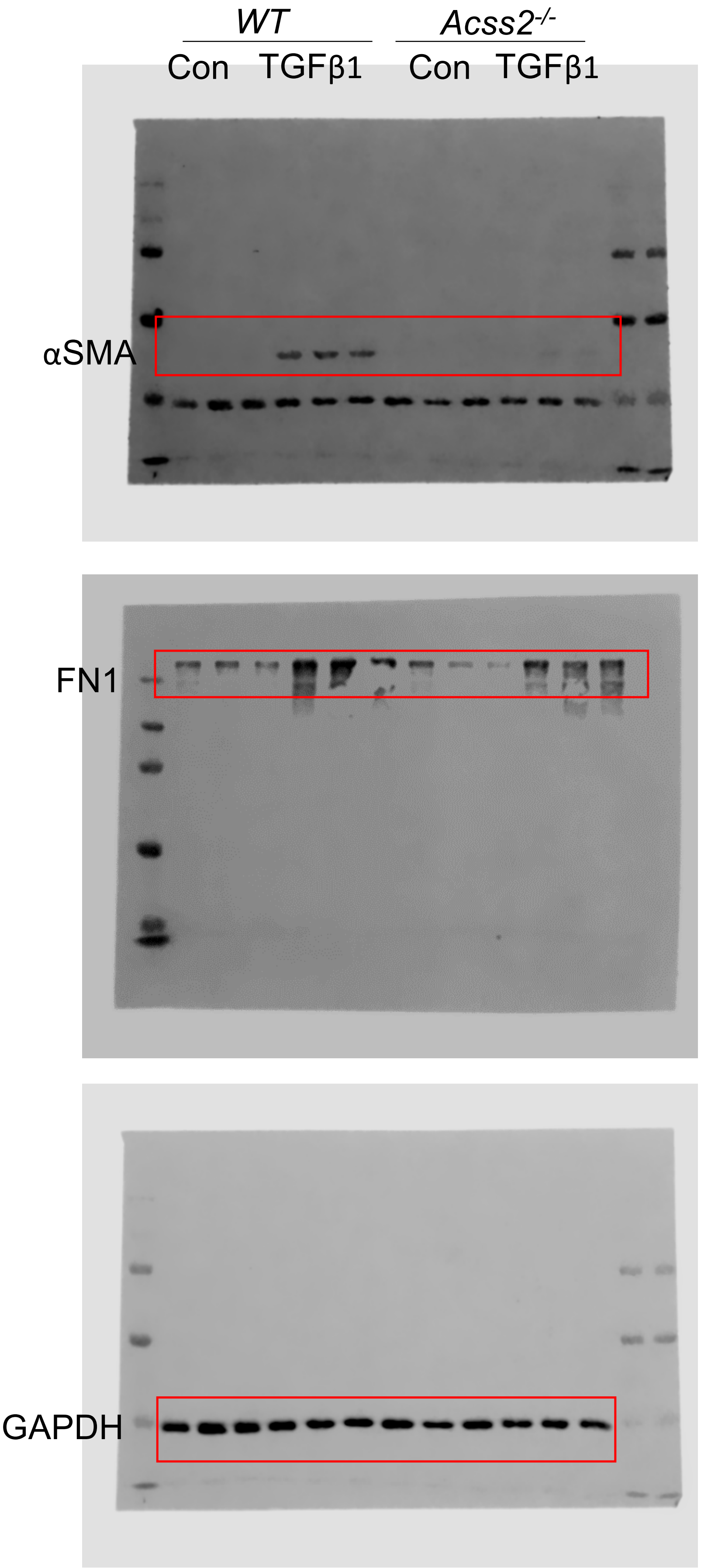

Full unedited blots for supplementary figure 4I

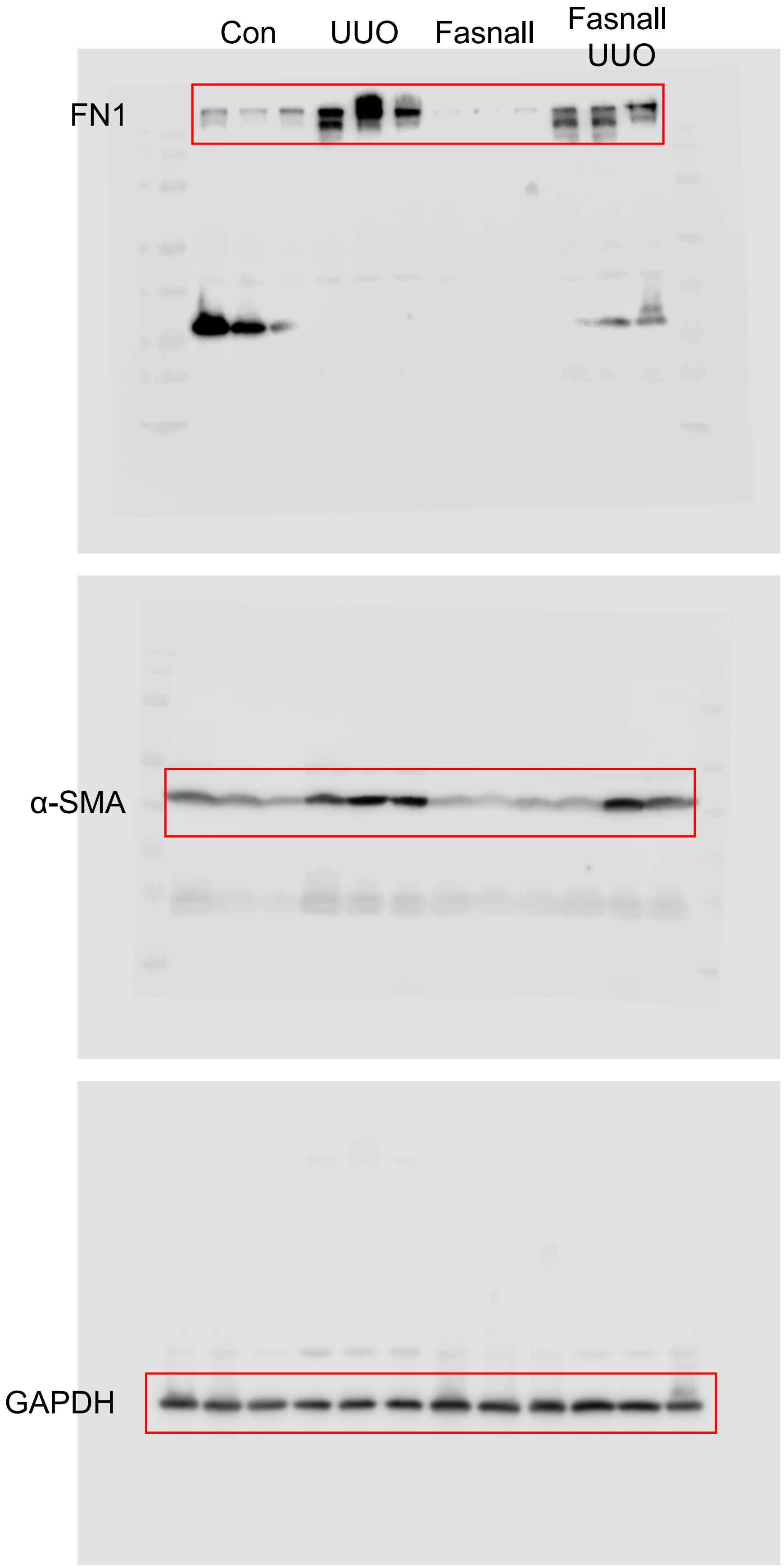

Full unedited blots for supplementary figure 4R

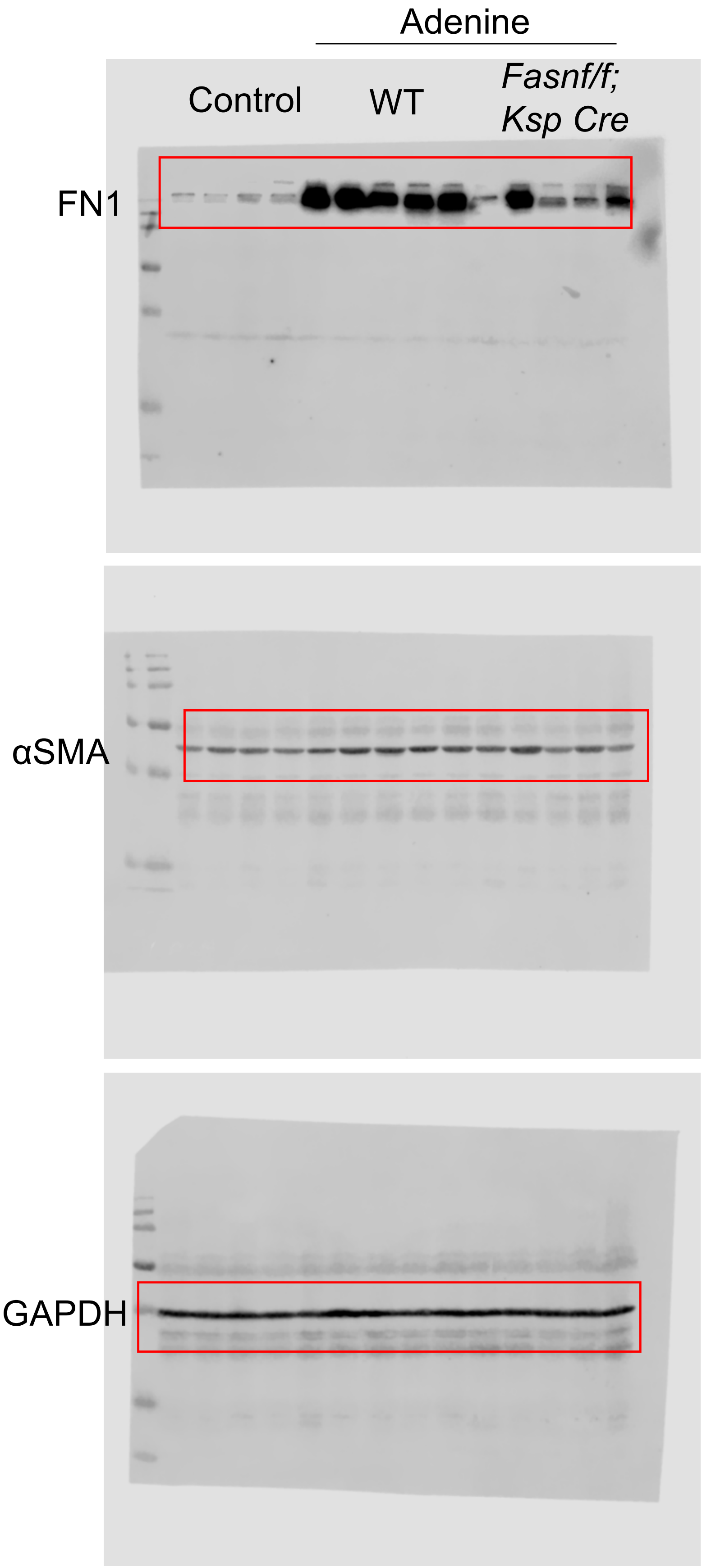

Full unedited blots for supplementary figure 5C

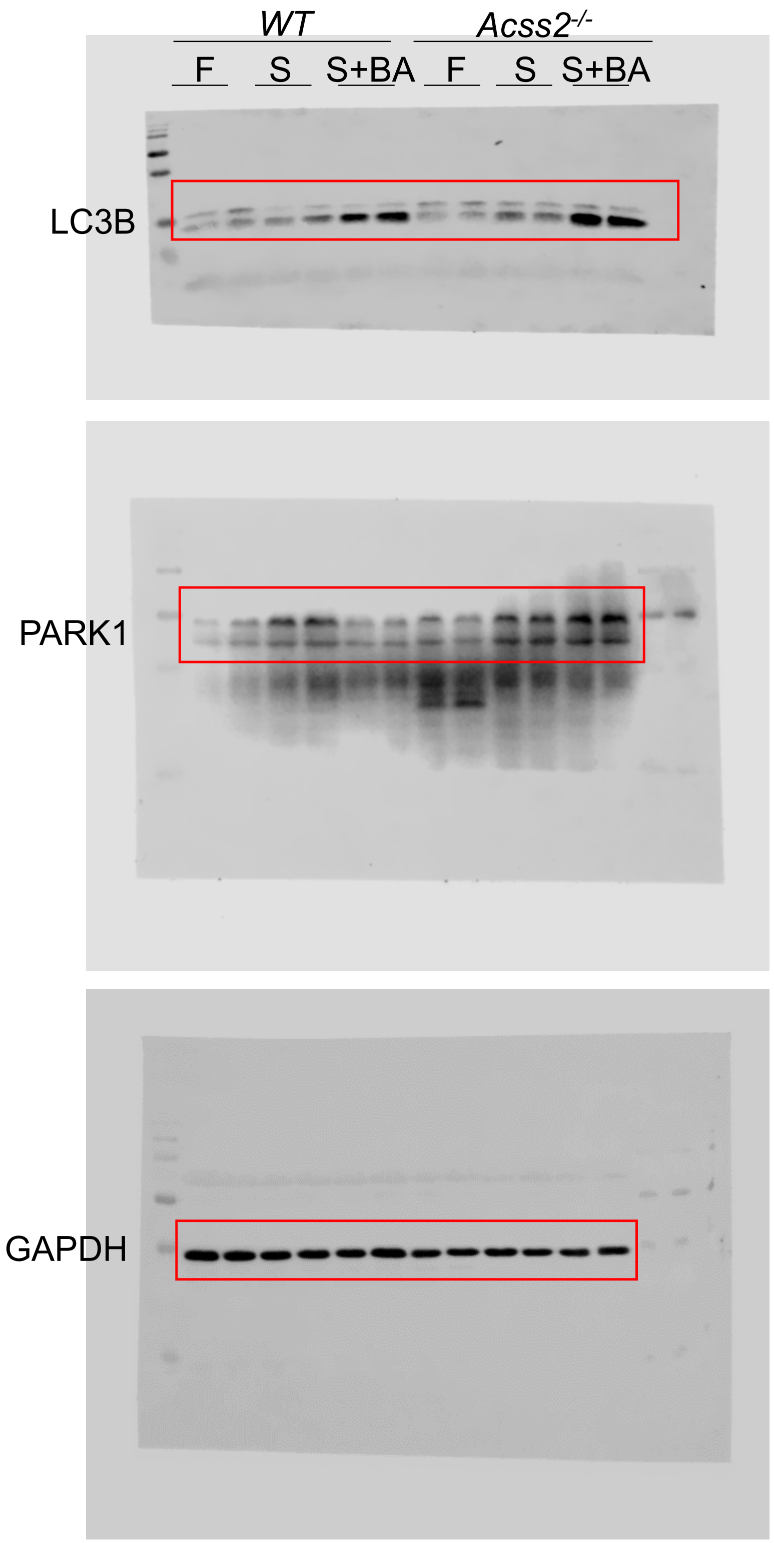

Full unedited blots for supplementary figure 6F

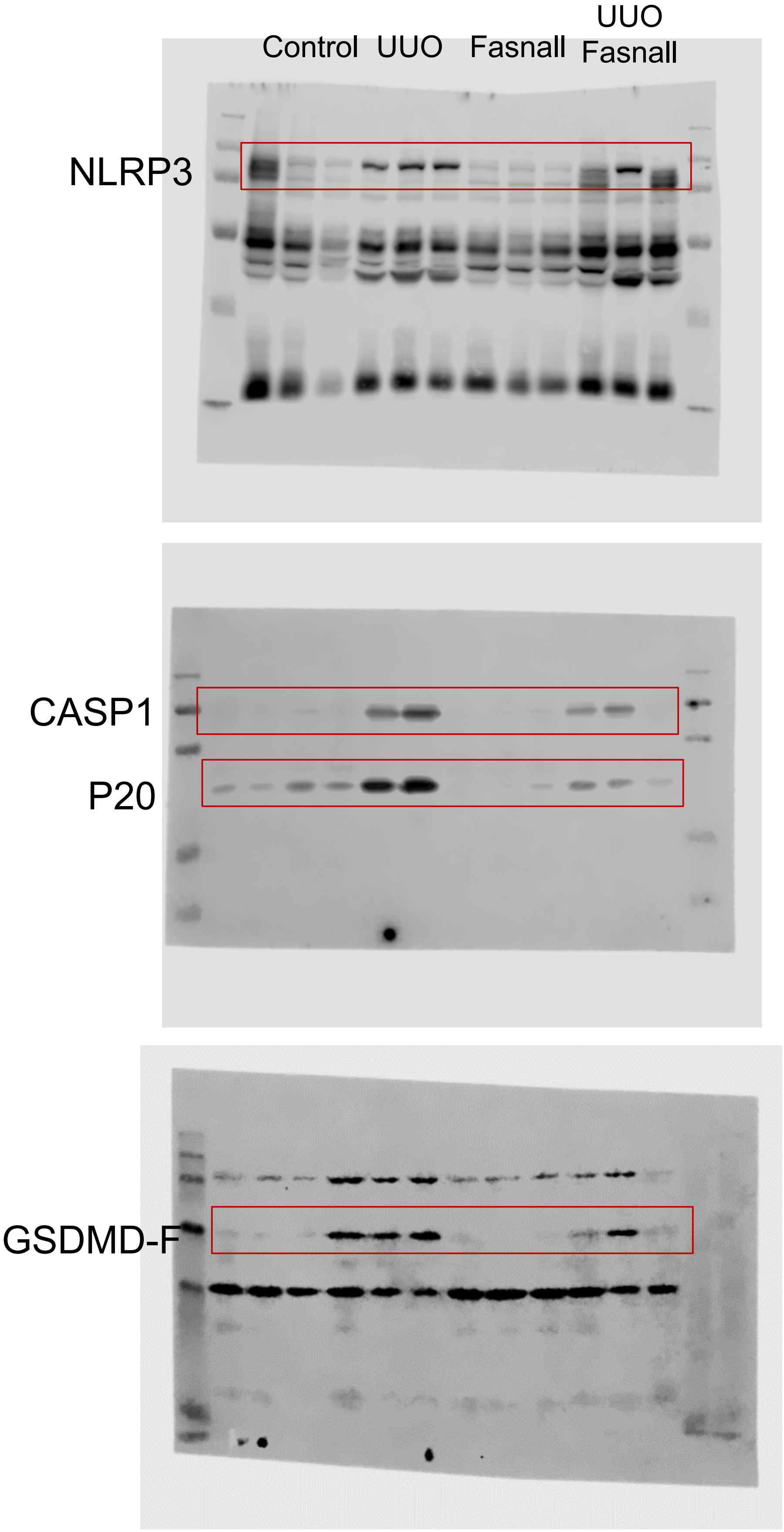

Full unedited blots for supplementary figure 6F

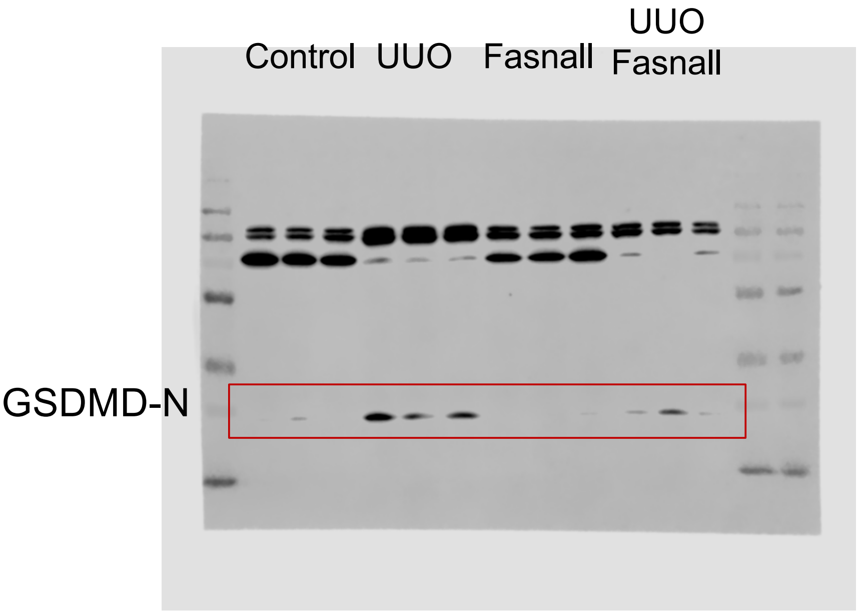

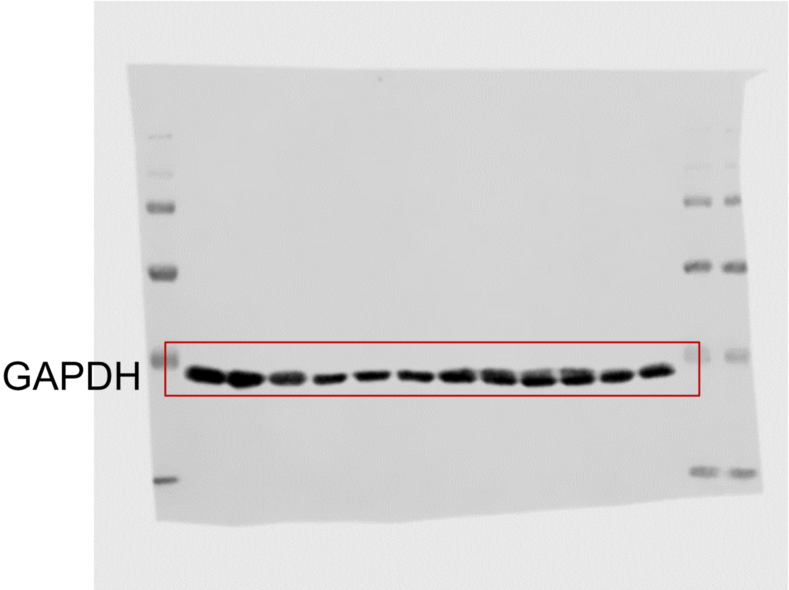

Full unedited blots for supplementary figure 6J

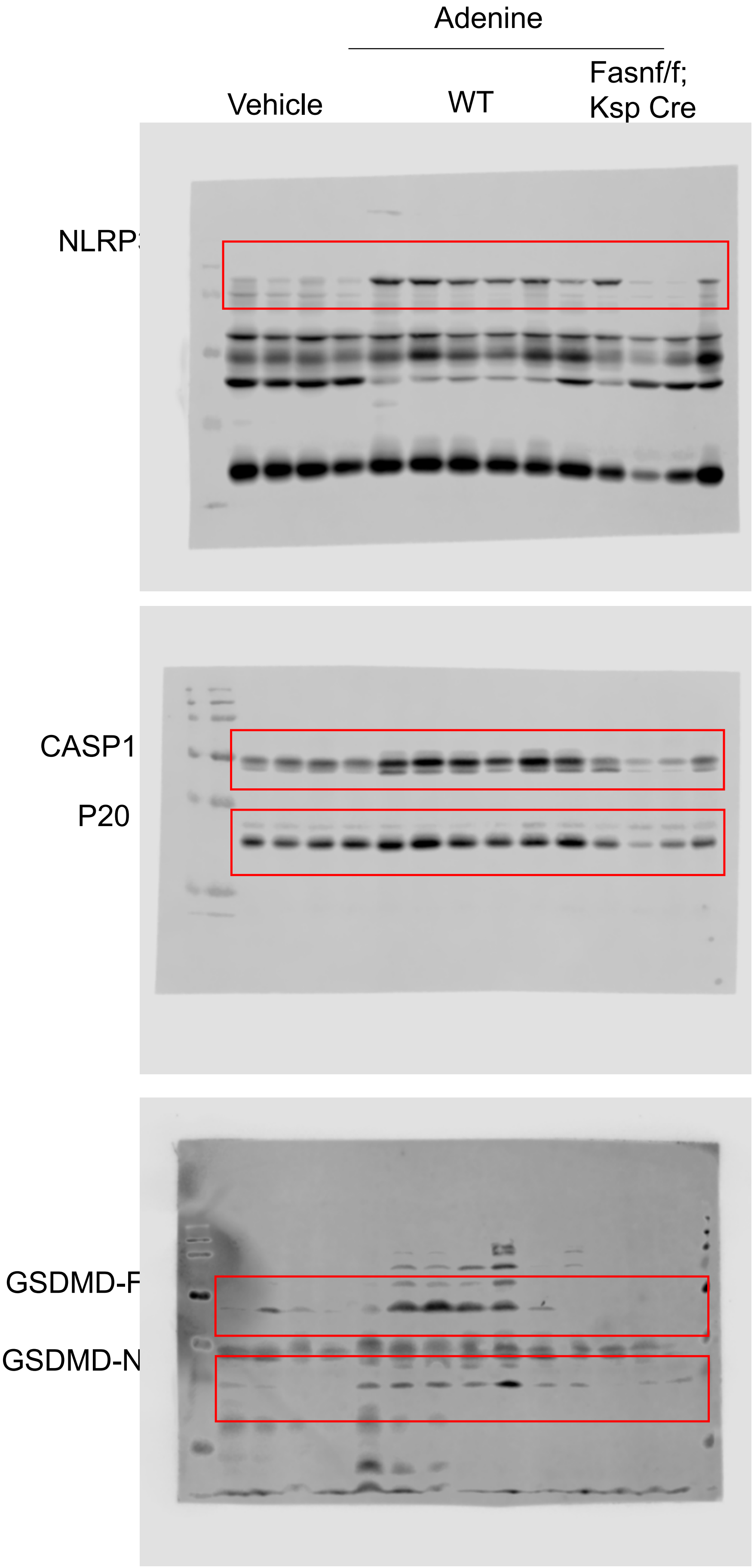

Full unedited blots for supplementary figure 6J

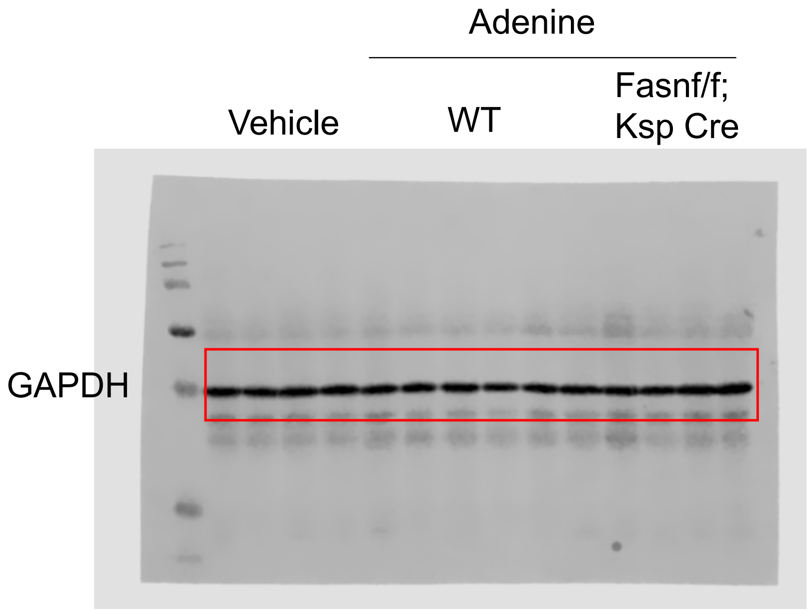

Supplement: Unedited blot and gel images [file jci-134-172963-s269.pdf]
